# Obstructive sleep apnea increases the risk of cardiovascular damage: a systematic review and meta-analysis of imaging studies

**DOI:** 10.1186/s13643-021-01759-6

**Published:** 2021-07-30

**Authors:** Mi Lu, Zhenjia Wang, Xiaojun Zhan, Yongxiang Wei

**Affiliations:** 1 Department of Otolaryngology Head & Neck Surgery, Beijing Anzhen Hospital, Capital Medical University, No.2 Anzhen Road, Beijing, 100029 China; 2 The Key Laboratory of Upper Airway Dysfunction-Related Cardiovascular Diseases, Beijing Institute of Heart, Lung and Blood Vessel Diseases, No.2 Anzhen Road, Beijing, 100029 China; 3 Department of Radiology, Beijing Hospital of Traditional Chinese Medicine, Capital Medical University, No. 23 Back Road of Art Gallery, Beijing, 100010 China; 4 Department of Otorhinolaryngology Head and Neck Surgery, Capital Institute of Pediatrics, No.2 Yabao Road, Beijing, 100020 China

**Keywords:** Obstructive sleep apnea, Atherosclerosis, Coronary artery calcium, Coronary plaque, Cardiac remodeling, Cardiac dysfunction

## Abstract

**Background:**

We aimed to perform a systematic review and meta-analysis of the association between obstructive sleep apnea (OSA) and cardiac as well as coronary impairment evaluated using imaging modalities. Finding of this study will provide more robust evidence regarding OSA-induced cardiovascular damage.

**Methods:**

We systematically searched through PubMed, EMBASE, and Cochrane library databases for relevant literatures on the association between OSA and cardiovascular damage evaluated using imaging modalities, and manually searched the references of selected articles for additional relevant articles. For each clinical parameter relevant to the meta-analysis, we first evaluated the methodological heterogeneity of the relevant studies and thereafter pooled the data together using fixed effect or random effect model. The difference in the relevant indices of cardiovascular damage between OSA patients and controls was evaluated using the standardized mean difference.

**Results:**

Of the 82 articles included in the final systematic analysis, 20 studies explored the association between OSA and coronary atherosclerosis. OSA patients had higher rate of coronary atherosclerosis assessed by coronary artery calcification score and plaque volume. Moreover, the severity of OSA and coronary atherosclerosis displayed a positive correlation. The rest of the studies (*n* = 62) evaluated cardiac alterations in OSA patients. According to the inclusion and exclusion criteria, 46 studies yielding 3082 OSA patients and 1774 controls were pooled for the meta-analysis. For left cardiac structure and function, OSA patients exhibited significantly wider left atrial diameter; higher left atrium volume index; wider left ventricular end-systolic diameter, left ventricular end-diastolic diameter, and left ventricular mass; higher left ventricular mass index; wider interventricular septum diameter and posterior wall diameter; and higher left ventricular myocardial performance index (all *p* < 0.05). In addition, compared with controls, left ventricular ejection fraction was significantly decreased in OSA patients (*p* = 0.001). For right cardiac structure and function, OSA patients displayed a significant increase in right ventricular diameter and right ventricular myocardial performance index (both *p* < 0.001). Finally, compared with controls, OSA patients displayed significant decrease in tricuspid annular plane systolic excursion and RV fractional area change (*p* = 0.001).

**Conclusion:**

Overall, this systematic review and meta-analysis provides imaging evidence in support that OSA patients are at a higher risk of developing coronary atherosclerosis and display cardiac remodeling and dysfunction.

**Supplementary Information:**

The online version contains supplementary material available at 10.1186/s13643-021-01759-6.

## Background

Obstructive sleep apnea (OSA) is a common disorder that affects nearly 1 billion adults worldwide [1]. Compared to general population, OSA is more prevalent in patients with cardiovascular disease, with a reported prevalence of 38 to 65% in coronary artery disease (CAD) patients and 12 to 55% in heart failure patients [2]. Substantial evidence summarizes that OSA can cause acute and long-term adverse implication for heart and vasculature by inducing intermittent hypoxia (IH), abrupt drop in intrathoracic pressure, sympathetic activation, and inflammatory disturbances [2–5]. Although apneic episode only occurs during sleep, pathophysiological perturbations induced by repeated apnea do not subside after waking up [5]. Thus, cardiovascular events can accumulate long after the cessation of apnea.

Cardiac imaging modalities can provide accurate assessment for coronary atherosclerosis and cardiac abnormalities. Early identification and evaluation of cardiovascular alterations in OSA patients may impact the risk stratification. Currently, evidence from the perspective of imaging is accumulating for exploring the relationship between OSA and cardiovascular damage [6–10]. These studies can be broadly divided into two categories, including OSA and coronary atherosclerosis, and OSA and cardiac alterations. However, these studies had a relatively small sample size and focused on specific populations (i.e., Turk, American, and Chinese). In addition, to the best of our knowledge, there is no systematic review and meta-analysis on the relationship between OSA and cardiac as well as coronary impairment. We aimed to summarize the association between OSA and cardiovascular damage assessed by imaging modalities. Findings of this study will provide more robust evidence regarding OSA-induced cardiovascular damage.

## Methods

This study was conducted in accordance with the Preferred Reporting Items for Systematic Review and Meta-Analysis (PRISMA) guidelines [11].

### Search strategies

We systematically searched through PubMed, EMBASE, and Cochrane library databases for relevant literatures on the association between OSA and cardiovascular damage evaluated by imaging modalities. Only articles written in English and published between January 1st, 2005, and June 30th, 2020 were considered. The following terms were used to search eligible articles: “sleep apnea, obstructive”, “sleep-disordered breathing”, “coronary atherosclerosis”, “coronary artery calcification”, “coronary artery calcium”, “coronary plaque”, “cardiac remodeling”, “ventricular function, left”, “ventricular dysfunction, left”, “left ventricular hypertrophy”, “ventricular function, right”, “ventricular dysfunction, right”, “computed tomography”, computed tomographic angiography”, “intravascular ultrasound”, “optical frequency domain imaging”, “echocardiography”, and “cardiac magnetic resonance imaging”. In addition, we manually searched the references of the selected studies to find other potential sources. An example of search strategy is provided in Supplementary materials.

### Study selection

Two investigators (Mi Lu and Zhenjia Wang) independently reviewed the titles and abstracts to identify relevant articles. A further screening was based on full-text articles to see whether they were eligible for inclusion. Any disagreement was resolved by discussion.

The inclusion criteria were as follows: (1) enrolled participants were adults; (2) all participants underwent polysomnography (PSG) or portable sleep monitoring; (3) English-language articles. In addition, articles that conducted meta-analysis must also meet the following criteria: (1) the study must include at least two separate groups, OSA group and non-OSA group; (2) non-OSA was defined as apnea hypopnea index (AHI) < 5; (3) the study must have reported values of the corresponding parameters in mean with standard deviation or median with range. The exclusion criteria were as follows: (1) duplicate reports; (2) reviews, case reports, or animal experiments; (3) patients with other sleep disorders, such as central sleep apnea, obesity hypoventilation syndrome, and periodic limb movement disorder; (4) patients who received or are receiving OSA treatment; (5) participants with major comorbidities, such as structural heart disease, cardiomyopathy, pulmonary hypertension, and chronic obstructive pulmonary disease.

### Data extraction

The search and retrieval of relevant articles was performed independently by two researchers (Mi Lu and Zhenjia Wang). For each included study, details of the first author, year of publication, country, sample size, participants’ age, gender, body mass index (BMI), mean AHI, OSA diagnostic method and criteria, history of hypertension, diabetes, hyperlipidemia, etc. were extracted. For articles included in meta-analysis, parameters reflecting cardiac morphology and function were also extracted. Any disagreements were resolved by consensus.

### Quality assessment

The Newcastle–Ottawa Scale (NOS) was used to assess the quality of included studies due to their case–control study design. The studies were assessed for the following criteria: selection (scale 0–4), comparability (scale 0–2), and exposure (scale 0–3). The quality score ranges from 0 to 9.

### Statistical analysis

A meta-analysis will be carried out if included studies are sufficiently homogeneous. Standardized mean difference (SMD) with 95% confidence intervals (CI) will be used to determine the difference in the relevant indices of cardiovascular damage between OSA patients and controls. Some studies stratified patients according to OSA severity (mild, moderate, or severe) and thus reported the corresponding data within each stratum. Two formulae were used to combine subgroups and calculate the overall means and standard deviations (see Supplementary materials). Heterogeneity was assessed using the *I*^2^ and chi-square tests, where *I*^2^ > 50% and a chi-square *p* < 0.05 indicated significant heterogeneity between studies. In such cases, the random effects model was used to generate pooled effects. By contrast, the fixed-effects model was instead used when there was an acceptable heterogeneity. We conducted subgroup analyses based on the age of 50 years and BMI of 30 kg/m^2^ when significant heterogeneity existed. Sensitivity analyses were conducted to test the robustness of the overall results using the leave-one-out method. Publication bias was assessed using a funnel plot. We used Egger tests to assess the asymmetry of the funnel plot. The trim-and-fill computation was used to estimate the effect of publication bias on the interpretation of the results. All analyses were performed using Stata/SE version 14.0 (StataCorp, College Station, Texas). Statistical significance was set at *p* < 0.05.

Finally, the Grading of Recommendations Assessment, Development, and Evaluation (GRADE) system was used to assess the quality of evidence for each outcome presented in the systematic review [12]. We recorded the overall quality of evidence as high, moderate, low, or very low.

## Results

**Fig. 1:**
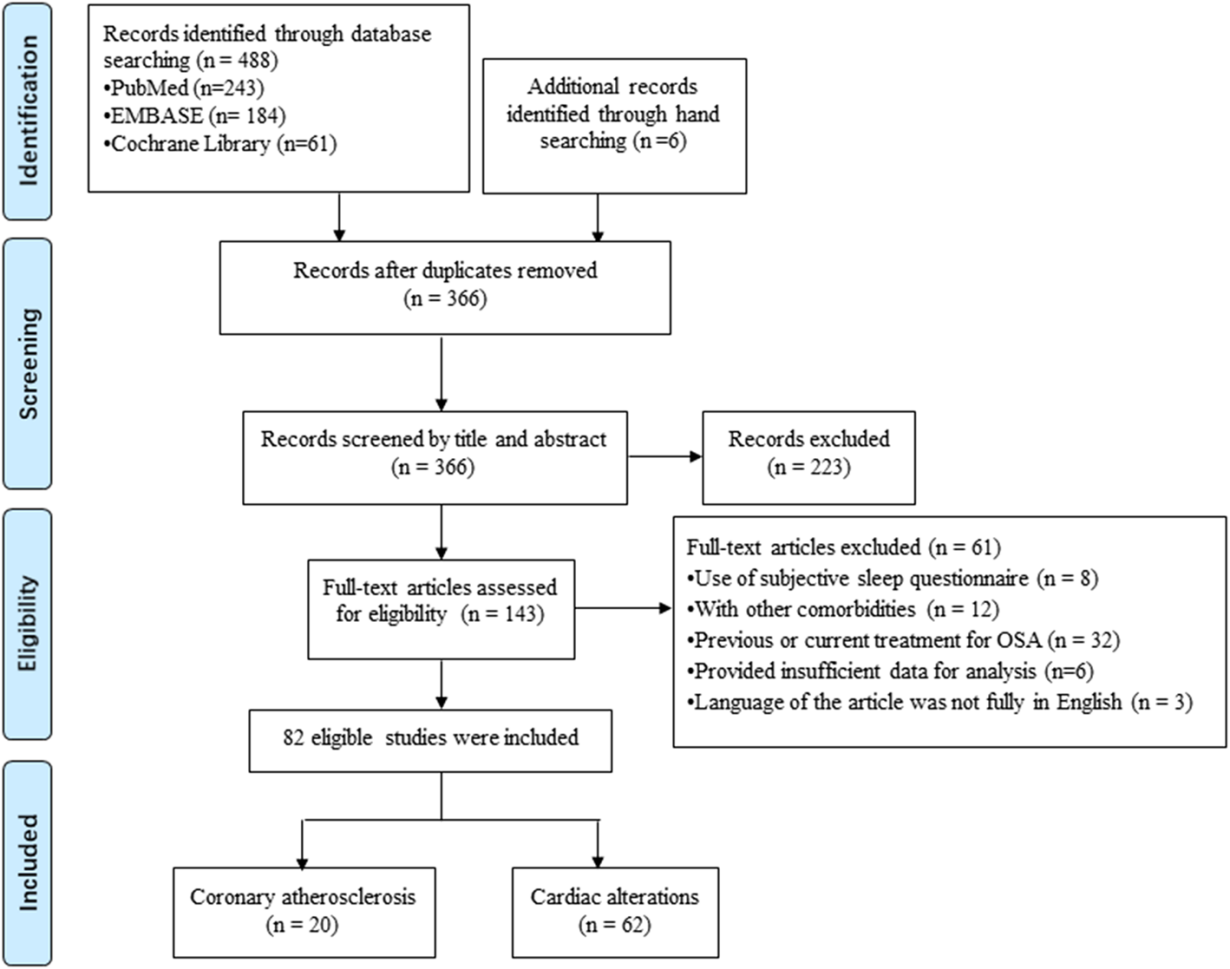
Flow diagram

The literature search yielded 366 citations. After removing duplicates, screening titles and abstracts, and reading the full text, only 82 articles were included in the final systematic review (Fig. 1). Of these, 20 studies explored the relationship between OSA and coronary atherosclerosis, assessed by coronary artery calcification (CAC) score, the presence and volume of coronary plaque. The rest 62 studies focused on cardiac abnormalities and myocardial injury in OSA patients. The certainty of evidence is shown in the Supplementary materials. The overall quality of evidence in this systematic review and meta-analysis is from very low to moderate due to risk of reporting bias and substantial heterogeneity.

### Obstructive sleep apnea and coronary artery calcification score

In this review, twelve articles evaluated the association between OSA and CAC score. The characteristics of the articles are shown in Table 1. All subjects included were free of overt cardiovascular diseases. Of these, 10 studies found that OSA was associated with CAC in basic models [13–22]; however, in some instances, the association was modestly attenuated and no longer significant after adjustment for confounding variables [15, 21, 22]. Another two studies by Kim et al. [14] and Luyster et al. [18] showed no significant relationship between OSA and CAC score after additional adjustment for BMI. In addition, Lutsey et al. [19] and Medeiros et al. [20] both demonstrated that the independent relationship between OSA and CAC score was only present in moderate-severe or severe OSA patients, while Bikov et al. [7] and Hamaoka et al. [8] found no correlation was observed between OSA and CAC score.

**Table 1:** Characteristics of the included studies on OSA and coronary artery calcification

First author	Year	Country	Simple size	Subgroup	Diagnosis criteria	Age, year	Male, %	BMI, kg/m2	Hypertension, %	Hyperlipidemia, %	Diabetes, %	ESS	AHI, events/h	OSA diagnostic methods	NOS
Sorajja [13]	2008	USA	202	Controls (48)	AHI < 5	46	30 (63)	31	20 (40)	23 (48)	2 (4)	NA	NA	PSG	8
				OSA (154)	AHI ≥ 5	51	111 (72)	35	76 (49)	99 (64)	14 (9)	NA	NA		
Kim [14]	2010	Korea	258	First quartile (64)	The quartile of AHI	44 ± 3	64 (100)	23 ± 2	4 (6)	NA	2 (3)	NA	0.2 ± 0.2	PSG	9
				Second quartile (64)		45 ± 3	64 (100)	24 ± 3	10 (16)	NA	2 (3)	NA	1.8 ± 0.8		
				Third quartile (65)		46 ± 3	65 (100)	24.8 ± 3	9 (14)	NA	5 (6)	NA	5.7 ± 1.8		
				Fourth quartile (65)		44 ± 3	65 (100)	26 ± 3	14 (22)	NA	2 (3)	NA	24 ± 15		
Kepez [15]	2011	Turkey	97	Snoring group (17)	AHI < 5	46.60 ± 4.59	10 (58.8)	28.25 ± 4.04	4 (23.5)	2 (12.5)	2 (11.8)	NA	NA	PSG	7
				Mild OSA (22)	5 ≤ AHI < 15	46.56 ± 9.54	16 (72.7)	27.61 ± 3.09	7 (31.8)	6 (27.3)	2 (9.1)	NA	NA		
				Moderate OSA (21)	15 ≤ AHI < 30	52.17 ± 7.20	16 (76.2)	29.18 ± 3.28	7 (33)	5 (23.8)	2 (9.5)	NA	NA		
				Severe OSA (37)	AHI ≥ 30	49.85 ± 10.60	22 (59.5)	30.99 ± 4.43	15 (40.5)	11 (29.7)	7 (18.9)	NA	NA		
Arik [16]	2013	Turkey	73	Controls (16)	AHI < 5	50 ± 10 48 ± 9	9 (56.3) 6 (40.2)	28.2 ± 4.8 30.9 ± 6.4	6 (38)	6 (38)	4 (25)	NA	2.4 ± 1.1 8.0 ± 2.0	PSG	7
				Mild OSA (14)	AHI 5–15				5 (36)	3 (21)	2 (14)	NA			
				Moderate OSA (19)	AHI 16–29	47 ± 9	13 (68.4)	28.4 ± 4.8	4 (21)	3 (16)	6 (32)	NA	22.5 ± 4.3		
				Severe OSA (24)	AHI ≥ 30	52 ± 10	15 (62.5)	32.2 ± 4.1	12 (50)	9 (38)	9 (38)	NA	45.3 ± 16.3		
Weinreich [17]	2013	Germany	1604	Controls (551)	AHI < 5	M 61.9 ± 6.9 F 60.8 ± 6.8	209 (37.9)	M 27.6 ± 3.6 F 26.2 ± 4.2	123 (58.9) 149 (43.6)	NA NA	27 (12.9) 30 (8.8)	NA	NA	Unattended portable monitoring	8
				Mild OSA (651)	5 ≤ AHI < 15	M 63.4 ± 7.4 F 64.6 ± 7.2	327 (50.2)	M 28.4 ± 3.5; F 27.7 ± 4.6	230 (70.3) 190 (58.6)	NA NA	42 (12.8) 30 (9.3)	NA	NA		
				Moderate OSA (288)	15 ≤ AHI ≤ 29	M 65.1 ± 7.3 F 66.1 ± 7.4	176 (61.1)	M 28.6 ± 3.7; F 28.8 ± 5.1	125 (71) 69 (61.6)	NA NA	26 (14.7) 13 (11.6)	NA	NA		
				Severe OSA (114)	AHI ≥ 30	M 65.9 ± 7.7 F 68.5 ± 5.7	79 (69.3)	M 30.2 ± 3.9; F 30.9 ± 4.1	63 (79.8) 25 (71.4)	NA NA	8 (10.1) 5 (14.3)	NA	NA		
Luyster [18]	2014	USA	252	Controls (61)	AHI < 5	59.1 ± 7.8	29 (48)	29.0 ± 5.8	43 (71)	45 (77)	10 (16)	NA	2.7 ± 1.1	Home sleep testing	9
				Mild OSA (97)	AHI 5–14	60.9 ± 7.6	48 (49)	29.9 ± 4.5	65 (67)	81 (83)	22 (23)	NA	9.0 ± 2.7		
				Moderate-severe OSA (94)	AHI ≥ 15	62.0 ± 6.5	64 (68)	30.4 ± 5.0	71 (75)	65 (69)	19 (20)	NA	26.9 ± 12.3		
Lutsey [19]	2015	USA	1465	Normal (510)	AHI < 5	66.9 ± 9.0	165 (32.3)	26.6 ± 5.0	NA	NA	71 (14.0)	NA	NA	PSG	9
				Mild OSA (478)	AHI 5–14	68.9 ± 9.2	214 (44.8)	28.9 ± 5.0	NA	NA	90 (19.0)	NA	NA		
				Moderate OSA (263)	AHI 15–29	69.1 ± 9.2	155 (58.9)	29.6 ± 5.3	NA	NA	63 (24.1)	NA	NA		
				Severe OSA (214)	AHI ≥ 30	67.9 ± 9.0	136 (63.6)	31.7 ± 6.0	NA	NA	51 (23.8)	NA	NA		
Medeiros [20]	2016	Brazil	214	Controls (132)	AHI < 5	55 (51–59)	0 (0)	27 (24–29)	92 (70)	94 (77)	32 (24)	9 (5–13)	1.9 (0.5–3.3)	Attended portable monitoring	7
				Mild OSA (61)	5 ≤ AHI < 15	59 (54–62)	0 (0)	29 (25–34)	50 (82)	45 (75)	15 (25)	9 (6–13)	8.3 (6.0–11.1)		
				Moderate/severe OSA (21)	AHI ≥ 15	58 (53–63)	0 (0)	32 (29–35)	20 (95)	14 (70)	6 (29)	9 (7–15)	16.9 (15.4–24.5)		
Seo [21]	2017	Korea	461	Normal (64)	AHI < 5	55.08 ± 7.65	428 (92.8)	25.57 ± 3.01	205 (44.5)	268 (58.1)	62 (13.4)	NA	25.46 ± 21.10	PSG	8
				OSA (397)	AHI ≥ 5										
Hamaoka [8]	2018	Japan	32	Mild-moderate OSA (15)	AHI 5–29	61.3 ± 12.4	11 (73.3)	26.3 ± 5.0	5 (30.0)	3 (20.0)	2 (13.3)	NA	20.7 ± 8.4	PSG	6
				Severe OSA (17)	AHI ≥ 30	65.0 ± 9.2	15 (88.2)	26.2 ± 5.3	9 (52.9)	5 (29.4)	4 (23.5)	NA	45.2 ± 11.9		
Shpilsky [22]	2018	USA	765	No/mild OSA (204)	AHI < 15	58 ± 7	159 (28)	30 ± 6	199 (36)	NA	43 (8)	NA	11 ± 11	Portable home monitoring	7
				Moderate/severe OSA (561)	AHI ≥ 15	61 ± 7	108 (53)	31 ± 6	104 (51)	NA	22 (11)	NA	26.1 ± 12.0		
Bikov [7]	2019	Hungary	41	Controls (19)	AHI ≤ 5	56 ± 9	3 (16)	26.3 ± 3.8	12 (63)	3 (16)	10 (53)	6.2 ± 3.7	1.8 ± 1.1	PSG or polygraphy	7
				OSA (22)	AHI > 5	62 ± 10	12 (55)	29.4 ± 5.7	34 (77)	22 (50)	8 (18)	7.2 ± 4.7	18.8 ± 16.3		

*AHI* apnea hypopnea index, *BMI* body mass index, *ESS* Epworth sleepiness scale, *NOS* Newcastle–Ottawa scale, *OSA* obstructive sleep apnea, *PSG* polysomnography

### Obstructive sleep apnea and coronary plaque burden

A total of 9 studies explored the relationship between OSA and coronary plaque characteristics by using invasive (*n* = 4) and non-invasive (*n* = 5) imaging modalities. The characteristics of the studies are summarized in Table 2. In a Canadian study of 19 patients who underwent intravenous ultrasound, Turmel et al. [23] reported that the coronary plaque volume was significantly larger in patients with moderate to severe OSA than those with no or mild OSA. Similar finding has been observed in a larger sample cohort. In this latter study, Tan et al. [24] found there was a significantly independent relationship between moderate to severe OSA and coronary plaque volume in the target coronary artery. Wada et al. [25] assessed plaque characteristics of the culprit lesion by intravenous ultrasound and found that sleep-disordered breathing was associated with larger plaque volume and greater ultrasound attenuation. Another Japanese cohort of 50 patients who underwent PSG and optical frequency-domain imaging again found that OSA patients had significantly larger lipid burden, thinner fibrous cap, greater macrophage accumulation, and more microchannels than those without OSA [9].

**Table 2:** Characteristics of the included studies on OSA and coronary plaque

First author	year	Country	Simple size	Subgroup	Diagnosis criteria	Age, years	Male (%)	BMI, kg/m2	Hypertension, %	Hyperlipidemia, %	Diabetes, %	ESS	AHI, events/h	OSA diagnosis methods	NOS
Turmel [23]	2009	Canada	19	Low AHI (7)	AHI < 15	57 ± 4	6 (86)	28.3 ± 2.9	NA	NA	NA	9.4 ± 3.7	11.9 ± 1.5	PSG	6
				High AHI (12)	AHI ≥ 15	63 ± 9	12 (100)	29.9 ± 5.7	NA	NA	NA	7.8 ± 6.7	39.4 ± 23.3		
Sharma [26]	2012	USA	81	Non OSA (32)	AHI < 10	54.1 ± 12.7	14 (43)	34.4	24 (77)	13 (42)	8 (26)	NA	7.5	PSG	5
				OSA (49)	AHI ≥ 10	59.6 ± 11.8	31 (63)	34.4	38 (84)	28 (62)	14 (31)	NA	42.2		
Kent [28]	2013	Ireland	29	Low AHI (15)	AHI < 15.5	45.1 ± 8.6	15 (100)	32.2 ± 4.1	0 (0)	0 (0)	0 (0)	7.7 ± 4.8	5.5 ± 5.5	Attended cardiorespiratory polygraphy	6
				High AHI (14)	AHI ≥ 15.5	44.6 ± 6.4	14 (100)	33.3 ± 5.6	0 (0)	0 (0)	0 (0)	13.2 ± 5.1	45.8 ± 20.1		
Tan [24]	2014	Singapore	93	No to mild OSA (61)	AHI ≤ 15	52.8 ± 9.0	55 (90.2)	24.9 ± 3.9	25 (41.0)	46 (75.4)	13 (21.3)	7.6 ± 4.5	NA	Portable diagnostic device	6
				Mod to severe OSA (32)	AHI > 15	57.5 ± 6.9	26 (81.2)	26.8 ± 4.2	23 (71.9)	29 (90.6)	11 (34.4)	9.4 ± 3.6	NA		
Hamaoka [8]	2018	Japan	32	Mild-moderate OSA (15)	AHI 5–29	61.3 ± 12.4	11 (73.3)	26.3 ± 5.0	5 (30)	3 (20)	2 (13.3)	NA	20.7 ± 8.4	Full PSG	5
				Severe OSA (17)	AHI ≥ 30	65.0 ± 9.2	15 (88.2)	26.2 ± 5.3	9 (52.9)	5 (29.4)	4 (23.5)	NA	45.2 ± 11.9		
Wada [25]	2018	Japan	289	No SDB (201)	3% ODI < 15	66.7 ± 11.3	161 (80.1)	24.2 ± 3.6	147 (73.1)	150 (74.6)	75 (37.3)	NA	/	Nocturnal pulse oximetry	6
				SDB (88)	3% ODI ≥ 15	69.1 ± 11.3	81 (92.1)	26.1 ± 4.6	72 (81.8)	73 (83.0)	39 (44.8)	NA	/		
Konishi [9]	2019	Japan	50	Non OSA (35)	AHI < 15	69.7 ± 8.9	27 (77)	23.6 ± 3.2	23 (66)	29 (83)	11 (31)	NA	7.2 ± 3.8	Standardized PSG	6
				OSA (15)	AHI ≥ 15	71.8 ± 10.4	10 (67)	23.6 ± 3.3	12 (80)	12 (80)	4 (27)	NA	30.9 ± 12.6		
Mo [29]	2019	Australia	119	Non-severe OSA (77)	AHI < 30	60 ± 11.4	52 (68)	31.7 ± 7	46 (59.7)	41 (53.2)	16 (20.8)	NA	29.5 ± 26.9	Laboratory PSG	6
				Severe OSA (42)	AHI ≥ 30	58.5 ± 11	30 (71)	32.8 ± 11.6	23 (54.8)	20 (47.6)	10 (23.8)	NA			
Umut [27]	2019	Turkey	214	Non-OSA (43)	Not mentioned	52.3 ± 6.4	33 (76.7)	31.2 ± 3.9	13 (30.2)	21 (48.8)	7 (16.3)	NA	4.0 ± 2.9	Attended PSG	8
				Mild OSA (51)		53.9 ± 6.7	34 (66.7)	32.6 ± 4.2	15 (29.4)	33 (64.7)	6 (11.8)	NA	9.8 ± 5.2		
				Moderate OSA (40)		55.2 ± 5.9	26 (65.0)	34.5 ± 6.9	15 (37.5)	26 (65.0)	9 (22.5)	NA	21.8 ± 4.1		
				Severe OSA (80)		54.9 ± 7.2	62 (77.5)	35.3 ± 5.6	30 (37.5)	42 (52.5)	19 (23.8)	NA	43.5 ± 12.9		

*AHI* apnea hypopnea index, *BMI* body mass index, *NOS* Newcastle–Ottawa scale, *ODI* Oxygen Desaturation Index, *OSA* obstructive sleep apnea, *PSG* polysomnography, *SDB* sleep-disordered breathing

Sharma et al. [26] and Umut et al. [27] both qualitatively assessed plaque burden using coronary computed tomography (CT) and found that the presence of non-calcified/mixed plaques was significantly higher in OSA patients compared to those without OSA. In addition, Kent et al. [28] found a positive relationship between OSA severity and total coronary plaque volume. Hamaoka et al. [8] furtherly reported a detailed association between OSA and coronary plaque volume with each CT value. They found that AHI was significantly correlated with low-attenuation plaque volume. In a recent study, Mo et al. [29] found that AHI and 3% oxygen desaturation index were both associated with significant coronary plaque burden.

### Obstructive sleep apnea and cardiac structure and function

A total of 60 studies explored the relationship between OSA and cardiac structure as well as function. Of these, 58 studies analyzed cardiac structure and function using echocardiography [30–87]. Cardiac magnetic resonance (CMR) [88] and single-photon emission computed tomography were used in 1 study each [89]. The main characteristics of the studies are shown in Table 3.

**Table 3:** Characteristics of the included studies on OSA and cardiac impairment

First author	Year	Country	Sample size	Subgroup	Diagnosis criteria	Age, years		Male (%)	BMI, kg/m^2^		Hypertension (%)		Hyperlipidemia (%)		Diabetes (%)	ESS		AHI, events/h	OSA diagnosis methods	NOS
**Echocardiography**																				
Arias [30]	2005	Spain	42	Control (15)	AHI < 5	48 ± 9	15 (100)			28.7 ± 4.7		0 (0)		NA	0 (0)	NA		3.9 ± 3.3	Respiratory recording device	7
				OSA (27)	AHI ≥ 10	52 ± 13	27 (100)			30.5 ± 4.0		0 (0)		NA	0 (0)	NA		44.0 ± 27.5		
Dursunoglu [31]	2005	Turkey	49	Control (20)	AHI < 5	43.5 ± 6.0	15 (75)			29.3 ± 2.4		0 (0)		NA	0 (0)	NA		5.2 ± 2.8	PSG	8
				Mild OSA (11)	AHI 5–14	46.0 ± 5.6	8 (73)			30.4 ± 4.0		0 (0)		NA	0 (0)	NA		25.3 ± 2.6		
				Moderate-severe OSA (18)	AHI ≥ 15	46.5 ± 4.9	14 (78)			30.6 ± 4.0		0 (0)		NA	0 (0)	NA		50.1 ± 11.6		
Dursunoglu [32]	2005	Turkey	67	Mild OSA (16)	AHI 5–14	46.0 ± 5.6	13 (81.3)			29.3 ± 2.4		4 (25)		NA	0 (0)	NA		5.2 ± 2.8	PSG	6
				Moderate OSA (18)	AHI 15–29	46.5 ± 4.9	15 (83.3)			30.4 ± 4.0		8 (44.4)		NA	0 (0)	NA		25.3 ± 2.6		
				Severe OSA (33)	AHI ≥ 30	48.1 ± 6.5	28 (84.8)			30.6 ± 3.7		26 (61.9)		NA	0 (0)	NA		50.1 ± 11.6		
Kasikcioglu [33]	2005	Turkey	28	Control (14)	AHI < 5	51.8 ± 12.9	14 (100)			27.9 ± 2.5		0 (0)		0 (0)	0 (0)	NA		1.7 ± 1.1	PSG	7
				OSA (14)	AHI > 15	49.7 ± 11.6	14 (100)			28.7 ± 2.9		0 (0)		0 (0)	0 (0)	NA		32.9 ± 7.1		
Tanriverdi [34]	2006	Turkey	64	Control (24)	AHI < 5	51.9 ± 5.2	19 (79.2)			29.4 ± 3.9		0 (0)		0 (0)	0 (0)	NA		3 ± 1.5	PSG	8
				OSA (40)	AHI ≥ 5	51.3 ± 9	32 (80)			29.8 ± 5.3		0 (0)		0 (0)	0 (0)	NA		25.3 ± 11.4		
Kasikcioglu [35]	2007	Turkey	20	Control group (10)	AHI < 5	45 ± 9	10 (100)			27.7 ± 2.6		0 (0)		0 (0)	0 (0)	NA		2.1 ± 1.0	PSG	7
				Patient group (10)	AHI > 30	42 ± 6	10 (100)			30.6 ± 3.2		0 (0)		0 (0)	0 (0)	NA		43.8 ± 11.7		
Ott [36]	2007	USA	41	Without OSA (18)	AHI < 5	45 ± 2	18 (100)			32.3 ± 0.9		0 (0)		0 (0)	0 (0)	NA		2 ± 0.4	PSG	8
				Moderate to severe OSA (23)	AHI ≥ 15	45 ± 3	23 (100)			33.7 ± 0.8		0 (0)		0 (0)	0 (0)	NA		50 ± 7.0		
Tavil [37]	2007	Turkey	41	Control (21)	AHI < 5	49 ± 5	12 (57.1)			29 ± 6		NA		NA	0 (0)			2 ± 2	PSG	8
				OSA (20)	AHI ≥ 5	50 ± 7	11 (55.0)			30 ± 7		NA		NA	0 (0)			31 ± 29		
Bayram [38]	2008	Turkey	46	Control (18)	AHI < 5	41.9 ± 11.5	14 (77.8)			27.9 ± 2.7		0 (0)		0 (0)	0 (0)	NA		2.6 ± 0.8	PSG	8
				OSA (28)	AHI ≥ 15	44.8 ± 10.5	23 (82.1)			29.7 ± 5.3		0 (0)		0 (0)	0 (0)	NA		62.3 ± 21.6		
Kim [39]	2008	Korea	62	None (24)	AHI < 5	52 ± 1	24 (100)			27 ± 1		3 (13)		NA	2 (8)	NA		2.0 ± 0.3	PSG	8
				Mild to moderate (18)	5 ≤ AHI ≤ 30	52 ± 1	18 (100)			27 ± 1		2 (11)		NA	5 (28)	NA		16.1 ± 1.2		
				Severe (20)	AHI > 30	51 ± 1	20 (100)			27 ± 1		3 (15)		NA	3 (15)	NA		45.5 ± 3.2		
Oliveira [40]	2008	Brazil	106	Control (50)	AHI < 5	52.3 ± 8.5	20 (40)			27.8 ± 4.5		18 (36.0)		NA	4 (8.0)	NA		2.6 ± 1.4	PSG	9
				OSA (56)	AHI ≥ 5	52.9 ± 10.6	29 (51.8)			29.4 ± 6.3		25 (44.6)		NA	4 (7.1)	NA		30.3 ± 23.1		
Bayram [41]	2009	Turkey	46	Control (18)	AHI < 5	41.9 ± 11.5	14 (77.8)			27.9 ± 2.7		NA		0 (0)	0 (0)	NA		2.6 ± 0.8	PSG	8
				OSA (28)	AHI ≥ 15	44.8 ± 10.5	23 (82.1)			29.7 ± 5.3		NA		0 (0)	0 (0)	NA		62.3 ± 21.6		
Baguet [42]	2009	France	130	Group A (65)	RDI < 37	48 ± 10	52 (80)			25.6 ± 3		0 (0)		0 (0)	NA	NA		N/A	PSG or polygraphy	5
				Group B (65)	RDI > 37	49 ± 10	57 (88)			27.5 ± 3.5		0 (0)		0 (0)	NA	NA				
Haruki [43]	2009	USA	49	control (20)	AHI < 5	36 ± 6	19 (95)			23.4 ± 2.9		1 (5)		3 (15)	0 (0)	NA		2.2 ± 1.4	PSG	8
				OSA (29)	AHI ≥ 5	40 ± 8	29 (100)			27.6 ± 4.3		9 (31)		22 (76)	3 (10)	NA		34.7 ± 23.1		
Kepez [44]	2009	USA	107	Without OSA (22)	AHI < 5	46.4 ± 4.6	13 (59.1)			27.6 ± 4.0		4 (18.2)		NA	NA	NA		2.5 ± 2.0	PSG	7
				Mild to moderate OSA (45)	5 ≤ AHI < 30	48.8 ± 8.2	33 (73.3)			28.4 ± 3.4		16 (35.6)		NA	NA	NA		15.0 ± 13.0		
				Severe OSA (40)	AHI ≥ 30	48.6 ± 9.2	24 (60)			31.5 ± 4.9		14 (35)		NA	NA	NA		46.0 ± 42.0		
Lee [45]	2009	China	51	Control (16)	AHI < 5	39 ± 7.8	13 (82)			27 ± 3.8		3 (19)		0 (0)	NA	NA		2 ± 1.6	PSG	8
				Mild OSA (20)	AHI 5–20	45 ± 9.5	17 (85)			28 ± 3.3		2 (10)		0 (0)	NA	NA		11 ± 4.5		
				Moderate to severe OSA (15)	AHI > 20	46 ± 11.9	15 (100)			31 ± 5.1		8 (53)		0 (0)	NA	NA		48 ± 21		
Tugcu [46]	2009	Turkey	71	Controls (30)	AHI < 5	54 ± 10	22 (73.3)			30.10 ± 3.65		0 (0)		12 (40)	0 (0)	2.6 ± 2.29		1.46 ± 0.68	PSG	7
				OSA (41)	AHI ≥ 15	56 ± 12	32 (78.0)			31.38 ± 4.97		0 (0)		21 (51.2)	0 (0)	19.37 ± 4.3		38.84 ± 21.80		
Tomiyama [47]	2009	Japan	164	None (14)	AHI < 5	44 ± 10	12 (85.7)			23.6 ± 2.8		0 (0)		0 (0)	0 (0)	8 ± 5		2.1 ± 1.2	PSG	7
				Mimo (65)	5 ≤ AHI < 30	46 ± 11	59 (90.8)			25.0 ± 3.1		0 (0)		0 (0)	0 (0)	10 ± 4		19.7 ± 6.1		
				Severe (85)	AHI ≥ 30	49 ± 11	78 (91.8)			27.0 ± 3.7		0 (0)		0 (0)	0 (0)	11 ± 5		51.2 ± 18.1		
Cioffi [48]	2010	Italy	157	Controls (20)	AHI < 5	56 ± 15	16 (80)			29 ± 6		10 (50)		12 (60)	2 (10)	NA		3.4 (1–10)	PSG	7
				Mild OSA (51)	AHI 5–15	60 ± 13	41 (80)			29 ± 5		36 (71)		28 (55)	11 (21)	NA		10 (6–15)		
				Moderate/severe OSA (86)	AHI > 15	63 ± 12	73 (85)			31 ± 5		67 (78)		51 (59)	15 (17)	NA		33 (23–46)		
Tugcu [49]	2010	Turkey	53	Control (26)	AHI < 5	54 ± 10	19 (73.1)			29.6 ± 3.6		0 (0)		NA	0 (0)	NA		2 ± 1	PSG	7
				OSA (27)	AHI ≥ 15	54 ± 10	24 (88.9)			31.1 ± 5.1		0 (0)		NA	0 (0)	NA		40 ± 22		
Varol [50]	2010	Turkey	64	Control (18)	AHI < 5	44.8 ± 11.6	13 (72)			29.2 ± 4.8		0 (0)		NA	0 (0)	NA		2.1 ± 1.6	PSG	8
				Mild to moderate (25)	5 ≤ AHI ≤ 30	51.2 ± 8.7	19 (76)			29.9 ± 4.3		0 (0)		NA	0 (0)	NA		15.8 ± 7.4		
				Severe (21)	AHI > 30	48.9 ± 9.3	18 (85)			32.2 ± 3.6		0 (0)		NA	0 (0)	NA		60.7 ± 24.5		
Cicek [51]	2011	Turkey	90	Group A (26)	AHI ≤ 5	49.4 ± 12.3	13 (50)			21.1 ± 2.3		12 (46.2)		6 (23.1)	4 (15.4)	7.5 ± 4.7		2.4 ± 1.5	PSG	7
				Group B (20)	5 < AHI < 15	59.3 ± 9.5	9 (45)			23.5 ± 3.1		4 (20.0)		4 (20)	2 (10.0)	7.4 ± 4.3		8.4 ± 3.6		
				Group C (20)	15 ≤ AHI < 30	60.1 ± 13.5	6 (30)			26.5 ± 4.2		8 (40.0)		5 (25)	5 (25.0)	8.1 ± 4.2		25.5 ± 4.2		
				Group D (24)	AHI ≥ 30	57.3 ± 16.2	6 (25)			30.5 ± 6.4		15 (62.5)		9 (37.5)	8 (33.3)	8.8 ± 5.9		62.9 ± 23.6		
Altintas [52]	2012	USA	40	Mild OSA (7)	AHI 5–14	38.3 ± 6.1	4 (57.1)			27.5 ± 5.1		1 (14.3)		NA	0 (0)	NA		8.5 ± 2.2	PSG	7
				Moderate OSA (13)	AHI 15–29	42.7 ± 9.1	10 (76.9)			31.4 ± 4.6		4 (30.8)		NA	0 (0)	NA		21.5 ± 3.8		
				Severe OSA (20)	AHI ≥ 30	48.9 ± 7.4	17 (85.0)			31.5 ± 4.9		10 (50)		NA	0 (0)	NA		50.5 ± 15.3		
Balci [53]	2012	Turkey	94	Control (33)	AHI < 5	41.6 ± 11.6	16 (48.5)			26.3 ± 1.4		0 (0)		0 (0)	0 (0)	NA		3.2 ± 1.9	PSG	8
				Mild to moderate (30)	5 ≤ AHI < 30	42.5 ± 11.2	18 (60.0)			26.9 ± 2.4		0 (0)		0 (0)	0 (0)	NA		14.2 ± 14.6		
				Severe (31)	AHI ≥ 30	45.7 ± 10.3	16 (51.6)			27.3 ± 2.3		0 (0)		0 (0)	0 (0)	NA		66.3 ± 39.9		
Butt [54]	2012	UK	80	Control (40)	AHI < 5	46 ± 9	30 (75)			32 ± 6		0 (0)		0 (0)	0 (0)	NA		3 ± 2	PSG	8
				OSA (40)	AHI ≥ 15	50 ± 10	33 (82.5)			34 ± 8		0 (0)		0 (0)	0 (0)	NA		39 ± 22		
Cho [55]	2012	Korea	45	Control (20)	AHI < 5	47.2 ± 7.1	N/A			27.9 ± 1.7		0 (0)		NA	0 (0)	6.67 ± 1.11		N/A	PSG	6
				OSA (25)	AHI ≥ 15	43.5 ± 11.3				28.0 ± 3.4		0 (0)		NA	0 (0)	13.6 ± 3.4		19.7 ± 11.6		
Hammerstingl [56]	2012	Germany	183	Control (29)	AHI < 5	55.7 ± 15.8	17 (58.6)			30.1 ± 5.5		12 (44.4)		7 (24.1)	76 (49.4)	NA		2.3 ± 1.3	PSG	8
				OSA (154)	AHI > 5	61.7 ± 12.4	109 (70.8)			31.1 ± 5.8		1 (3.4)		53 (34.4)	21 (13.6)	NA		35.9 ± 28.4		
Kim [57]	2012	Korea	49	Control (24)	AHI < 5	48.42 ± 7.45	17 (79.8)			27.45 ± 2.41		0 (0)		0 (0)	0 (0)	NA		2.94 ± 1.44	PSG	8
				OSA (25)	AHI ≥ 5	43.48 ± 11.32	20 (80)			28.1 ± 3.1		0 (0)		0 (0)	0 (0)	NA		19.66 ± 11.64		
Oliveira [58]	2012	Brazil	106	Control (50)	AHI < 5	52.3 ± 8.5	20 (40.0)			27.8 ± 4.5		18 (36)		NA	4 (8)	NA		2.6 ± 1.4	PSG	8
				OSA (56)	AHI > 20	52.9 ± 10.6	29 (51.8)			29.4 ± 6.3		25 (44.6)		NA	4 (7.1)	NA		30.3 ± 23.1		
Pressman [59]	2012	USA	54	None/mild OSA (14)	AHI < 15	43 ± 13	3 (20)			37 ± 6		7 (50)		NA	2 (14)	NA		4.9 ± 4	PSG	5
				Moderate/severe (40)	AHI ≥ 15	45 ± 10	18 (45)			42 ± 9		24 (60)		NA	10 (25)	NA		50 ± 28		
Yang [60]	2012	China	295	Control (75)	AHI < 5	59.8 ± 1.1	61 (81.3)			26.32 ± 4.57		0 (0)		NA	0 (0)	NA		2.9 ± 2.0	PSG	8
				OSA (220)	AHI > 5	58.4 ± 0.7	179 (81.4)			27.39 ± 5.74		0 (0)		NA	0 (0)	NA		20.0 ± 5.6		
Aslan [61]	2013	Turkey	80	Group 1 (43)	AHI < 15	44.1 ± 10.9	31 (72.1)			28.48 ± 4.2		0 (0)		0 (0)	0 (0)	NA		5.3 ± 4.5	PSG	5
				Group 2 (37)	AHI ≥ 15	46.0 ± 9.4	34 (91.9)			31.41 ± 4.8		0 (0)		0 (0)	0 (0)	NA		49.2 ± 24.8		
Hammerstingl [62]	2013	Germany	82	Group 1 (29)	AHI 5–14	61.8 ± 13.0	21 (72.4)			28.9 ± 4.9		13 (44.8)		9 (31)	1 (3.4)	8.5 ± 4.1		9.0 ± 2.8	PSG	5
				Group 1 (24)	AHI 15–30	66.3 ± 10.5	13 (54.1)			30.4 ± 4.5		15 (62.5)		12 (50)	4 (16.6)	9.4 ± 4.5		22.0 ± 4.4		
				Group 1 (29)	AHI > 30	62.5 ± 10.7	17 (58.6)			32.9 ± 6.3		16 (55.1)		9 (31)	5 (17.2)	13.6 ± 4.9		61.7 ± 22.7		
Usui [63]	2013	Japan	74	Mild to moderate OSA (52)	5 ≤ AHI < 30	41.0 ± 13.1	52 (100)			24.2 ± 2.7		0 (0)		0 (0)	0 (0)	NA		17.2 ± 6.9	PSG	6
				Severe OSA (22)	AHI ≥ 30	47.0 ± 13.5	22 (100)			24.5 ± 3.0		0 (0)		0 (0)	0 (0)	NA		44.7 ± 10.3		
Vitarelli [64]	2013	Italy	77	Control (35)	AHI < 5	45.1 ± 12.2	13 (37.1)			26.8 ± 4.3		0 (0)		0 (0)	0 (0)	NA		3.8 ± 1.1	PSG	8
				Mild OSA (19)	5 ≤ AHI < 30	48.3 ± 8.2	7 (36.8)			27.5 ± 5.4		0 (0)		0 (0)	0 (0)	NA		15.4 ± 2.2		
				Severe OSA (23)	AHI ≥ 30	47.4 ± 8.1	9 (39.1)			28.3 ± 6.5		0 (0)		0 (0)	0 (0)	NA		59.4 ± 9.3		
Araz [65]	2014	Turkey	98	Group 1 (31)	AHI < 5	49.8 ± 10.9	20 (29.9)			29.5 ± 7.3		0 (0)		NA	0 (0)	3 ± 2		1.5 ± 1.4	PSG	7
				Group 2 (67)	AHI ≥ 5	49.7 ± 12.7	47 (70.1)			34.6 ± 8.3		0 (0)		NA	0 (0)	10 ± 4		52.6 ± 32.3		
Chen [66]	2014	China	79	Control group (14)	AHI < 15	47 ± 8	9 (64.3)			24.1 ± 3.4		7 (50)		4 (28.6)	0 (0)	NA		8.6 ± 3.8	PSG	5
				OSAS group (65)	AHI ≥ 15	49 ± 10	54 (83.1)			26.9 ± 3.6		32 (49.2)		17 (26.2)	0 (0)	NA		45.4 ± 19.5		
Danica [67]	2014	Serbia	203	Controls (78)	AHI < 5	48.8 ± 10.2	36 (46.2)			24.9 ± 2.8		5 (6.4)		51 (65.4)	0 (0)	NA		37.2 ± 21.7	PSG	8
				Patients (125)	AHI ≥ 5	51.6 ± 10.7	91 (72.8)			31.6 ± 5.6		69 (55.6)		94 (75.2)	0 (0)	NA				
Sun [68]	2014	China	186	Control group (50)	AHI < 5	62.2 ± 10.8	37 (74.0)			29.66 ± 4.22		0 (0)		NA	0 (0)	NA		RDI 26 ± 19	PSG	7
				OSA group (136)	AHI ≥ 5	63.3 ± 10.6	89 (65.4)			30.94 ± 4.15		0 (0)		NA	0 (0)	NA		RDI 14 ± 6		
Cil [69]	2015	Turkey	74	Control (30)	AHI < 5	43.03 ± 10.89	25 (83.3)			30.8 ± 4.6		8 (26.7)		3 (10)	4 (13.3)	NA		1.35 ± 2.94	PSG	8
				OSA (44)	AHI ≥ 5	49.8 ± 11.5	30 (68.2)			34.0 ± 6.7		19 (43.2)		9 (20.4)	6 (13.6)	NA		28.05 ± 28.82		
Imai [70]	2015	Germany	206	Mild to moderate OSA (139)	5 ≤ AHI < 30	45 ± 12	115 (83)			23.7 ± 2.8		0 (0)		NA	0 (0)	NA		16.0 ± 6.7	PSG	5
				Severe OSA (67)	AHI ≥ 30	52 ± 11	61 (91)			25.7 ± 2.4		0 (0)		NA	0 (0)	NA		49.4 ± 14.9		
Sforza [71]	2015	France	405	Non SDB (31)	AHI < 5	68.9 ± 0.7	5 (17)			24.1 ± 3.4		13 (41.9)		14 (45.2)	0 (0)	5.0 ± 3.3		3.3 ± 1.2	Respiratory monitoring	8
				Mild SDB (129)	5 < AHI < 15	68.8 ± 0.7	44 (34)			24.5 ± 3.3		47 (36.4)		53 (41.1)	4 (3.1)	5.5 ± 3.4		9.8 ± 3.2		
				Moderate SDB (135)	15 < AHI < 30	68.9 ± 0.8	55 (41)			25.3 ± 3.7		57 (42.2)		56 (41.5)	4 (3.0)	5.9 ± 3.6		21.3 ± 4.1		
				Severe SDB (110)	AHI ≥ 30	68.8 ± 0.8	62 (56)			26.9 ± 3.6		53 (48.6)		32 (29.4)	8 (7.3)	6.4 ± 3.7		46.0 ± 4.7		
Wang [72]	2015	China	108	Control (30)	AHI < 5	45 ± 6	21 (71)			25 ± 4		0 (0)		NA	0 (0)	NA		2.7 ± 1.2	PSG	8
				Mild (26)	5 ≤ AHI < 15	48 ± 8	18 (69)			26 ± 4		0 (0)		NA	0 (0)	NA		10.5 ± 3.2		
				Moderate (29)	5 ≤ AHI < 30	45 ± 8	8 (26)			27 ± 3		0 (0)		NA	0 (0)	NA		18.7 ± 5.6		
				Severe (23)	AHI ≥ 30	46 ± 6	6 (28)			27 ± 4		0 (0)		NA	0 (0)	NA		57.2 ± 2.6		
Akyol [73]	2016	Turkey	116	Mild OSA (26)	5 ≤ AHI < 15	44.1 ± 10.2	16 (61.5)			28.5 ± 4.9		0 (0)		NA	0 (0)	NA		8.3 ± 2.9	PSG	5
				Moderate OSA (41)	15 ≤ AHI < 30	44.2 ± 10	30 (73.2)			29.7 ± 3.3		0 (0)		NA	0 (0)	NA		23.1 ± 3.5		
				Severe OSA (49)	AHI ≥ 30	46.4 ± 11.2	33 (67.3)			31 ± 2.7		0 (0)		NA	0 (0)	NA		51.9 ± 17.3		
Altiparmak [74]	2016	Turkey	94	Control (42)	AHI < 5	46 ± 7	28 (66.7)			25.8 ± 3.3		6 (14.3)		NA	5 (11.9)	NA		N/A	PSG	9
				OSA (52)	AHI ≥ 5	49 ± 10	35 (67.3)			26.5 ± 2.1		16 (30.8)		NA	10 (19.2)	NA				
Altıparmak [75]	2016	Turkey	66	Control (35)	AHI < 5	43.0 ± 6.4	23 (74.2)			26.2 ± 3.2		0 (0)		NA	0 (0)	NA		N/A	PSG	8
				OSA (31)	AHI ≥ 5	45.5 ± 6.6	25 (71.4)			26.7 ± 2.1		0 (0)		NA	0 (0)	NA		45.4 ± 28.1		
Güvenç [76]	2016	Turkey	67	Control (26)	AHI < 5	47 ± 13	19 (73)			26.01 ± 3.53		10 (3.8)		NA	0 (0)	NA		N/A	PSG	7
				OSA (41)	AHI ≥ 5	48 ± 9	27 (66)			32.02 ± 4.6		13 (31.6)		NA	4 (10.5)	NA		Moderate 23.0 ± 4.5; severe 53.4 ± 18.5		
Korcarz [77]	2016	USA	544	AHI < 5 (468)	AHI < 5	46 ± 7	222 (47)			28.8 ± 5.9		NA		NA	9 (2)	NA		1.1 ± 1.3	PSG	8
				AHI 5–14.9 (76)	AHI 5–14.9	49 ± 8	52 (68)			31.0 ± 5.2		NA		NA	1 (1)	NA		8.7 ± 2.9		
Li [78]	2016	China	100	Control (31)	AHI < 5	46.8 ± 5.4	19 (61.3)			24.86 ± 2.78		0 (0)		NA	0 (0)	NA		1.72 ± 1.01	PSG	7
				Mild (24)	AHI 5–15	47.3 ± 6.1	15 (62.5)			26.40 ± 3.12		0 (0)		NA	0 (0)	NA		12.72 ± 2.03		
				Moderate (25)	AHI 16–30	47.9 ± 7.9	15 (60)			26.83 ± 3.55		0 (0)		NA	0 (0)	NA		24.01 ± 3.56		
				Severe (20)	AHI > 30	48.5 ± 5.4	12 (60)			27.97 ± 3.59		0 (0)		NA	0 (0)	NA		40.78 ± 5.02		
Ozkececi [79]	2016	Turkey	90	Without OSA (30)	AHI < 5	46.4 ± 14	14 (46.7)			29.3 ± 4.8		0 (0)		NA	NA	NA		1 (1–4)	PSG	8
				OSA (60)	AHI ≥ 5	49.6 ± 11.7	29 (96.7)			31.6 ± 5.8		0 (0)		NA	NA	NA		24.5 (6–98)		
Vitarelli [80]	2016	Italy	67	Control subjects (30)	AHI < 5	46.2 ± 13.4	11 (36.7)			26.4 ± 4.3		0 (0)		0 (0)	0 (0)	NA		3.8 ± 1.4	PSG	8
				Mild OSA (10)	5 < AHI < 15	47.9 ± 10.3	4 (40)			26.9 ± 5.8		0 (0)		0 (0)	0 (0)	NA		7.1 ± 1.9		
				Moderate OSA (8)	15 < AHI < 30	47.6 ± 9.1	3 (37.5)			27.4 ± 5.5		0 (0)		0 (0)	0 (0)	NA		19.8 ± 2.7		
				Severe OSA (19)	AHI ≥ 30	48.1 ± 10.2	7 (36.8)			28.2 ± 6.3		0 (0)		0 (0)	0 (0)	NA		58.9 ± 9.1		
Vural [81]	2016	Turkey	63	Non-OSA (20)	AHI < 5	42.9 ± 13.1	12 (60)			26.2 ± 1.0		5 (25)		9 (45)	1 (5)	NA		2.7 ± 1.0	PSG	7
				Mild-to-moderate OSA (19)	5 ≤ AHI < 30	41.4 ± 12.6	12 (63.2)			26.4 ± 1.1		11 (57)		9 (47)	1 (5)	NA		14.9 ± 8.3		
				Severe OSA (24)	AHI ≥ 30	43.1 ± 11.1	13 (54.2)			26.8 ± 1.2		15 (62)		15 (62)	2 (8)	NA		60.9 ± 21.3		
Zhou [82]	2016	China	79	Controls (19)	AHI ≤ 5	52.0 ± 10.8	12 (63.2)			24.2 ± 3.7		NA		NA	NA	NA		NA	PSG	7
				Mild OSA (20)	5 < AHI ≤ 15	53.9 ± 11.7	16 (80)			30.6 ± 9.7		NA		NA	NA	NA				
				Moderate OSA (16)	15 < AHI ≤ 30	58.8 ± 10.7	13 (81.2)			35.0 ± 9.5		NA		NA	NA	NA				
				Severe OSA (24)	AHI > 30	49.7 ± 12.7	22 (91.7)			38.9 ± 9.5		NA		NA	NA	NA				
Buonauro [83]	2017	Italy	88	Control (29)	AHI ≤ 5	52.9 ± 10.5	23 (79.3)			26.3 ± 3.4		NA		NA	NA	NA		NA	Cardiorespiratory monitoring	6
				OSA (59)	AHI > 15	54.4 ± 11.2	49 (83.1)			33.2 ± 7.2		NA		NA	NA	NA		42.0 ± 24.3		
Vural [84]	2017	Turkey	162	Control (45)	AHI < 5	48.1 ± 11.8	24 (53.3)			26.7 ± 1.5		10 (44.4)		21 (46.7)	4 (8.9)	NA		2.8 ± 1.0	PSG	8
				Mild (22)	AHI 5–15	47.7 ± 11.8	14 (63.6)			27.2 ± 1.3		13 (59.1)		9 (40.9)	4 (18.2)	NA		9.3 ± 3.2		
				Moderate (27)	AHI 15–30	49.4 ± 12.1	13 (56.5)			27.2 ± 1.3		16 (69.6)		14 (60.9)	5 (21.7)	NA		24.9 ± 3.9		
				Severe (68)	AHI ≥ 30	49.9 ± 9.5	34 (59.6)			27.6 ± 2.3		31 (54.4)		26 (45.6)	9 (15.8)	NA		57.3 ± 20.4		
Zhou [85]	2017	China	82	Control (19)	AHI ≤ 5	52.0 ± 10.8	12 (63.2)			24.2 ± 3.7		0 (0)		NA	0 (0)	NA		NA	PSG	7
				Mild (21)	5 < AHI < 15	52.8 ± 11.9	15 (71.4)			29.6 ± 9.1		0 (0)		NA	0 (0)	NA				
				Moderate (19)	15 ≤ AHI < 30	55.7 ± 12.4	13 (68.4)			35.2 ± 10.1		0 (0)		NA	0 (0)	NA				
				Severe (23)	AHI ≥ 30	50.2 ± 12.1	18 (78.3)			39.2 ± 9.8		0 (0)		NA	0 (0)	NA				
Çetin [86]	2018	Turkey	55	Group I (26)	AHI 5–30	49.0 ± 10.8	17 (66)			26.9 ± 1.6		12 (46)		12 (46)	4 (15)	NA		8.9 ± 9.8	PSG	6
				Group II (29)	AHI ≥ 30	49.9 ± 9.4	15 (52)			27.2 ± 2.4		15 (51)		14 (48)	6 (20)	NA		61.1 ± 21.0		
Li [87]	2018	China	101	Control (30)	AHI ≤ 5	46.82 ± 5.45	17 (56.7)			27.06 ± 4.38		0 (0)		NA	0 (0)	NA		1.75 ± 0.99	PSG	7
				Mild OSA (23)	AHI 5–15	47.31 ± 6.15	13 (56.5)			28.40 ± 3.12		0 (0)		NA	0 (0)	NA		13.96 ± 3.98		
				Moderate OSA (25)	AHI 16–30	47.96 ± 7.90	14 (56)			29.83 ± 5.05		0 (0)		NA	0 (0)	NA		24.01 ± 3.56		
				Severe OSA (23)	AHI > 30	48.55 ± 5.43	14 (60.9)			32.97 ± 3.59		0 (0)		NA	0 (0)	NA		39.61 ± 6.64		
**CMR**																				
Javaheri [88]	2016	USA	1412	AHI < 5 (256)	AHI < 5	66.2 ± 8.7	69 (27.0)			26.1 ± 5.1		126 (49.2)		NA	27 (10.6)		NA	N/A	PSG	7
				AHI 5–15 (511)	AHI 5–15	67.4 ± 8.6	194 (38.0)			27.7 ± 4.7		270 (52.8)		NA	57 (11.2)		NA			
				AHI 15–30 (367)	AHI 15–30	69.0 ± 9.0	201 (54.8)			28.6 ± 4.8		204 (55.6)		NA	59 (16.1)		NA			
				AHI 30–50 (191)	AHI 30–50	69.2 ± 9.2	124 (64.9)			29.6 ± 5.6		114 (59.7)		NA	34 (17.9)		NA			
				AHI > 50 (87)	AHI > 50	68.5 ± 8.6	67 (77.0)			31.1 ± 5.2		55 (63.2)		NA	194 (13.8)		NA			
**SPECT**																				
Wang [89]	2013	China	63	Control (17)	AHI < 5	51.0 (42.0, 54.0)	13 (76.5)			28.6 (25.2, 31.3)		NA		NA	4 (23.5)	NA		3.3	PSG	8
				Mild (15)	AHI 5–20	48.0 (39.0, 55.0)	10 (66.7)			28.4 (25.7, 31.7)		NA		NA	3 (20.0)	NA		9.1		
				Moderate (13)	AHI 21–30	44.0 (39.0, 47.0)	9 (69.2)			28.5 (27.2, 32.1)		NA		NA	2 (15.4)	NA		38.5		
				Severe (18)	AHI ≥ 31	45.0 (36.0, 51.0)	17 (94.4)			30.2 (28.7, 33.3)		NA		NA	4 (22.2)	NA		65.2		

*AHI* apnea hypopnea index, *BMI* body mass index, *IVSD* interventricular septum diameter, *LAD* left atrial diameter, *LAVI* left atrial volume index, *LVEDD* left ventricular end-diastolic diameter, *LVESD* left ventricular end-systolic diameter, *LVM* left ventricular mass, *LVMI* left ventricular mass index, *LV MPI* left ventricular myocardial performance index, *LVEF* left ventricular ejection fraction, *NOS* Newcastle–Ottawa scale, *OSA* obstructive sleep apnea, *PSG* polysomnography, *PWD* posterior wall diameter, *SMD* standardized mean difference, *RVD* right ventricular diameter, *RV FAC* right ventricular fractional area change, *RVFWT* right ventricular free wall thickness, *TAPSE* tricuspid annular plane systolic excursion

We furtherly performed a meta-analysis for studies in cardiac structure and function which was assessed by echocardiography. According to the inclusion and exclusion criteria detailed earlier, 46 studies yielding 3082 OSA patients and 1774 controls were pooled for the meta-analysis. These pooled findings and subgroup analyses are summarized in Tables 4 and 5, respectively. Comprehensive details are shown in the Supplementary materials. Additionally, sensitivity analyses showed that no significant change occurred when an independent study was omitted, confirming that the results of our meta-analyses were stable.

**Table 4:** Results of the meta-analysis comparing OSA patients and controls

Echocardiographic parameters	Number of studies	OSA/control	SMD (95% CI)	*p* value	Study heterogeneity			Egger’s test *p* value
					***I***^**2**^	***χ***^**2**^	***p* value**	
LAD (mm)	13	1107/317	0.385 (0.252, 0.518)	< 0.001	39.1%	19.72	0.073	0.757
LAVI (ml/m^2^)	6	238/163	0.307 (0.096, 0.518)	0.004	89.50%	47.49	< 0.001	0.036
LVESD (mm)	24	1526/620	0.323 (0.223, 0.422)	< 0.001	0.00%	14.8	0.902	0.646
LVEDD (mm)	18	918/406	0.126 (0.003, 0.249)	0.044	0.00%	13.98	0.669	0.638
LVM (g)	7	708/612	0.558 (0.403, 0.712)	< 0.001	87.3%	47.42	< 0.001	0.807
LVMI (g/m^2^)	23	1515/641	0.478 (0.242, 0.714)	< 0.001	81.3%	117.7	< 0.001	0.562
IVSD (mm)	24	1375/808	0.471 (0.195, 0.747)	0.001	87.1%	177.71	< 0.001	0.021
PWD (mm)	22	1348/1047	0.602 (0.328, 0.875)	< 0.001	86.7%	157.47	< 0.001	0.087
LVEF (%)	39	2552/1737	− 0.238 (− 0.379, − 0.097)	0.001	73.4%	142.76	< 0.001	0.465
LV MPI	8	512/385	0.687 (0.371, 1.004)	< 0.001	74.4%	27.38	< 0.001	0.915
RVD (mm)	15	845/470	0.725 (0.605, 0.845)	< 0.001	82.7%	80.8	< 0.001	0.184
RV MPI	8	303/346	0.881 (0.487, 1.274)	< 0.001	79.4%	33.98	< 0.001	0.052
TAPSE	10	435/419	− 0.481 (− 0.810, − 0.152)	0.004	79.0%	42.9	< 0.001	0.12
RV FAC	5	762/234	− 0.399 (− 0.553, − 0.246)	< 0.001	0.00%	2.59	0.629	0.222

*IVSD* interventricular septum diameter, *LAD* left atrial diameter, *LAVI* left atrium volume index, *LVEDD* left ventricular end-diastolic diameter, *LVESD* left ventricular end-systolic diameter, *LVM* left ventricular mass, *LVMI* left ventricular mass index, *LV MPI* left ventricular myocardial performance index, *LVEF* left ventricular ejection fraction, *OSA* obstructive sleep apnea, *PWD* posterior wall diameter, *SMD* standardized mean difference, *RVD* right ventricular diameter, *RV FAC* right ventricular fractional area change, *RV MPI* right ventricular myocardial performance index, *TAPSE* tricuspid annular plane systolic excursion

**Table 5:** Subgroup analysis

Echocardiographic parameters	Subgroup	*N*	SMD (95% CI)	*p* value	Study heterogeneity		
					**I**^**2**^	**χ**^**2**^	***p* value**
**LAVI**	**Overall**	**6**	**0.307 (0.096, 0.518)**	**0.004**	**89.50%**	**47.49**	** < 0.001**
	**Age**						
	Age ≥ 50	2	0.598 (− 0.070, 1.265)	0.886	9.6%	10.17	0.293
	Age < 50	4	0.962 (− 0.054, 1.978)	0.064	92.7%	41.05	< 0.001
	**BMI**						
	BMI ≥ 30	3	1.121 (− 0.585, 2.828)	0.198	95.6%	45.02	< 0.001
	BMI < 30	3	0.262 (− 0.026, 0.550)	0.075	11.0%	2.25	0.325
**LVM**	**Overall**	**7**	**0.558 (0.403, 0.712)**	** < 0.001**	**87.3%**	**47.42**	** < 0.001**
	**Age**						
	Age ≥ 50	5	0.648 (− 0.007, 1.371)	0.079	90.5%	42.10	< 0.001
	Age < 50	2	0.593 (0.121, 1.066)	0.001	0.0%	0.60	0.438
	**BMI**						
	BMI ≥ 30	2	0.593 (0.121, 1.066)	0.224	94.9%	19.65	< 0.001
	BMI < 30	5	0.382 (0.121, 0.644)	0.004	37.9%	6.44	0.168
**LVMI**	**Overall**	**23**	**0.478 (0.242, 0.714)**	** < 0.001**	**81.3%**	**117.7**	** < 0.001**
	**Age**						
	Age ≥ 50	11	0.332 (− 0.047, 0.712)	0.086	85.7%	69.94	< 0.001
	Age < 50	12	0.619 (0.242, 0.714)	< 0.001	71.1%	38.06	< 0.001
	**BMI**						
	BMI ≥ 30	7	0.211 (− 0.227, 0.650)	0.345	83.0%	35.29	< 0.001
	BMI < 30	16	0.598 (0.311, 0.884)	< 0.001	81.2%	79.94	< 0.001
**IVSD**	**Overall**	**24**	**0.471 (0.195, 0.747)**	**0.001**	**87.1%**	**177.71**	** < 0.001**
	**Age**						
	Age ≥ 50	10	0.569 (− 0.007, 1.145)	0.053	93.6%	141.06	< 0.001
	Age < 50	14	0.396 (0.166, 0.626)	0.001	64.5%	36.60	< 0.001
	**BMI**						
	BMI ≥ 30	10	0.471 (0.195, 0.747)	0.005	83.0%	52.84	< 0.001
	BMI < 30	14	0.446 (0.046, 0.846)	0.029	88.4%	112.43	< 0.001
**PWD**	**Overall**	**22**	**0.602 (0.328, 0.875)**	** < 0.001**	**86.7%**	**157.47**	** < 0.001**
	**Age**						
	Age ≥ 50	9	0.712 (0.084, 1.340)	0.026	93.7%	127.17	< 0.001
	Age < 50	13	0.511 (0.304, 0.718)	< 0.001	58.8%	29.11	0.004
	**BMI**						
	BMI ≥ 30	8	0.356 (− 0.276, 0.988)	0.270	92.9%	98.19	< 0.001
	BMI < 30	14	0.730 (0.504, 0.955)	< 0.001	68.0%	40.68	< 0.001
**LVEF**	**Overall**	**39**	− **0.238 (**− **0.379,** − **0.097)**	**0.001**	**73.4%**	**142.76**	** < 0.001**
	**Age**						
	Age ≥ 50	16	− 0.142 (− 0.378, 0.094)	0.238	80.5%	76.92	< 0.001
	Age < 50	23	− 0.310 (− 0.476, − 0.143)	< 0.001	62.8%	59.07	< 0.001
	**BMI**						
	BMI ≥ 30	13	− 0.254 (− 0.630, 0.121)	0.184	87.5%	95.72	0.005
	BMI < 30	26	− 0.239 (− 0.358, − 0.120)	< 0.001	46.4%	46.63	< 0.001
**LV MPI**	**Overall**	**8**	**0.687 (0.371, 1.004)**	** < 0.001**	**74.4%**	**27.38**	** < 0.001**
	**Age**						
	Age ≥ 50	2	0.456 (− 0.413, 1.326)	0.304	92.4%	13.09	< 0.001
	Age < 50	6	0.773 (0.426, 1.121)	< 0.001	63.0%	13.50	0.019
	**BMI**						
	BMI ≥ 30	4	0.663 (0.091, 1.235)	0.023	80.9%	15.72	0.001
	BMI < 30	4	0.734 (0.363, 1.105)	< 0.001	66.7%	9.02	0.029
**RVD**	**Overall**	**15**	**0.725 (0.605, 0.845)**	** < 0.001**	**82.7%**	**80.8**	** < 0.001**
	**Age**						
	Age ≥ 50	11	0.678 (0.318, 1.039)	< 0.001	70.2%	10.06	0.018
	Age < 50	4	0.873 (0.454, 1.293)	< 0.001	85.5%	69.08	< 0.001
	**BMI**						
	BMI ≥ 30	8	0.922 (0.442, 1.403)	< 0.001	87.0%	53.65	< 0.001
	BMI < 30	7	0.676 (0.329, 1.023)	< 0.001	72.6%	21.93	0.001
**RV MPI**	**Overall**	**8**	**0.881 (0.487, 1.274)**	** < 0.001**	**79.4%**	**33.98**	** < 0.001**
	**Age**						
	Age ≥ 50	3	0.723 (− 0.151, 1.597)	0.105	89.9%	19.70	< 0.001
	Age < 50	5	0.973 (0.586, 1.359)	< 0.001	62.8%	10.75	0.029
	**BMI**						
	BMI ≥ 30	5	0.651 (0.161, 1.141)	0.009	80.1%	20.07	< 0.001
	BMI < 30	3	1.226 (0.879, 1.572)	< 0.001	20.5%	2.52	0.284
**TAPSE**	**Overall**	**10**	− **0.481 (**− **0.810,** − **0.152)**	**0.004**	**79.0%**	**42.9**	** < 0.001**
	**Age**						
	Age ≥ 50	4	− 0.476 (− 1.137, 0.185)	0.158	74.2%	23.27	< 0.001
	Age < 50	6	− 0.488 (− 0.874, − 0.102)	0.013	87.1%	19.38	0.002
	**BMI**						
	BMI ≥ 30	5	− 0.357 (− 0.896, 0.182)	0.194	85.0%	26.74	< 0.001
	BMI < 30	5	− 0.610 (− 0.987, − 0.232)	0.002	66.2%	11.84	0.019

*IVSD* interventricular septum diameter, *LAD* left atrial diameter, *LAVI* left atrium volume index, *LVM* left ventricular mass, *LVMI* left ventricular mass index, *LV MPI* left ventricular myocardial performance index, *LVEF* left ventricular ejection fraction, *OSA* obstructive sleep apnea, *PWD* posterior wall diameter, *SMD* standardized mean difference, *RVD* right ventricular diameter, *RV FAC* right ventricular fractional area change, *RV MPI* right ventricular myocardial performance index, *TAPSE* tricuspid annular plane systolic excursion

### OSA and left cardiac structure and function

The parameters of left atrial diameter (LAD) and left atrium volume index (LAVI) were used to assess LA remodeling. The relationship between OSA and LAD was reported in 13 studies involving 1107 OSA patients and 317 controls. This meta-analysis revealed that compared to controls, OSA patients displayed significantly wider LAD (SMD [95% CI] 0.385 [0.252, 0.518]; *p* < 0.001), with non-significant heterogeneity. No significant publication bias (*p* = 0.757) was found among the studies. Differences in LAVI were reported in 6 studies involving 238 OSA patients and 163 controls. This meta-analysis found that LAVI in OSA patients was significantly higher than that in controls (SMD [95% CI] 0.307 [0.096, 0.518]; *p* = 0.004), with statistically significant heterogeneity. The subgroup analysis indicated that heterogeneity in OSA patients with age ≥ 50 years or BMI < 30 kg/m^2^ was significantly decreased. In this analysis, there was publication bias on Egger test (*p* = 0.036).

LV remodeling was assessed based on left ventricular (LV) end-systolic diameter (LVESD), LV end-diastolic diameter (LVEDD), LV mass (LVM), LV mass index (LVMI), interventricular septum diameter (IVSD), and posterior wall diameter (PWD). Differences in LVESD were reported in 24 studies involving 1526 OSA patients and 620 controls. This meta-analysis found that compared to controls, the OSA patients had significantly wider LVESD (SMD [95% CI] 0.323 [0.223, 0.422]; *p* < 0.001), with non-significant heterogeneity. No significant publication bias (*p* = 0.646) was found among the studies. LVEDD was assessed in 18 studies involving 918 OSA patients and 406 controls. This meta-analysis found that compared to controls, OSA patients displayed significantly wider LVEDD (SMD [95% CI] 0.126 [0.003, 0.249]; *p* = 0.669), with non-significant heterogeneity. No significant publication bias was found among the studies (*p* = 0.638). Differences in LVM were reported in 7 studies involving 708 OSA patients and 612 controls. Our analysis revealed that compared to controls, LVM was substantially higher in OSA patients (SMD [95% CI] 0.558 [0.403, 0.712]; *p* < 0.001), with statistically significant heterogeneity. Further subgroup analysis showed that heterogeneity in OSA patients with age < 50 years or BMI < 30 kg/m^2^ was significantly decreased. No significant publication bias was found among the studies (*p* = 0.807). Differences in LVMI were reported in 23 studies involving 1515 OSA patients and 641 controls. Compared to controls, OSA patients exhibited significantly higher LVMI (SMD [95% CI] 0.478 [0.242, 0.714]; *p* < 0.001), with statistically significant heterogeneity. No significant publication bias was found among the studies (*p* = 0.562). Differences in IVSD were reported in 24 studies involving 1375 OSA patients and 808 controls. We found that compared with controls, OSA patients have significantly wider IVSD (SMD [95% CIs] 0.471 [0.195, 0.747]; *p* = 0.001), with statistically significant heterogeneity. And Egger test revealed a significant publication bias among the studies (*p* = 0.021). Differences in PWD were reported in 22 studies involving 1348 OSA patients and 1047 controls. The meta-analysis showed that OSA patients exhibited significantly wider PWD than that in controls (SMD [95% CIs] 0.602 [0.328, 0.875]; *p* < 0.001), with statistically significant heterogeneity. No significant publication bias was found among the studies (*p* = 0.087). There was no apparent change in the heterogeneity of LVMI, IVSD, and PWD when the subgroups were performed using the age of 50 years or the BMI of 30 kg/m^2^ as the threshold.

LV dysfunction was diagnosed based on LV ejection fraction (LVEF) and LV myocardial performance index (LV MPI). Differences in LVEF were reported in 39 studies involving 2552 OSA patients and 1737 controls. This meta-analysis found that compared with controls, LVEF was significantly lower in OSA patients (SMD [95% CI] − 0.238 [− 0.379, − 0.097]; *p* = 0.001), with statistically significant heterogeneity. There was non-significant publication bias (*p* = 0.465). Differences in LV MPI were reported in 8 studies involving 512 OSA patients and 385 controls. This meta-analysis found that compared to controls, OSA patients displayed significantly higher LV MPI (SMD [95% CI] 0.687 [0.371, 1.004]; *p* < 0.001) with statistically significant heterogeneity. Non-significant publication bias was found among these studies (*p* = 0.915). Additionally, there was no apparent change in the heterogeneity of LVEF and LV MPI when subgroup analyses were performed using the age of 50 years or the BMI of 30 kg/m^2^ as the threshold.

### OSA and right cardiac structure and function

Right atrial diameter (RAD) was used in three studies to assess the RA remodeling. Of these, only Sun et al. [68] found that RAD in OSA group was significantly higher than that in the control group.

RV remodeling was evaluated based on the right ventricular (RV) diameter (RVD) and RV free-wall thickness (RV FWT). Differences in RVD were reported in 15 studies involving 845 OSA patients and 470 controls. The meta-analysis found that compared to controls, OSA patients exhibited significantly higher RVD (SMD [95% CI] 0.725 [0.605, 0.845]; *p* < 0.001), with statistically significant heterogeneity, while no apparent change in the heterogeneity when the subgroups were performed using the age of 50 years or the BMI of 30 kg/m^2^ as the threshold. No significant publication bias was detected among the studies (*p* = 0.184). Three studies used RV FWT to assess right ventricular remodeling. However, only one study found that there was significant difference in terms of RV FWT between severe OSA patients and controls [80].

Assessment of RV dysfunction was based on RV MPI, tricuspid annular plane systolic excursion (TAPSE), and RV fractional area change (RV FAC). Differences in RV MPI were reported in 8 studies involving 303 OSA patients and 346 controls. This meta-analysis found that OSA patients displayed significantly higher RV MPI than the controls (SMD [95% CI] 0.881 [0.487, 1.274]; *p* < 0.001), with statistically significant heterogeneity. Subgroup analysis showed that heterogeneity in OSA patients with BMI < 30 kg/m^2^ was significantly decreased. No significant publication bias was found among the studies (*p* = 0.052). Differences in TAPSE were reported in 10 studies involving 435 OSA patients and 419 controls. Herein, TAPSE was found to be significantly lower in OSA patients than controls (SMD [95% CI] − 0.481 [− 0.810, − 0.152]; *p* = 0.004), with statistically significant heterogeneity. There was no apparent change in the heterogeneity when the subgroups were performed using the age of 50 years or the BMI of 30 kg/m^2^ as the threshold. No significant publication bias was found among the studies (*p* = 0.12). Differences in RV FAC were reported in 5 studies involving 294 OSA patients and 158 controls. In this meta-analysis, we found that compared to controls, OSA patients displayed a significantly lower RV FAC (SMD [95% CI] − 0.399 [− 0.553, − 0.246]; *p* < 0.001), with non-significant heterogeneity. No significant publication bias was found among the studies (*p* = 0.222).

### Obstructive sleep apnea and myocardial injury

In a community-based cohort, Shah et al. [90] found that LV scar measured using CMR with LGE was more prevalent in patients with OSA than those without OSA. After multivariable adjustment, OSA is still associated with over a two-fold increase in the odds of LV scar presence, a majority of which were atypical and clinically unrecognized. In another study, Okuda et al. [91] demonstrated that moderate to severe OSA patients had less myocardial contractile reserve than those with less severe OSA. In addition, the severity of hypoxic events during sleep was also independently associated with myocardial contractile reserve.

## Discussion

### Main finding

Overall, this systematic review and meta-analysis summarized findings on the association between OSA and cardiovascular abnormalities diagnosed using imaging techniques in 82 studies published in the last 15 years. The major findings were as follows: (1) OSA increases the risk of developing coronary atherosclerosis. (2) There were significant alterations in patients with OSA regarding the parameters of cardiac remodeling and dysfunction, which illustrated that atrial enlargement, ventricular hypertrophy, and cardiac dysfunction were more common among OSA patients. (3) OSA is associated with subclinical myocardial injury.

### OSA and coronary artery calcification score

Numerous studies included in this review reported OSA was associated with CAC score. However, in some instances, the association was no longer significant after adjustment for traditional risk factors such as BMI. While in the real-world study, several of these risk factors were shared by patients with OSA and coronary atherosclerosis, which illustrates the complexity of the interaction between OSA and CAC score. In addition, the same CAC score may represent two different types of lesions. One type is spotty calcification, which is a mixed plaque with small punctate calcification, and another one is pure small calcified plaque lesion [92]. However, the risk degree of the two lesions is markedly different. Histological investigations demonstrated that spotty calcification appears frequently in unstable plaques [92]. However, there is no relevant research on OSA and spotty calcifications. One possible explanation is that spotty calcification is a relatively subjective assessment index. Moreover, the low resolution of smooth convolution kernel, the conventional image reconstruction mode in coronary CTA, affects the measurement of diameters for spotty calcified lesions [93].

### OSA and coronary plaque burden

All included studies demonstrated that OSA increases the risk of developing coronary plaque burden. Qualitative analysis showed that non-calcified and mixed plaque was more likely to present in OSA patients. Quantitative analysis showed that plaque volume was higher in patients with OSA than those without. However, there were inconsistent findings on the features of vulnerable plaque. Kent et al. [28] found no significant difference in the number of soft plaques between low-AHI group and high-AHI group, while Hamaoka et al. [8] recently reported that coronary low-attenuation plaque volume was associated with AHI. The potential reason may be the low-attenuation plaque volume not only existed in soft plaque, but existed in partially calcified plaque. In addition, the latter study used 320-slice CT to quantitatively evaluate coronary plaque burden and PSG to evaluate the severity of OSA, as opposed to the 64-slice CT and polygraphy used in the former. The more advanced technologies used in the latter study may have revealed more reliable findings. Of the invasive studies which assessed the association between OSA and plaque vulnerability, only one study [24], of 93 patients pre-treated with statin before recruitment, found no significant differences regarding the tissue composition of plaques and prevalence of thin cap fibroatheroma between OSA patients and controls. It was believed that the composition of tissue plaques and the incidence of thin cap fibroatheroma may have been influenced by statins [24, 94].

The linking of OSA to increased coronary plaque burden has been established. OSA and related intermittent hypoxia induces endothelial dysfunction mainly by activating numerous inflammatory responses, which will further accelerate coronary atherosclerosis. Indeed, OSA patients display increased secretion of circulating proinflammatory cytokines, chemokines, and adhesion molecules [95–97]. Our recent meta-analysis revealed that adiponectin levels, which are an anti-inflammatory and anti-atherosclerosis molecule, were significantly lower in patients with OSA than that in controls [98]. And continuous positive airway pressure can effectively reverse inflammatory cytokine levels [96]. However, there is no evidence on whether continuous positive airway pressure can ameliorate coronary plaque formation. Therefore, the future perspective in this domain is to elucidate the effect of OSA treatment on coronary plaques.

### OSA and cardiac remodeling and dysfunction

This meta-analysis summarized the relationship between OSA and cardiac structure and function, as assessed by echocardiography. We found OSA patients exhibit significant alterations in several parameters of cardiac structure (wider LAD, LVESD, LVEDD, LVM IVSD, and PWD RVD and higher LAVI and LVMI) and function (increase in LV MPI and RV MPI and decrease in LVEF, TAPSE, and RV FAC). In addition, one CMR study also showed that individuals with severe OSA were more likely to have lower LVEF, and higher AHI levels are strongly associated with higher LVM. Even though there were publication biases regarding LAVI and IVSD findings, trim-and-fill test revealed the bias did not impact on the estimates of LAVI (i.e., no trimming performed because data was unchanged). Additionally, in some instance, the heterogeneity in OSA patients was significantly decreased when the subgroups were performed using the age of 50 years or the BMI of 30 kg/m^2^ as the threshold, suggesting that age and BMI may be potential sources of heterogeneity.

Several mechanisms other than intermittent hypoxia might also be implicated for cardiac remodeling and dysfunction in patients with OSA. For instance, the negative intrathoracic pressure increases transmural pressure of the atria, ventricles, and aorta. Meanwhile, arousals during apneic and hypopnea episodes activate sympathetic pathway and increase the blood pressure. Over time, these events increase the LV and RV afterload and induce ventricular hypertrophy, cardiac diastolic and systolic dysfunction, and heart failure [4].

### OSA and myocardial injury

OSA-related myocardial injury is increasingly well understood. In a rat model, Chen et al. found that IH-induced increased the expression of myocardial transforming growth factor-beta and oxidative stress concomitant with lower levels of tissue-inhibitor of metalloproteinase-1 and higher levels of collagen-1 mRNA and fibronectin mRNA. Taken together, these findings suggest that IH promotes cardiac fibrosis [99]. Our recent review also concluded that IH appears to have a direct effect on myocardial extracellular matrix [100]. In current review, two studies evaluated myocardial injury in patients with OSA using myocardial contractile reserve and LV scar, respectively. Both studies revealed that OSA is associated with subclinical myocardial injury. However, none of the studies quantified the impact of OSA on myocardial tissue damage. T1-mapping CMR is an emerging quantitative technique for the assessment of myocardial injury [101]. Native (non-contrast) T1 values reflect several alterations in myocardial tissue composition, such as edema, necrosis, and fibrosis [102–104]. Combining native and post contrast T1 assessment allows for quantification of the extracellular volume fraction, which provides further information about intercellular matrix, extracellular matrix remodeling, and extent of myocardial injury [105].

### Study limitations

Firstly, web of science, google scholar, and Scopus databases were not searched, so we cannot claim to have been exhaustive in retrieving all studies. Secondly, no subgroup analysis of OSA stratification was performed because of the limited number of studies with AHI < 15 or AHI > 30 events/h. Finally, all included studies were cross-sectional studies and thus a causal relationship cannot be determined.

## Conclusions

OSA patients have an increased risk of developing coronary atherosclerosis and subclinical myocardial injury. In addition, OSA patients are more likely to have cardiac remodeling and dysfunction, such as atrial enlargement, ventricular hypertrophy, and cardiac dysfunction, than controls.

## Supplementary Information

**Additional file 1: Table S1** A example of search strategies. **Table S2** Equations. **Table S3** Certainty of evidence. **Figure S1** Forest plot. **Figure S2** Funnel plot. **Figure S3** Sensitivity plot.

## Data Availability

Please contact author for data requests.

## Abbreviations

AHI: Apnea hypopnea index
BMI: Body mass index
CAC: Coronary artery calcification
CAD: Coronary artery disease
CMR: Cardiac magnetic resonance
CT: Computed tomography
CTA: Computed tomography angiography
IH: Intermittent hypoxia
IVSD: Interventricular septum diameter
LAD: Left atrial diameter
LAVI: Left atrium volume index
LVEDD: Left ventricular end-diastolic diameter
LVESD: Left ventricular end-systolic diameter
LVM: Left ventricular mass
LVMI: Left ventricular mass index
LV MPI: Left ventricular myocardial performance index
LVEF: Left ventricular ejection fraction
NOS: Newcastle–Ottawa scale
OSA: Obstructive sleep apnea
PWD: Posterior wall diameter
PSG: Polysomnography
SMD: Standardized mean difference
RVD: Right ventricular diameter
RV FAC: Right ventricular fractional area change
RV FWT: Right ventricular free wall thickness
TAPSE: Tricuspid annular plane systolic excursion
