# Supplementary material for: Obstructive sleep apnea increases the risk of cardiovascular damage: a systematic review and meta-analysis of imaging studies
Source: Syst Rev. 2021 Jul 30;10:212. doi: 10.1186/s13643-021-01759-6 (PMC8325188; doi:10.1186/s13643-021-01759-6)
Supplement: Supplementary file 1 — Additional file 1: Table S1 A example of search strategies. Table S2 Equations. Table S3 Certainty of evidence. Figure S1 Forest plot. Figure S2 Funnel plot. Figure S3 Sensitivity plot. [file 13643_2021_1759_MOESM1_ESM.zip › Figure S1 Forest plotsR1.docx]

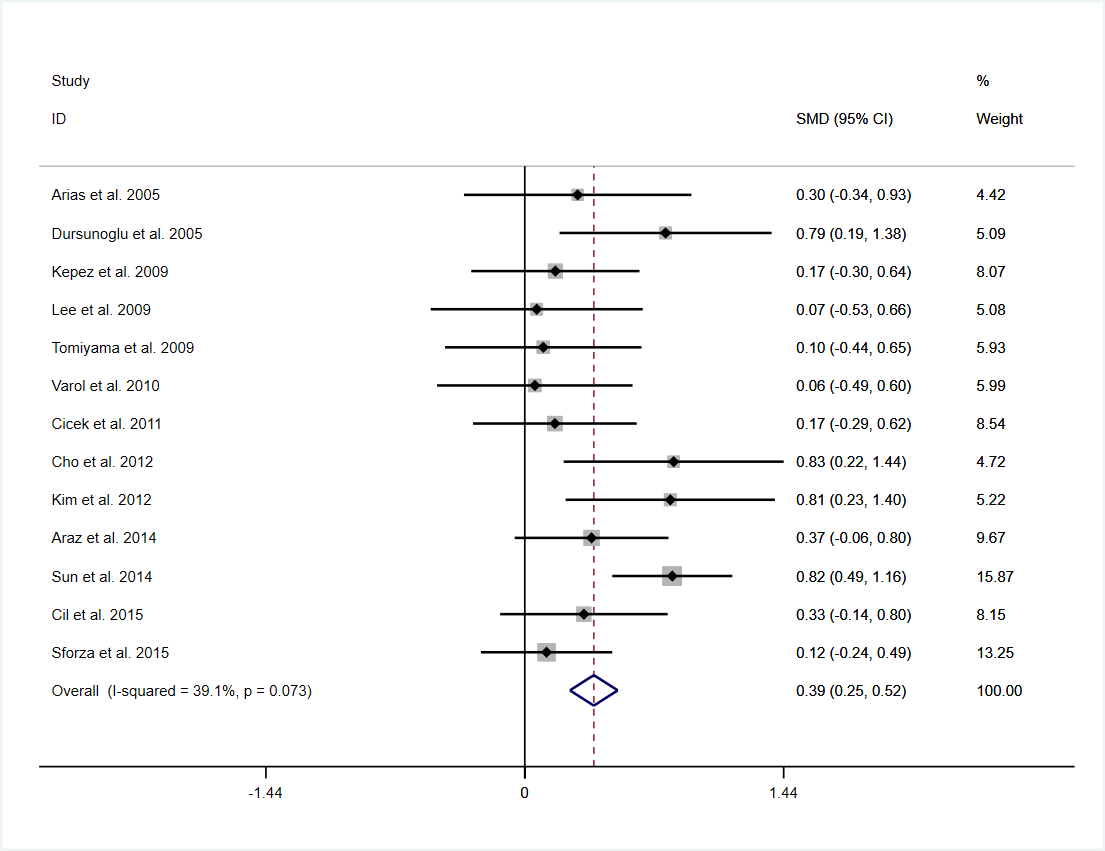


1. **Forest plot of the differences in left atrial diameter between the OSA patients and controls based on echocardiography**

**
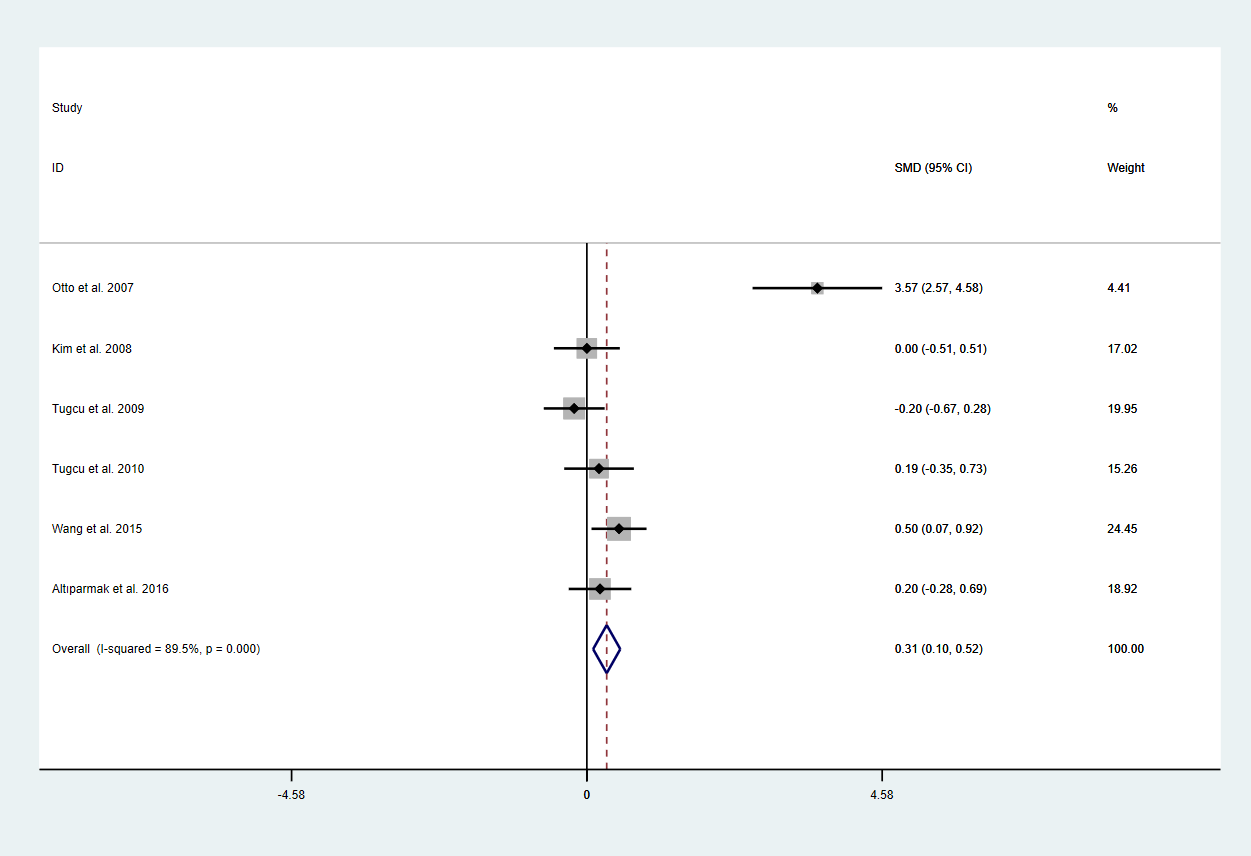
**

1. **Forest plot of the differences in left atrium volume index between the OSA patients and controls based on echocardiography**


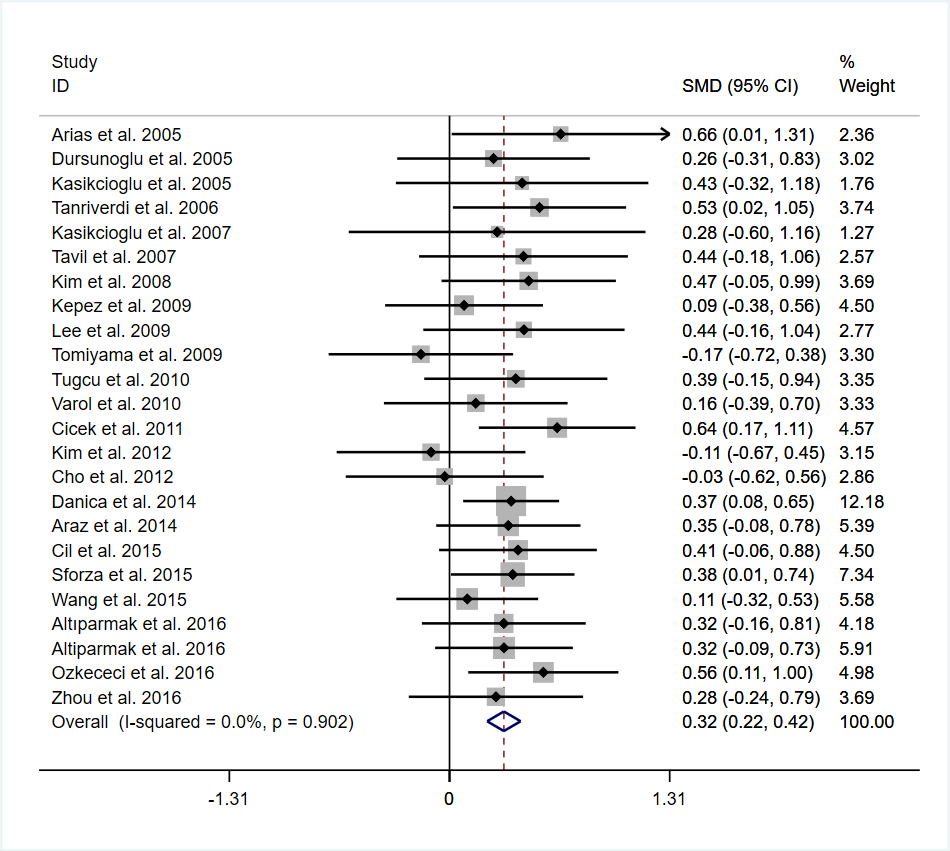


1. **Forest plot of the differences in left ventricular end-systolic diameter between the OSA patients and controls based on echocardiography**


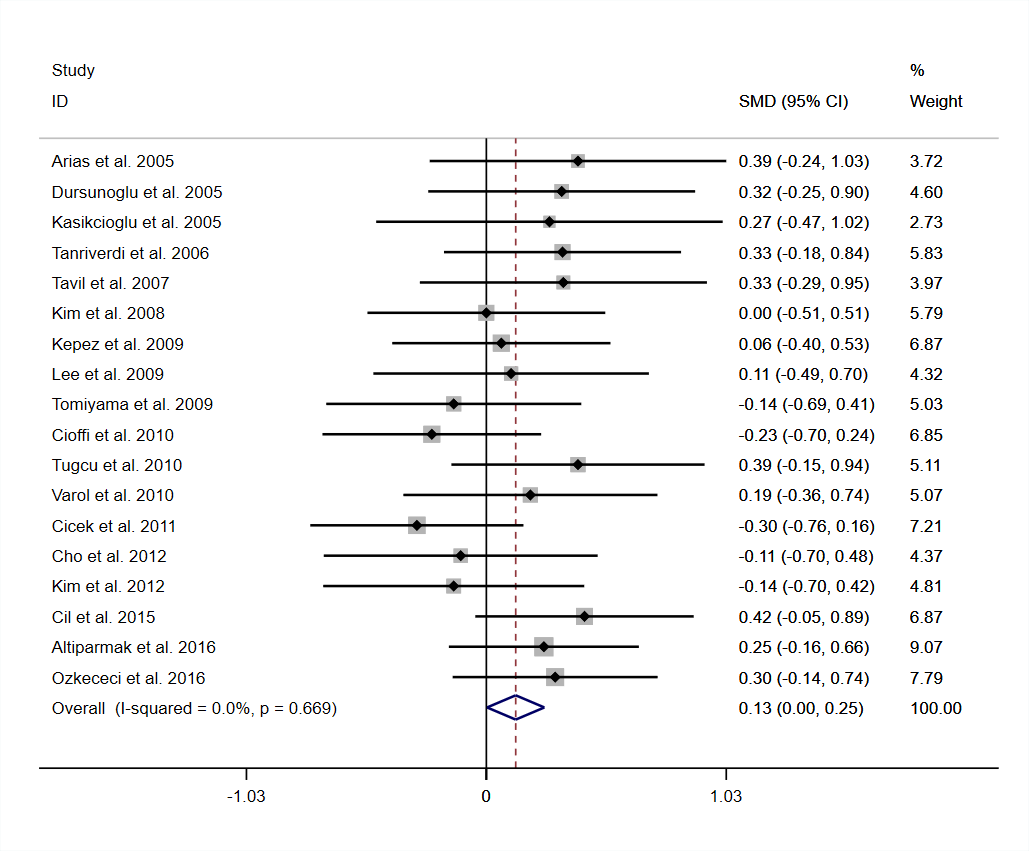


**d. Forest plot of the differences in left ventricular end-diastolic diameter between the OSA patients and controls based on echocardiography**


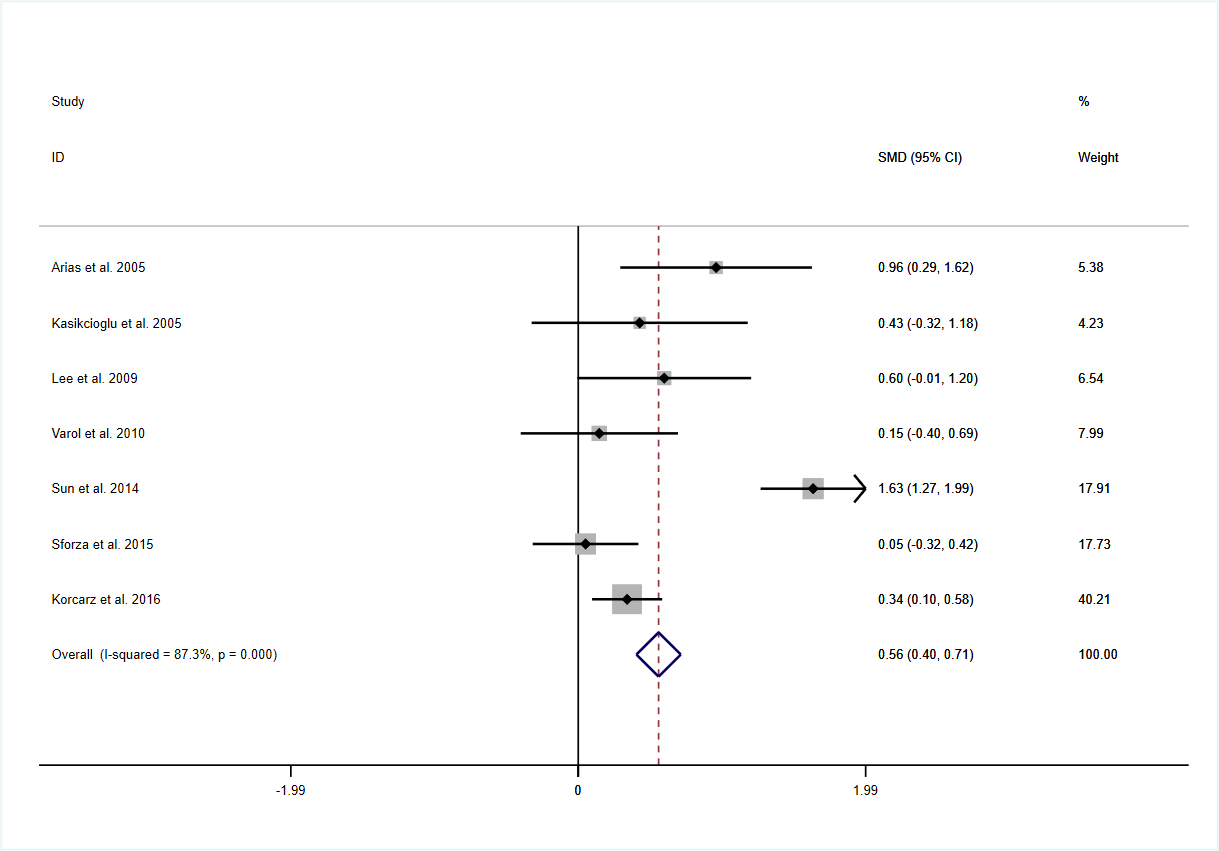


1. **Forest plot of the differences in left ventricular mass between the OSA patients and controls based on echocardiography**


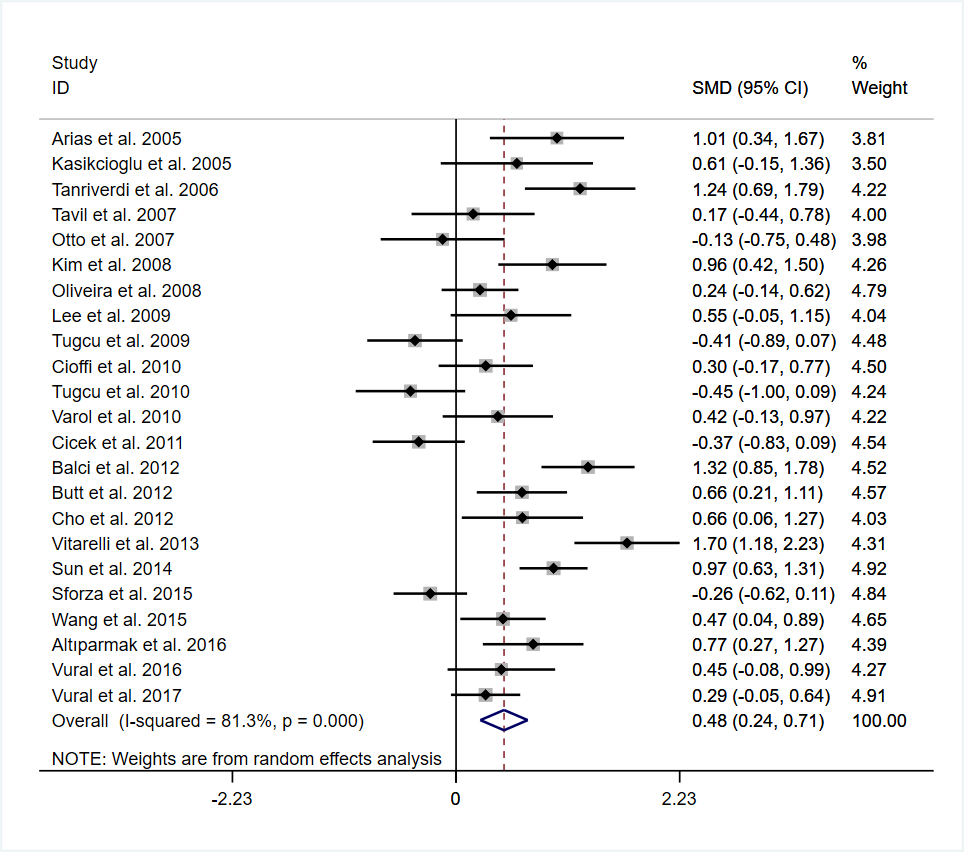


1. **Forest plot of the differences in left ventricular mass index between the OSA patients and controls based on echocardiography**

**
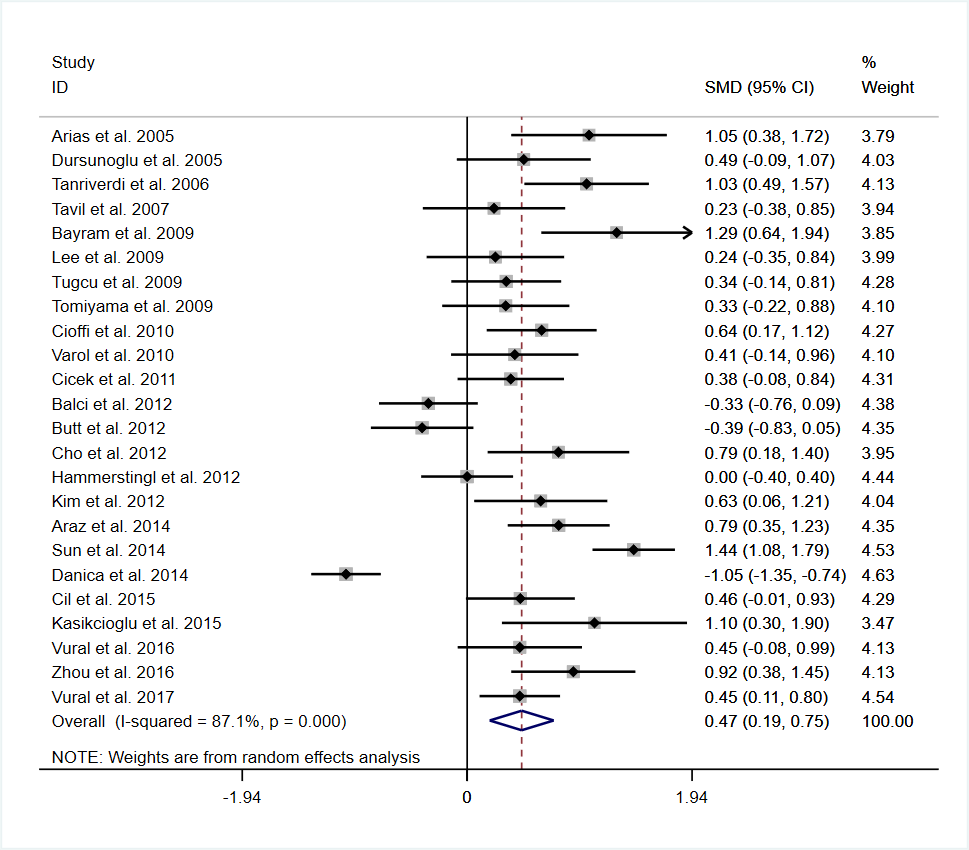
**

1. **Forest plot of the differences in interventricular septum diameter between the OSA patients and controls based on echocardiography**


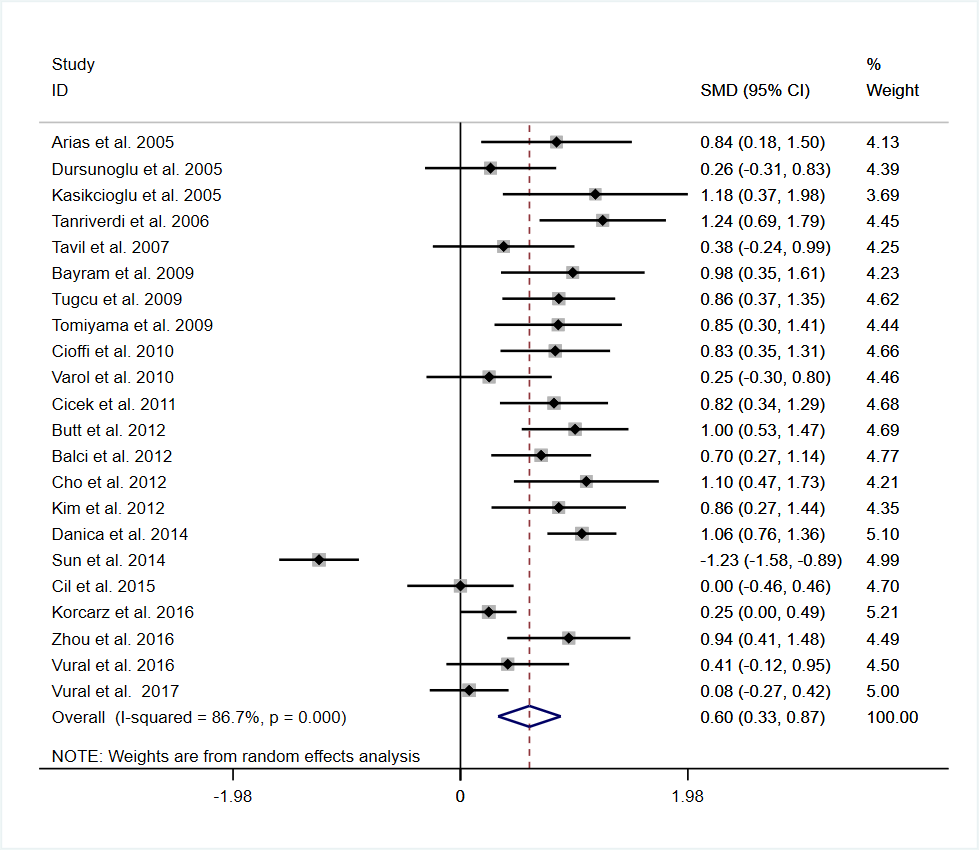


1. **Forest plot of the differences in posterior wall diameter between the OSA patients and controls based on echocardiography
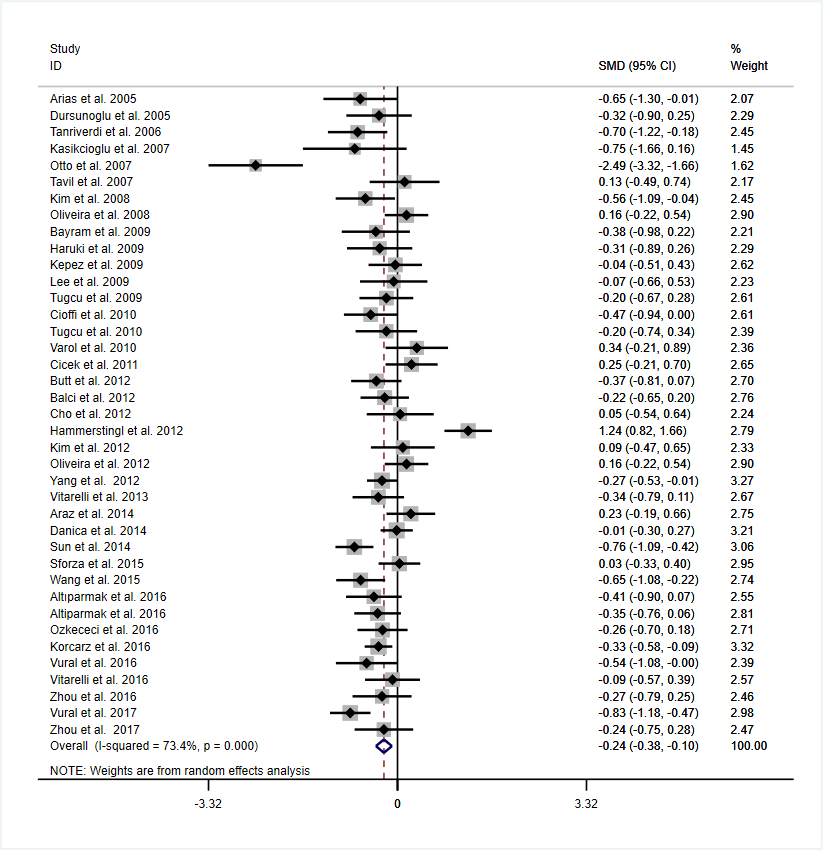
**
2. **Forest plot of the differences in left ventricular ejection fraction between the OSA patients and controls based on echocardiography**
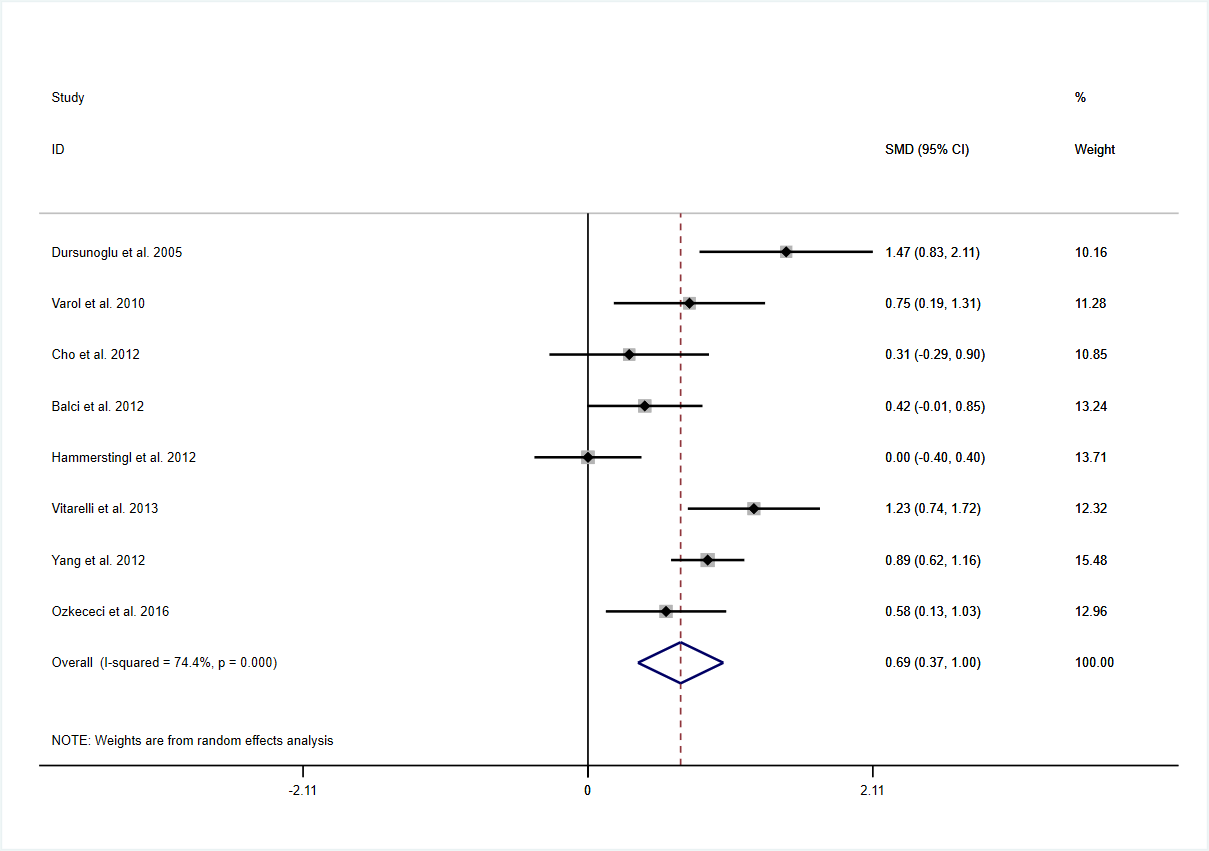

3. **Forest plot of the differences in left ventricular myocardial performance index between the OSA patients and controls based on echocardiography**
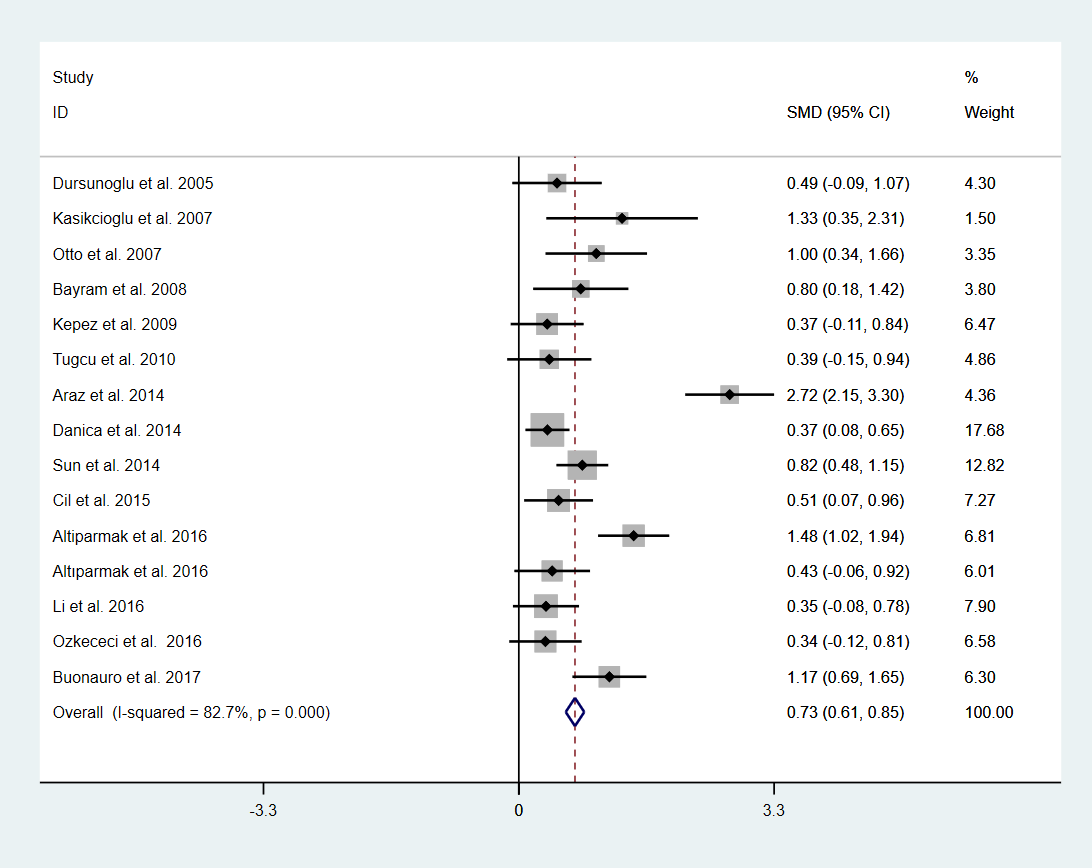

4. **Forest plot of the differences in right ventricular diameter between the OSA patients and controls based on echocardiography**


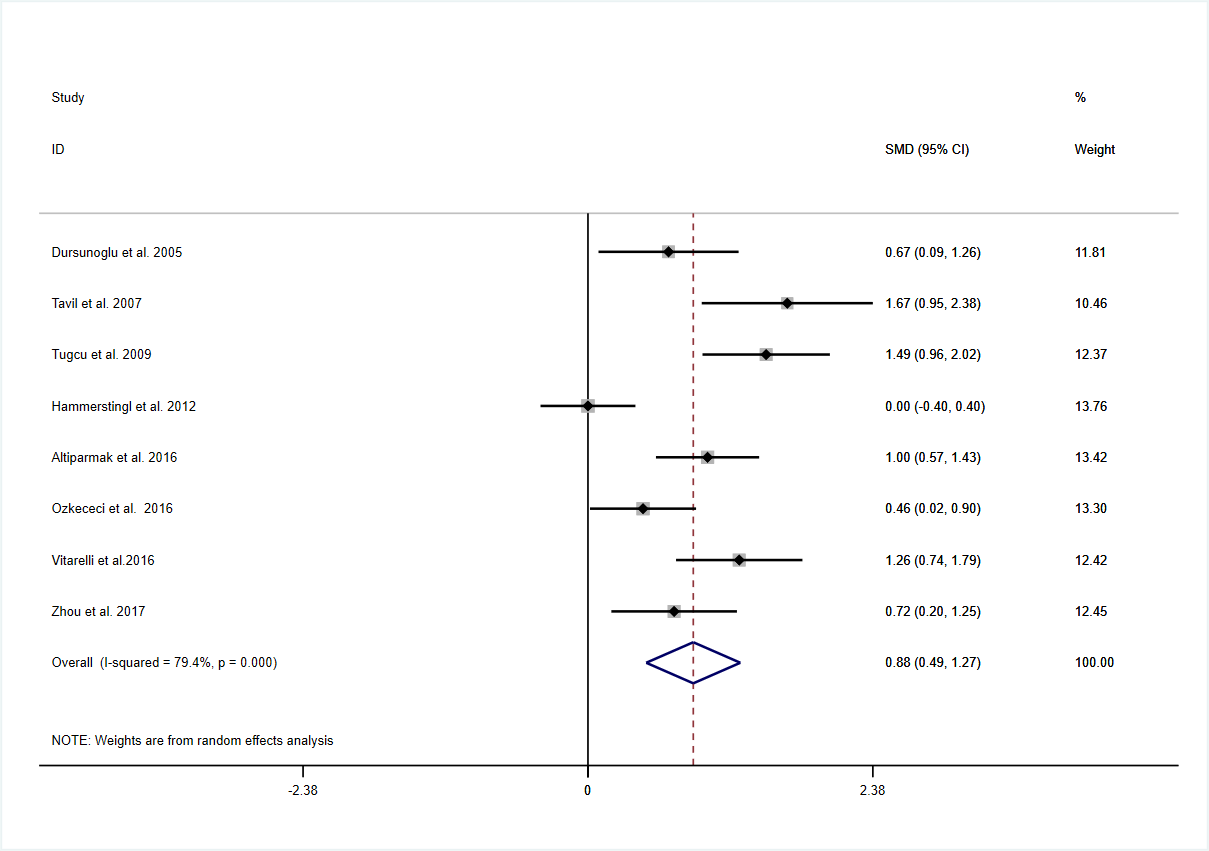


1. **Forest plot of the differences in right ventricular myocardial performance index between the OSA patients and controls based on echocardiography**


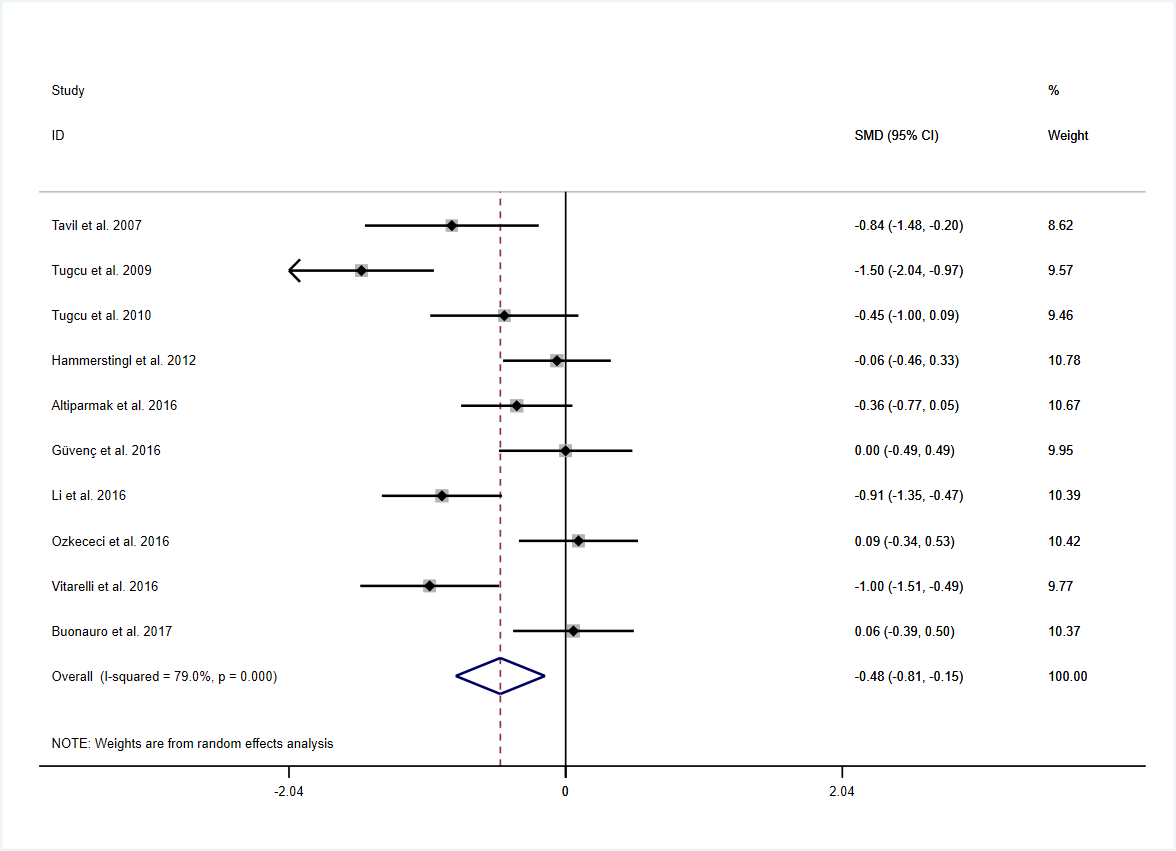


1. **Forest plot of the differences in tricuspid annular plane systolic excursion between the OSA patients and controls based on echocardiography**


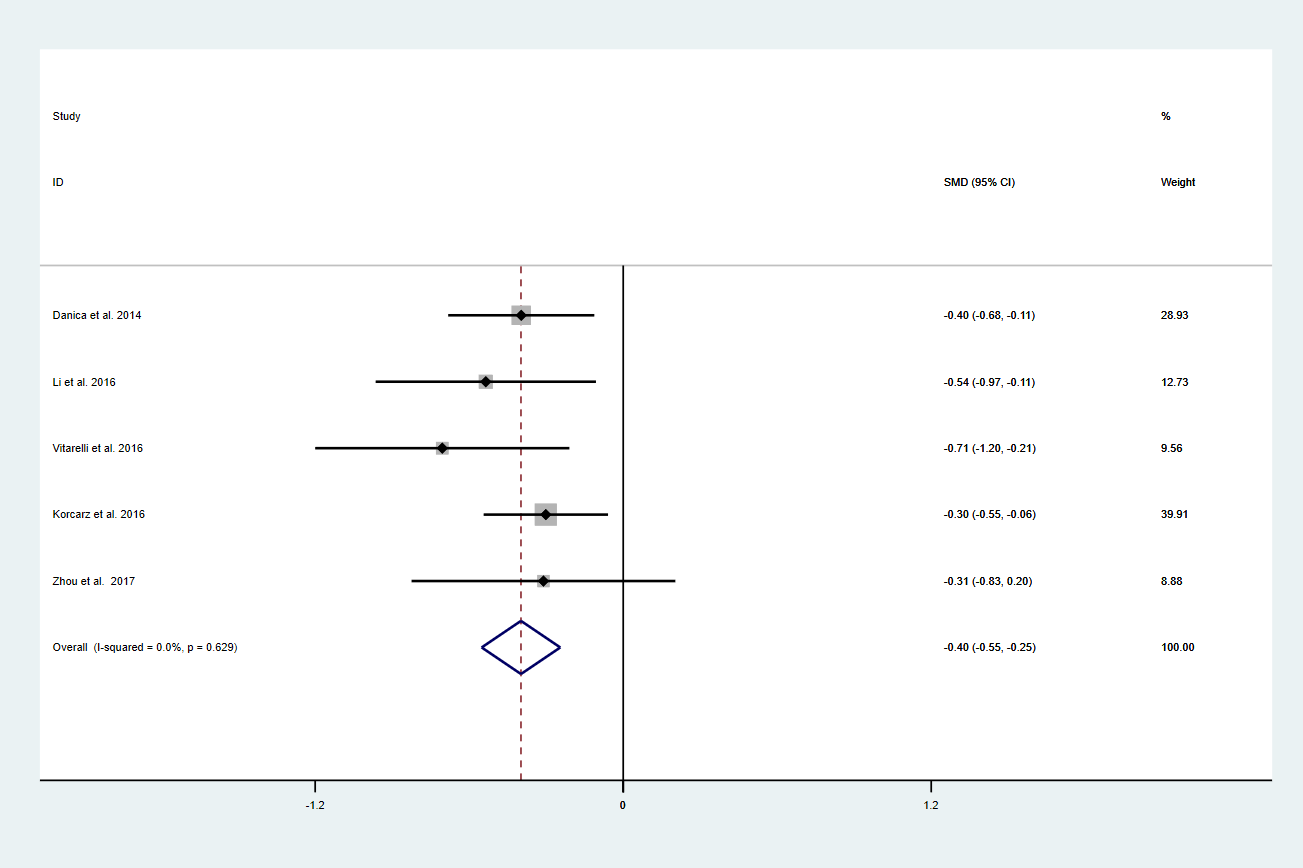


1. **Forest plot of the differences in right ventricular fractional area change between the OSA patients and controls based on echocardiography**
