# Supplementary material for: Obstructive sleep apnea increases the risk of cardiovascular damage: a systematic review and meta-analysis of imaging studies
Source: Syst Rev. 2021 Jul 30;10:212. doi: 10.1186/s13643-021-01759-6 (PMC8325188; doi:10.1186/s13643-021-01759-6)
Supplement: Supplementary file 1 — Additional file 1: Table S1 A example of search strategies. Table S2 Equations. Table S3 Certainty of evidence. Figure S1 Forest plot. Figure S2 Funnel plot. Figure S3 Sensitivity plot. [file 13643_2021_1759_MOESM1_ESM.zip › Figure S2 Funnel plotsR1.docx]

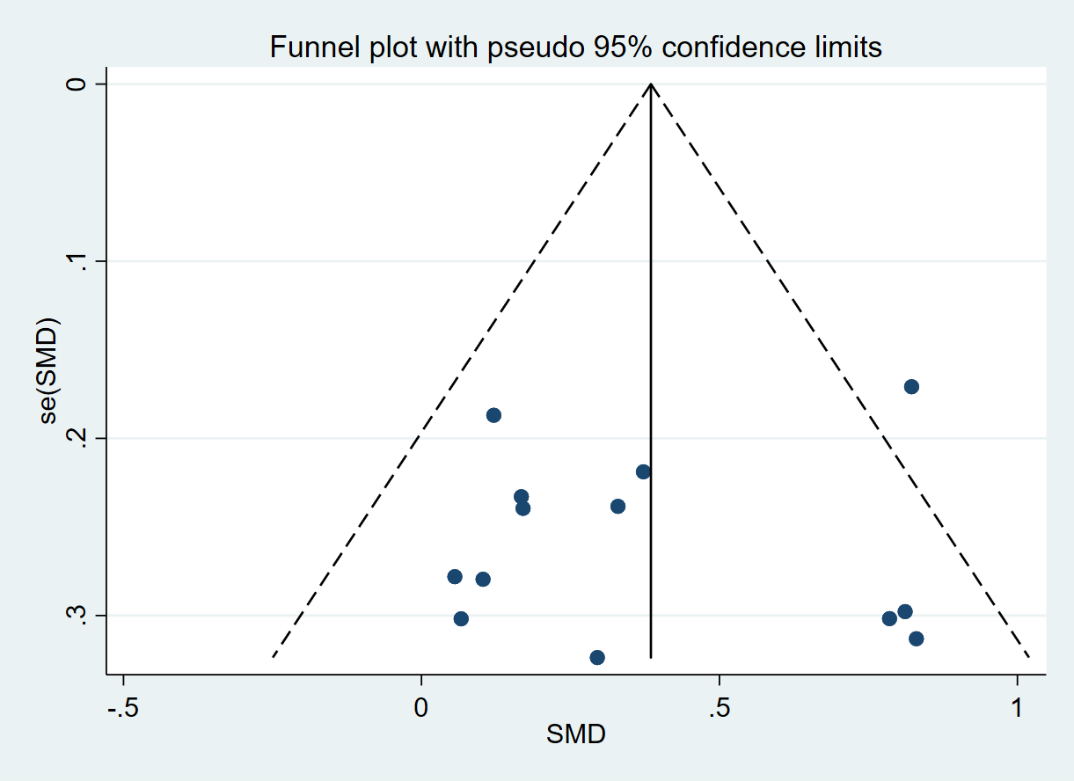


1. Funnel plot for left atrial diameter


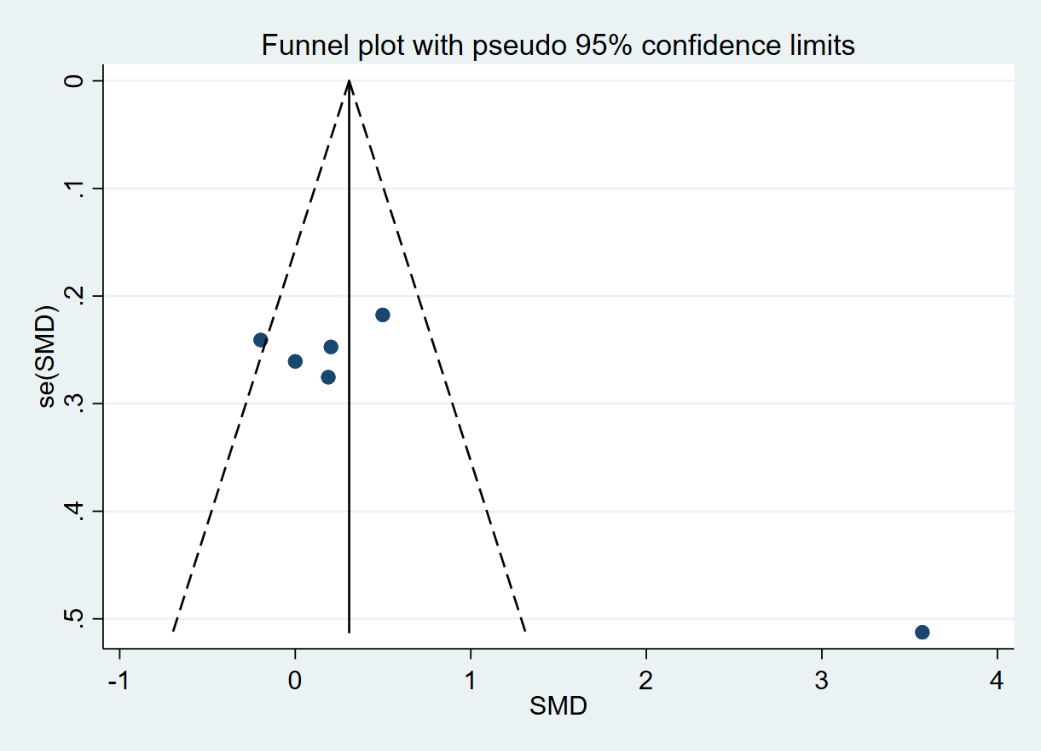


1. Funnel plot for left atrium volume index


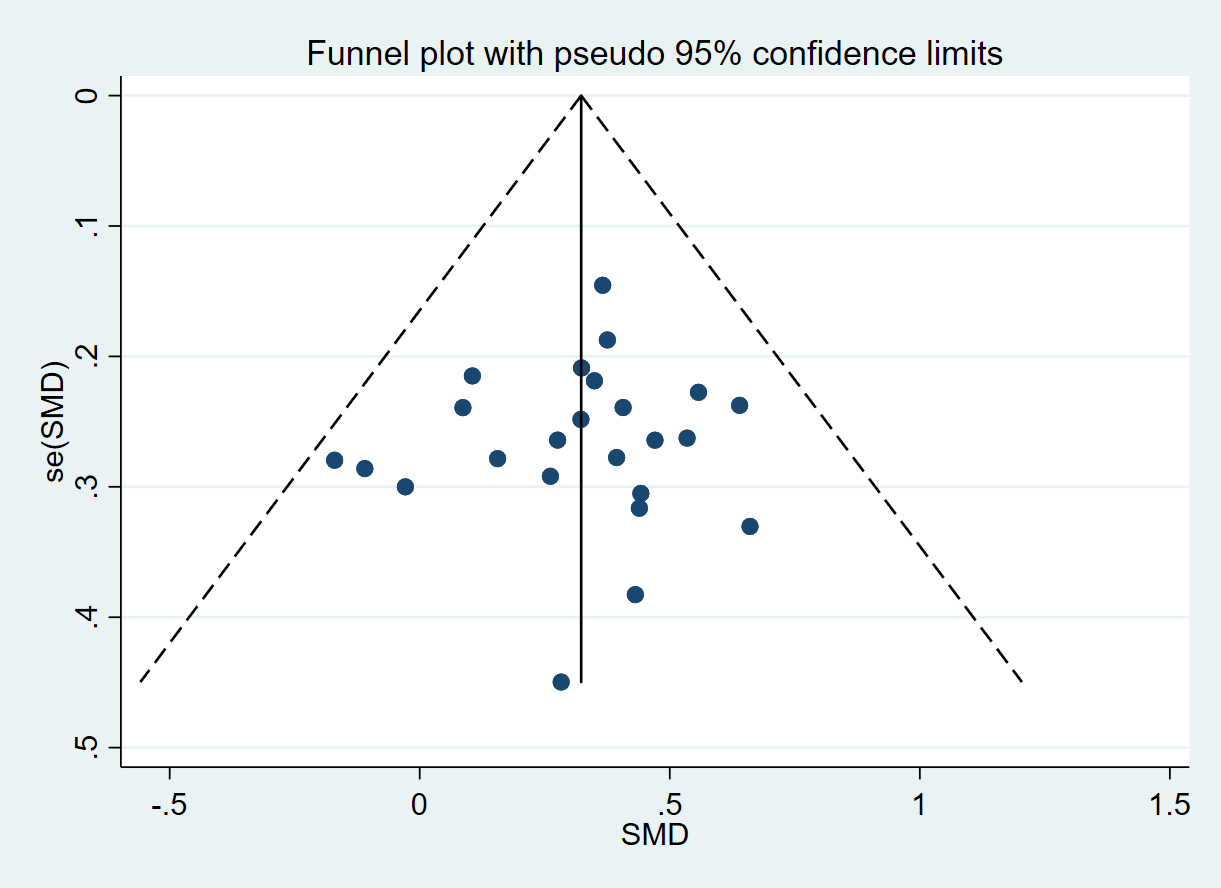


1. Funnel plot for left ventricular end-systolic diameter


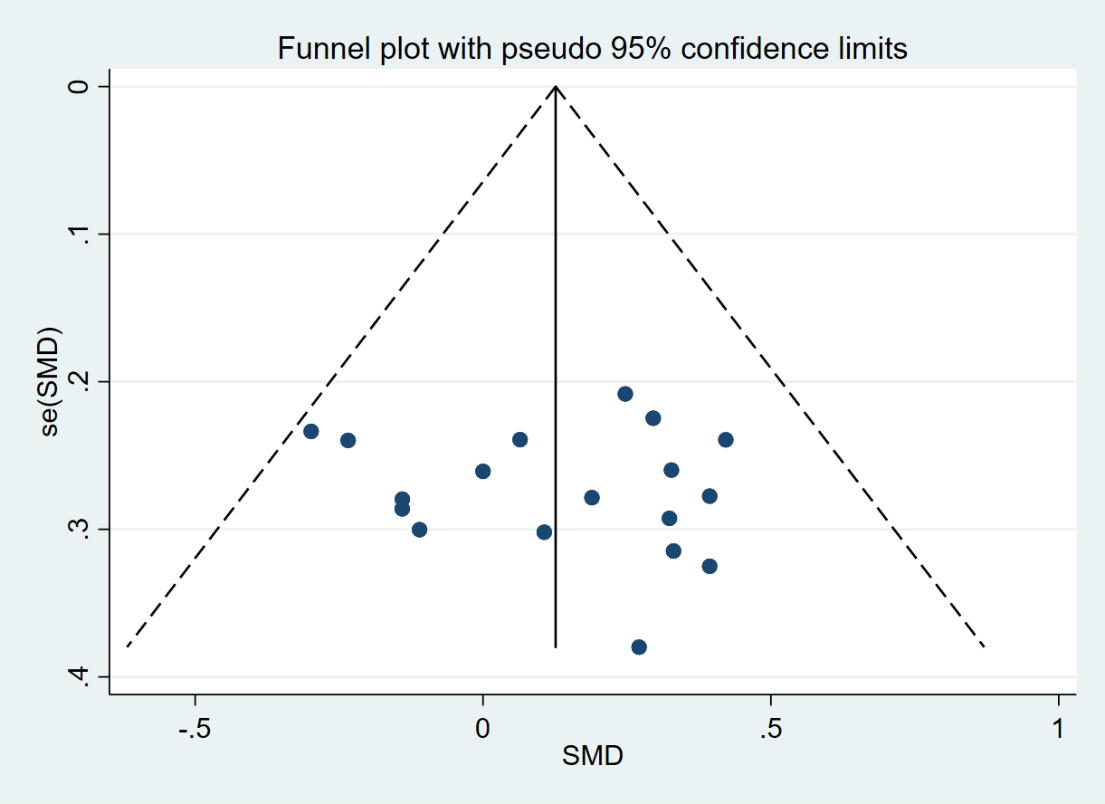


1. Funnel plot for left ventricular end- diastolic diameter


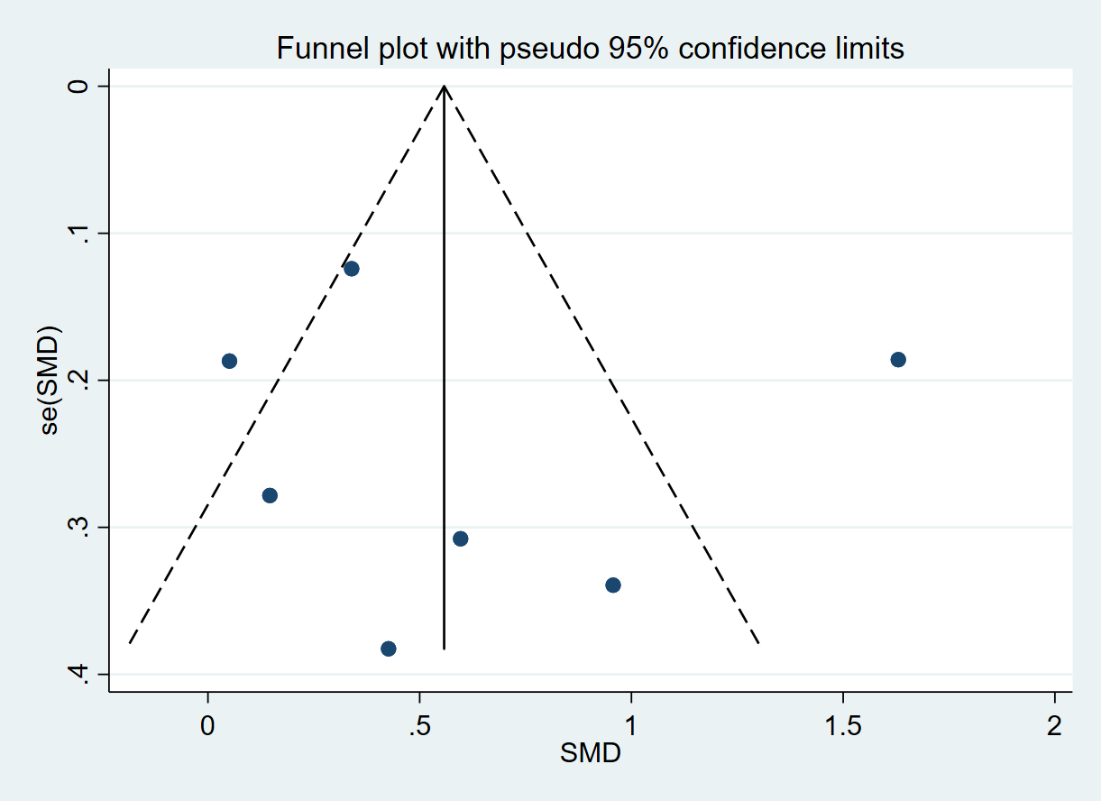


1. Funnel plot for left ventricular mass


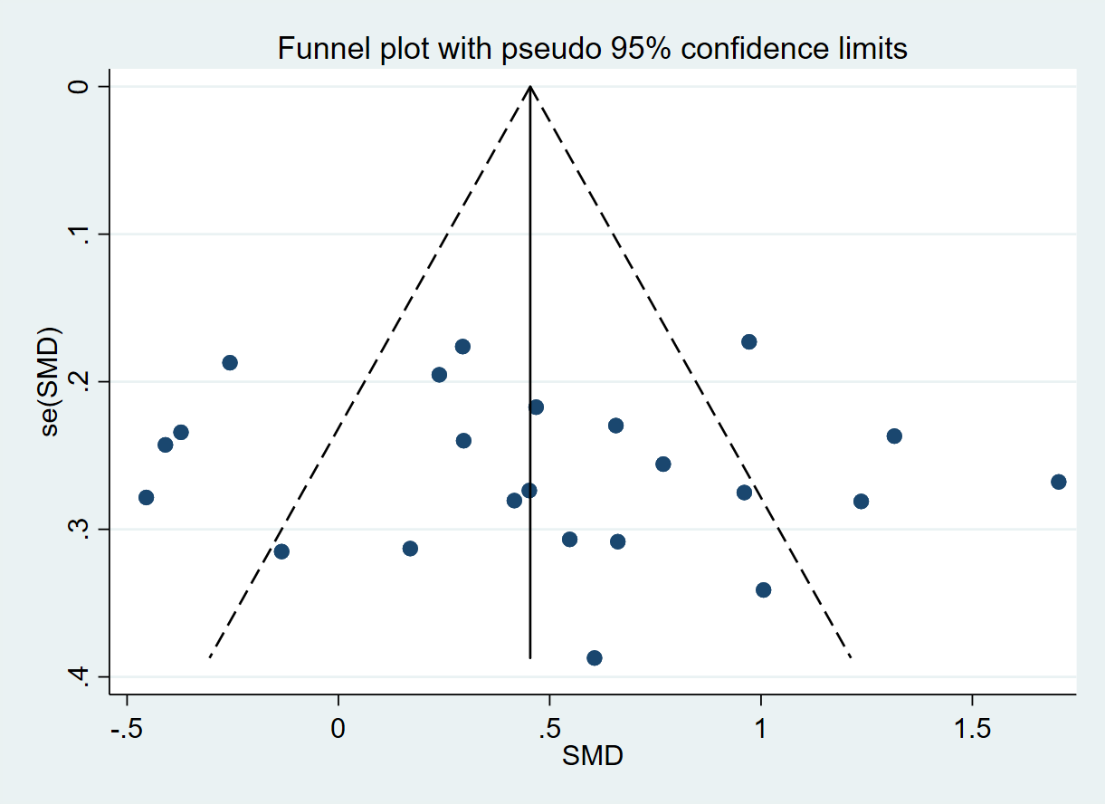


1. Funnel plot for left ventricular mass index


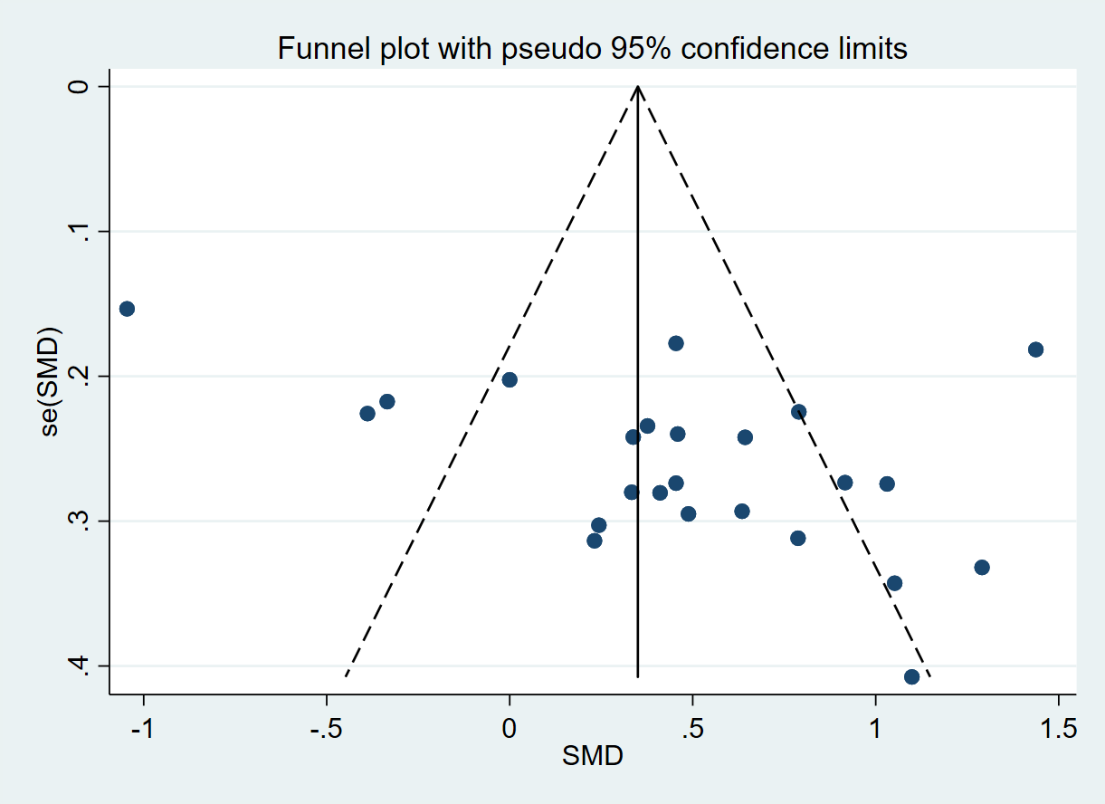


1. Funnel plot for interventricular septum diameter


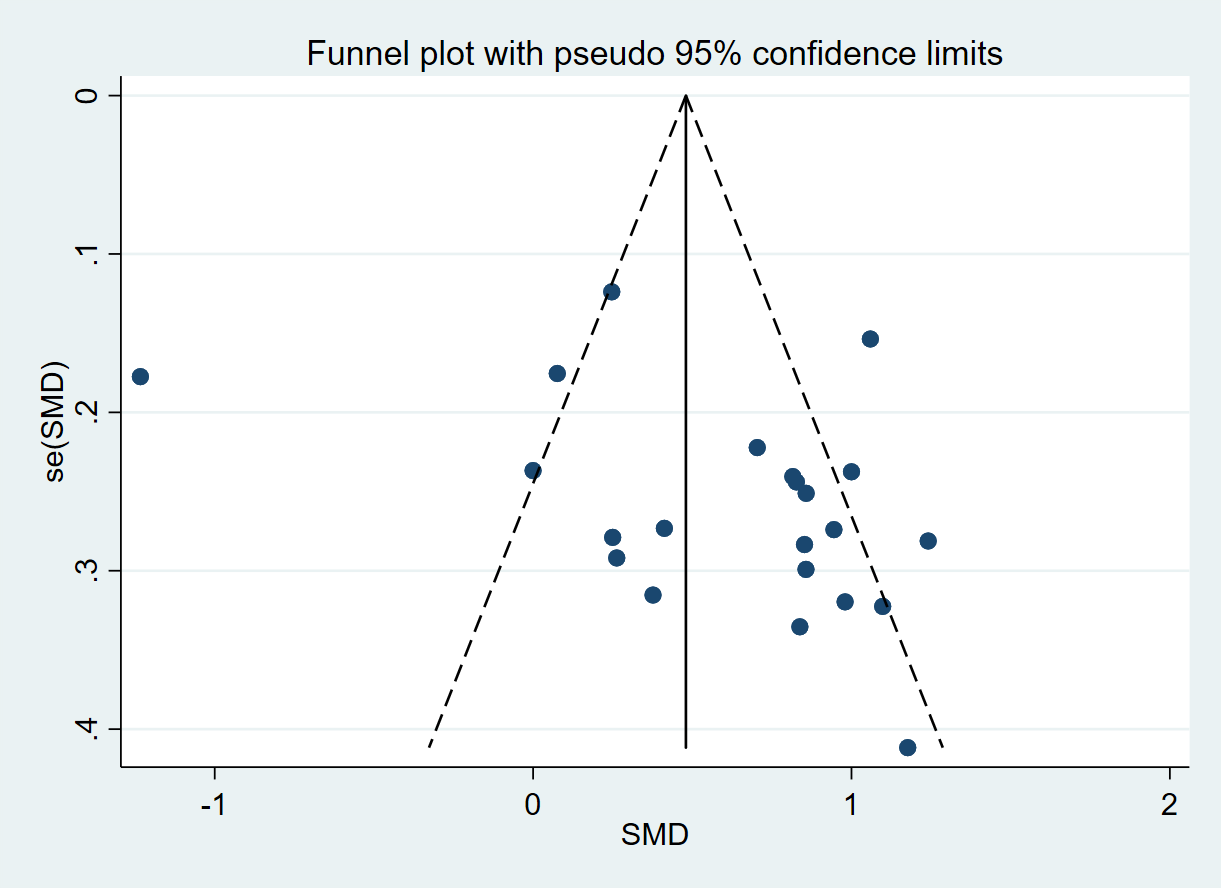


1. Funnel plot for posterior wall diameter


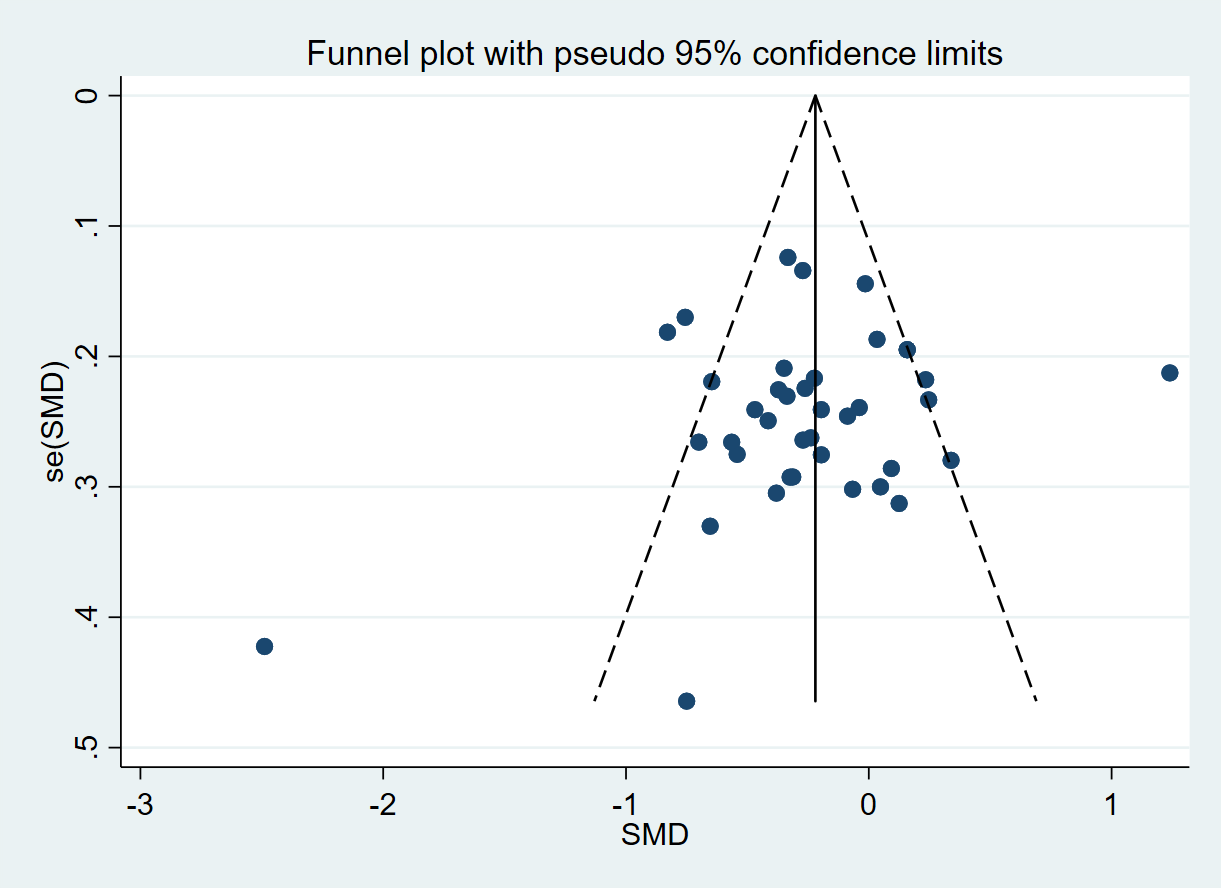


1. Funnel plot for left ventricular ejection fraction


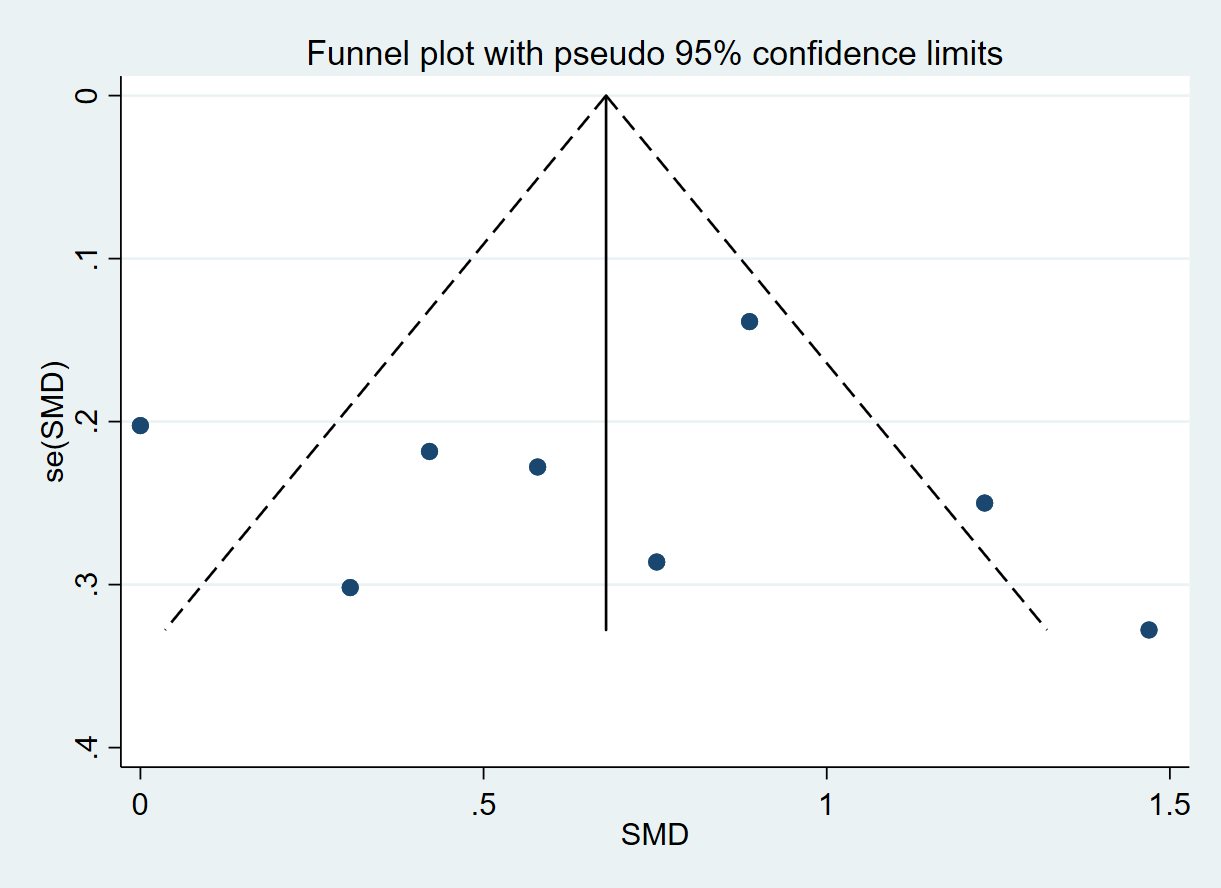


1. Funnel plot for left ventricular myocardial performance index


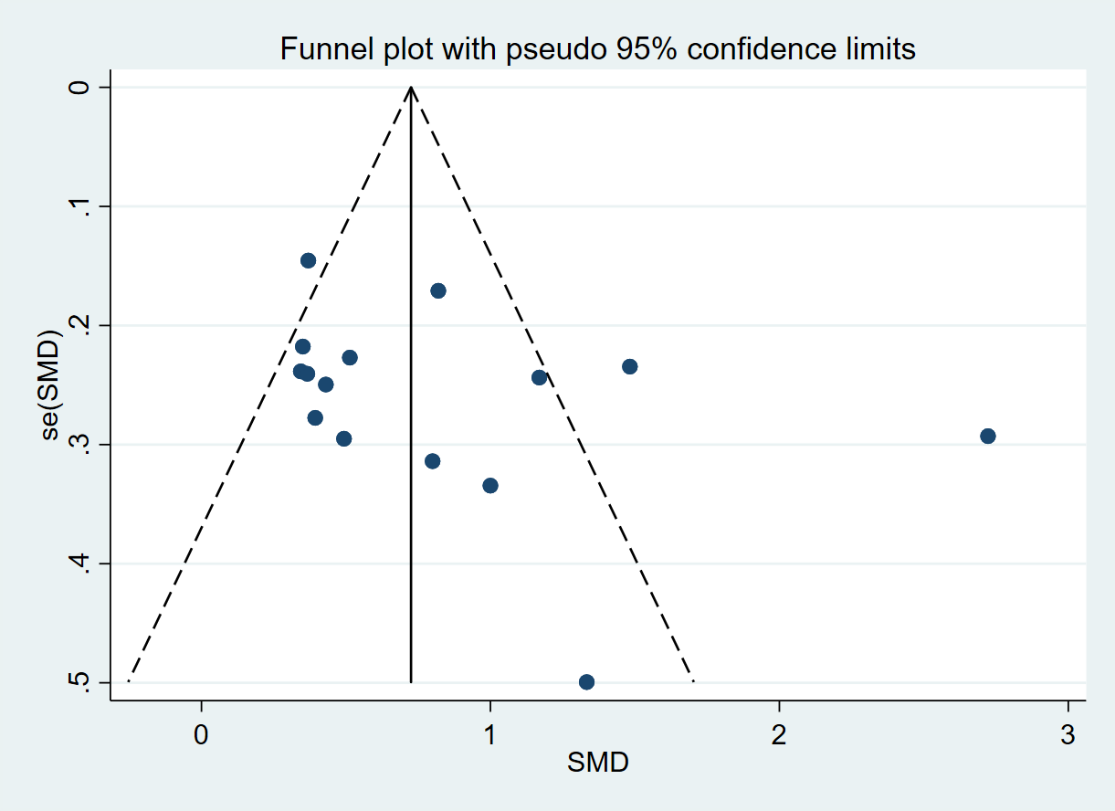


1. Funnel plot for right ventricular diameter


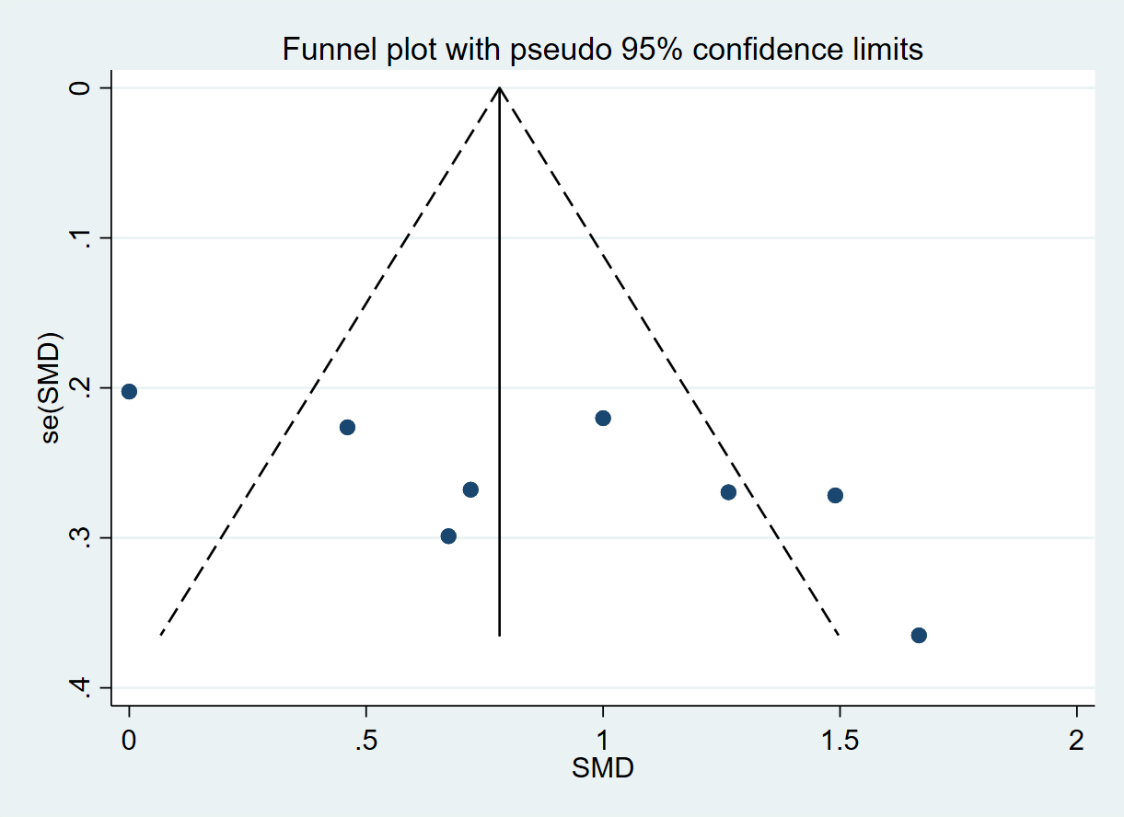


1. Funnel plot for right ventricular myocardial performance index


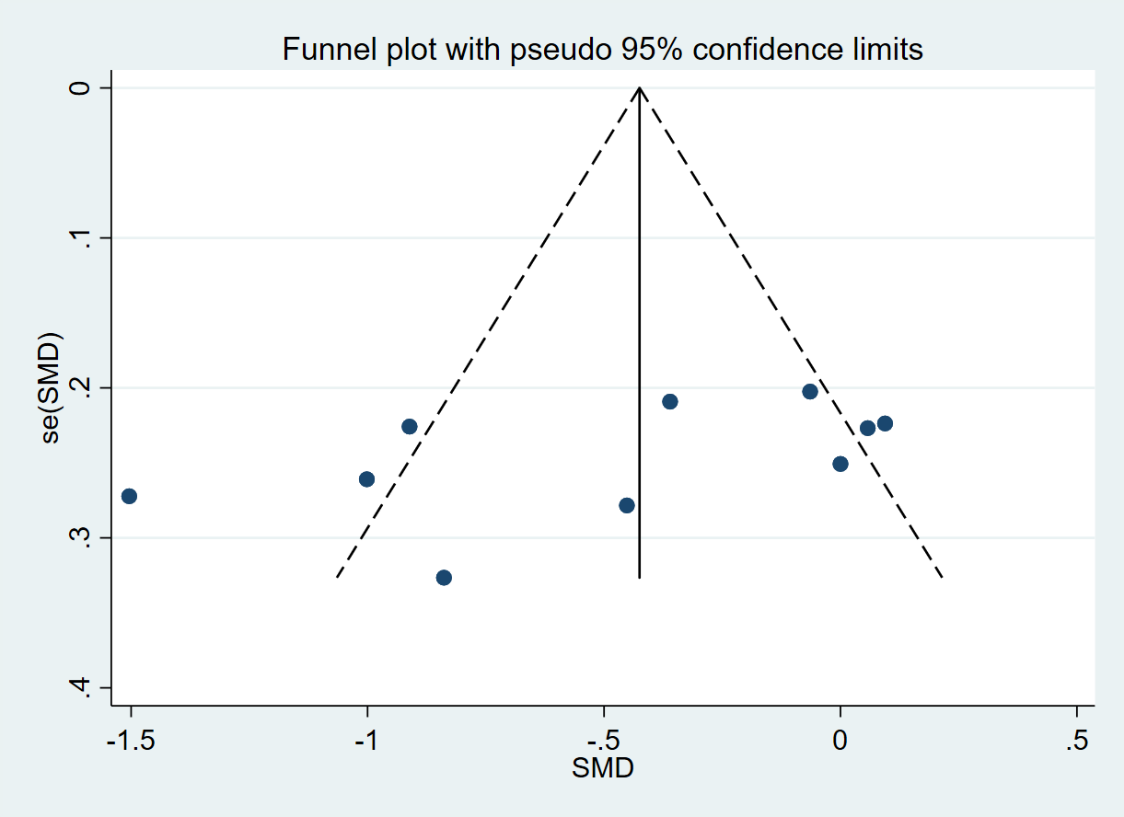


1. Funnel plot for tricuspid annular plane systolic excursion
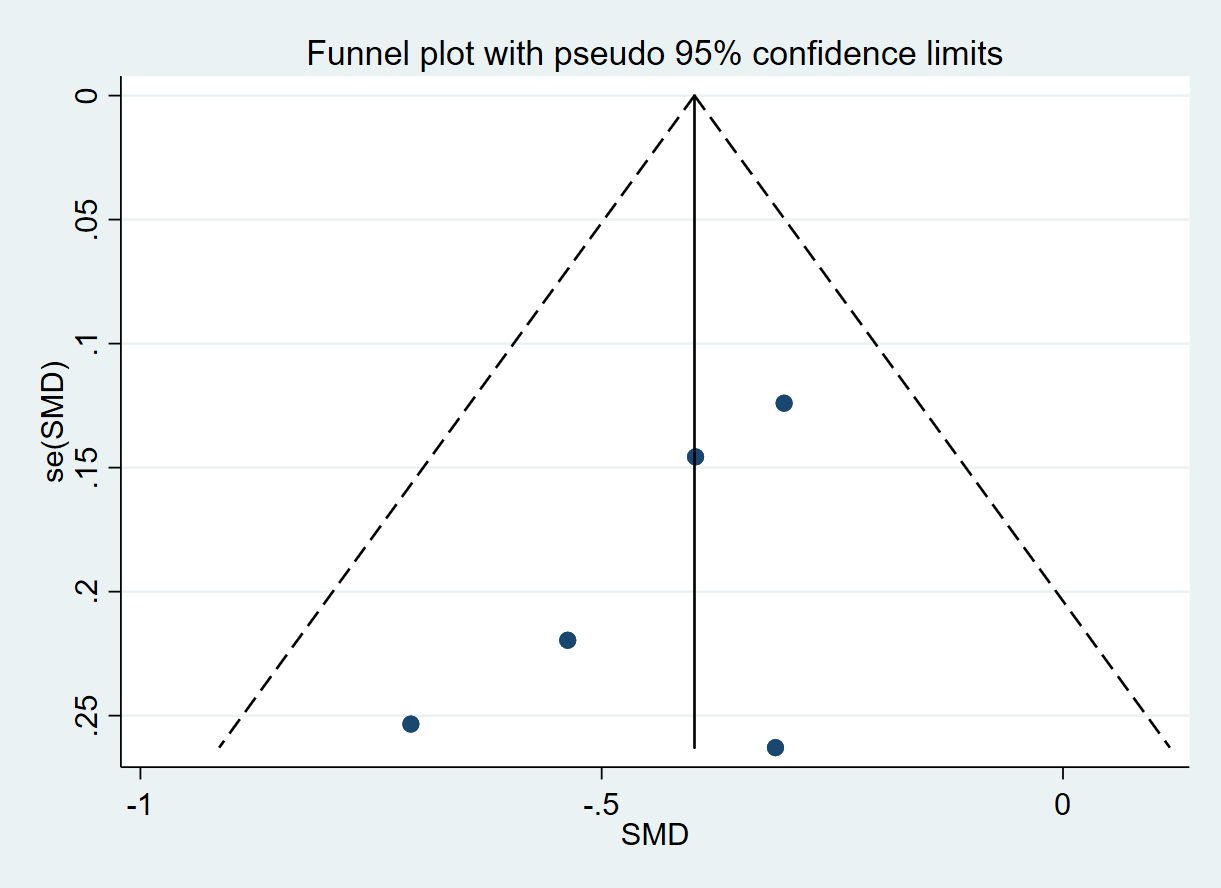

2. Funnel plot for right ventricular fractional area change
