# Supplementary material for: Obstructive sleep apnea increases the risk of cardiovascular damage: a systematic review and meta-analysis of imaging studies
Source: Syst Rev. 2021 Jul 30;10:212. doi: 10.1186/s13643-021-01759-6 (PMC8325188; doi:10.1186/s13643-021-01759-6)
Supplement: Supplementary file 1 — Additional file 1: Table S1 A example of search strategies. Table S2 Equations. Table S3 Certainty of evidence. Figure S1 Forest plot. Figure S2 Funnel plot. Figure S3 Sensitivity plot. [file 13643_2021_1759_MOESM1_ESM.zip › Figure S3 Sensitivity plotsR1.docx]

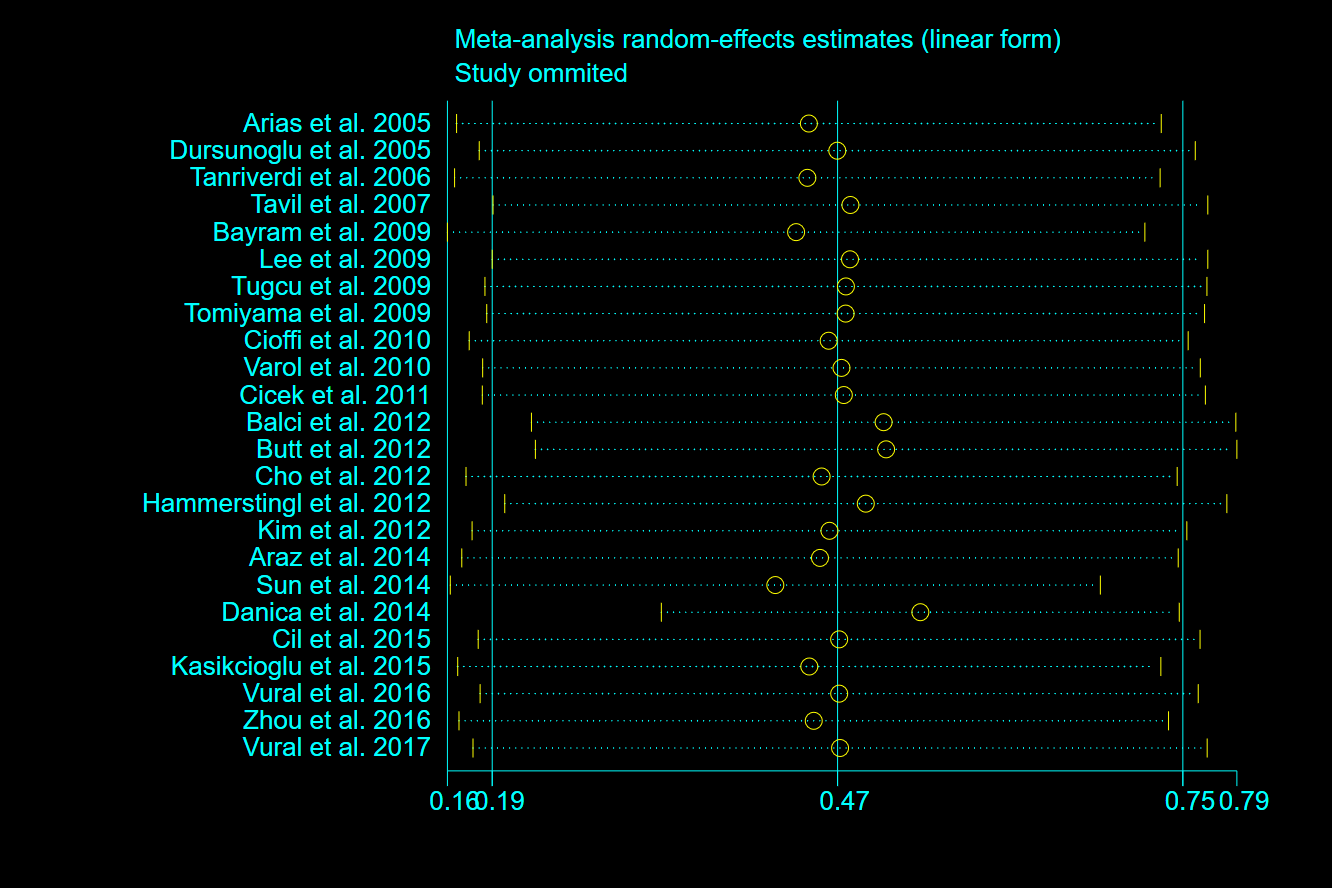


1. Sensitivity analysis of studies on interventricular septum diameter


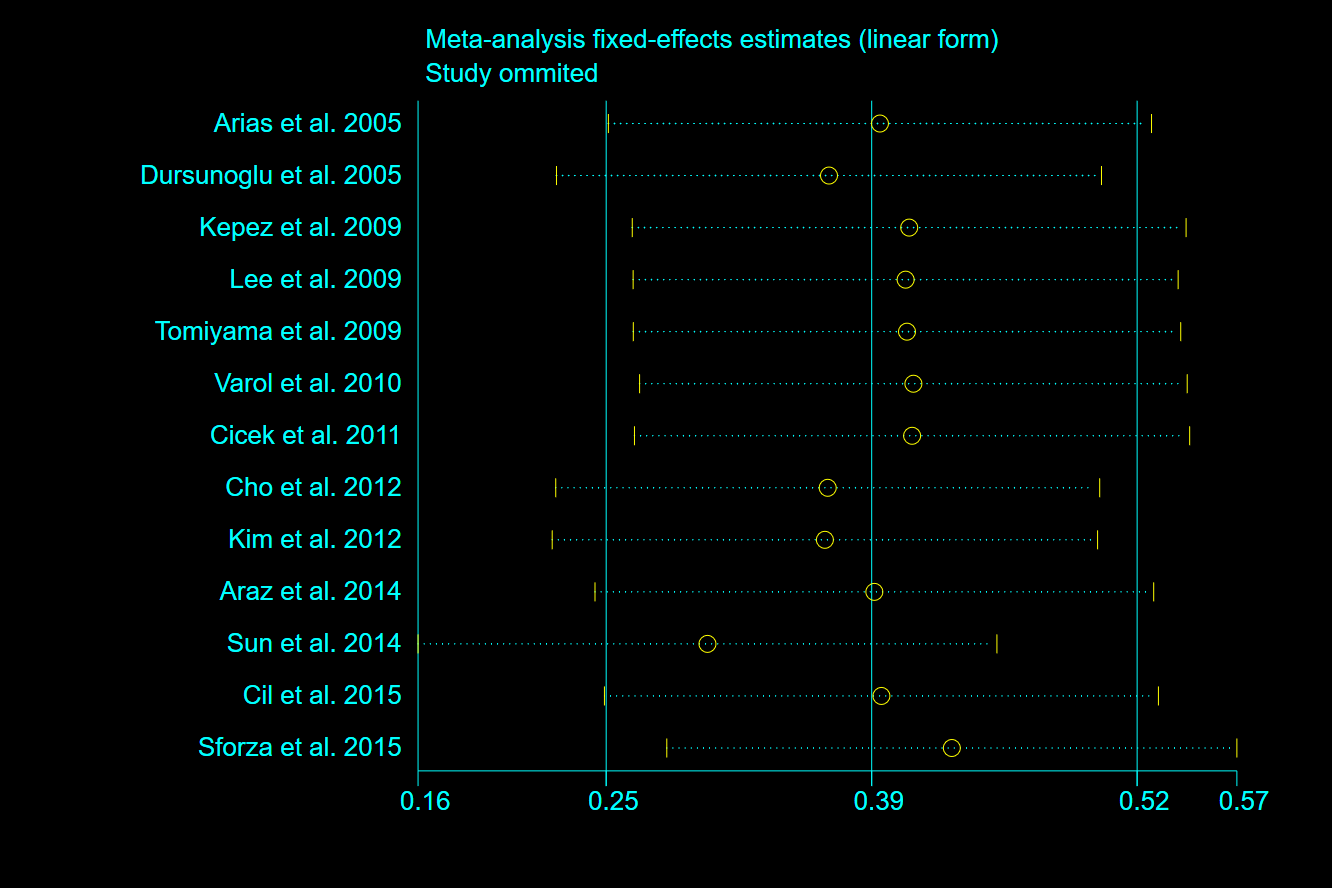


1. Sensitivity analysis of studies on left atrial diameter


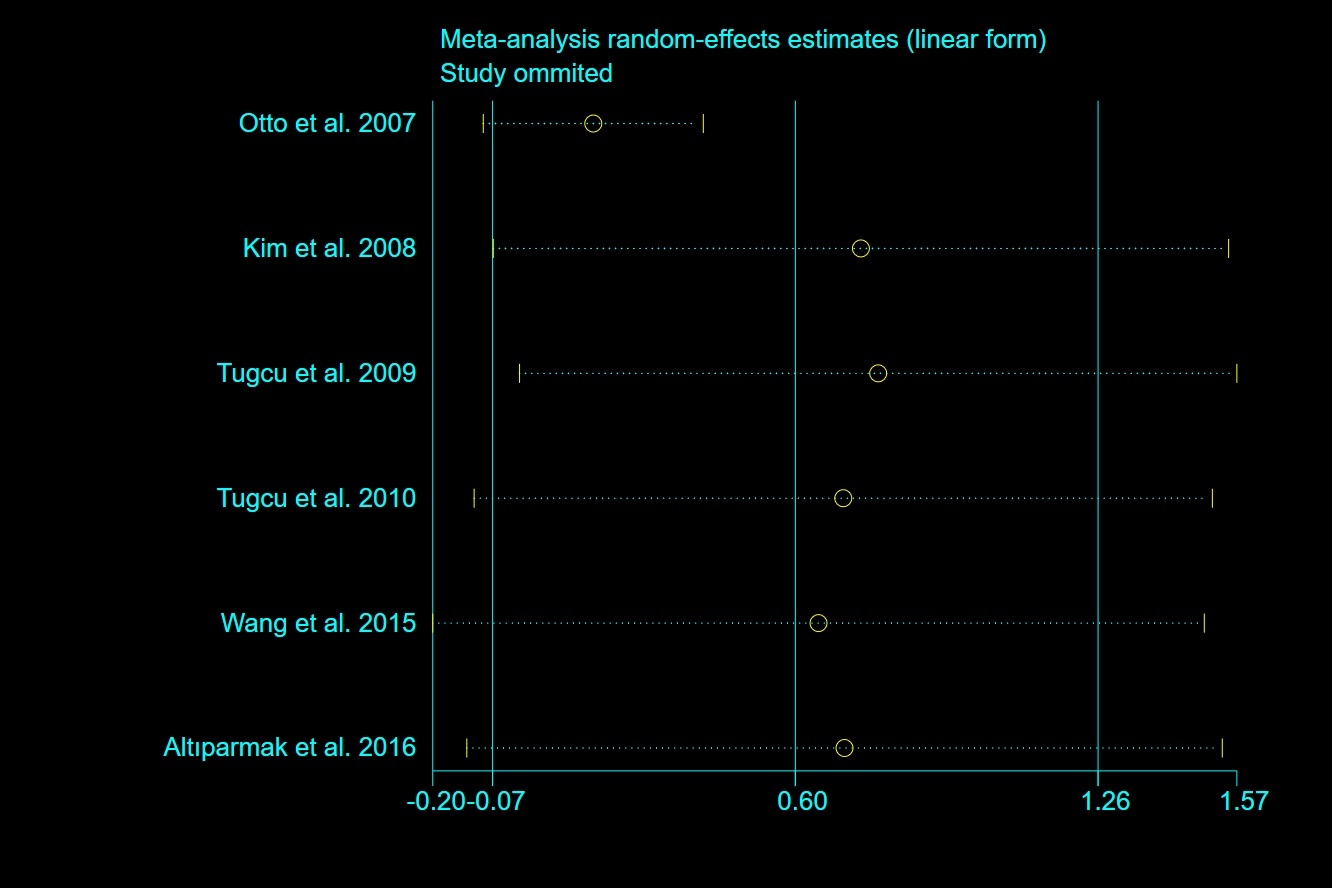


1. Sensitivity analysis of studies on left atrium volume index


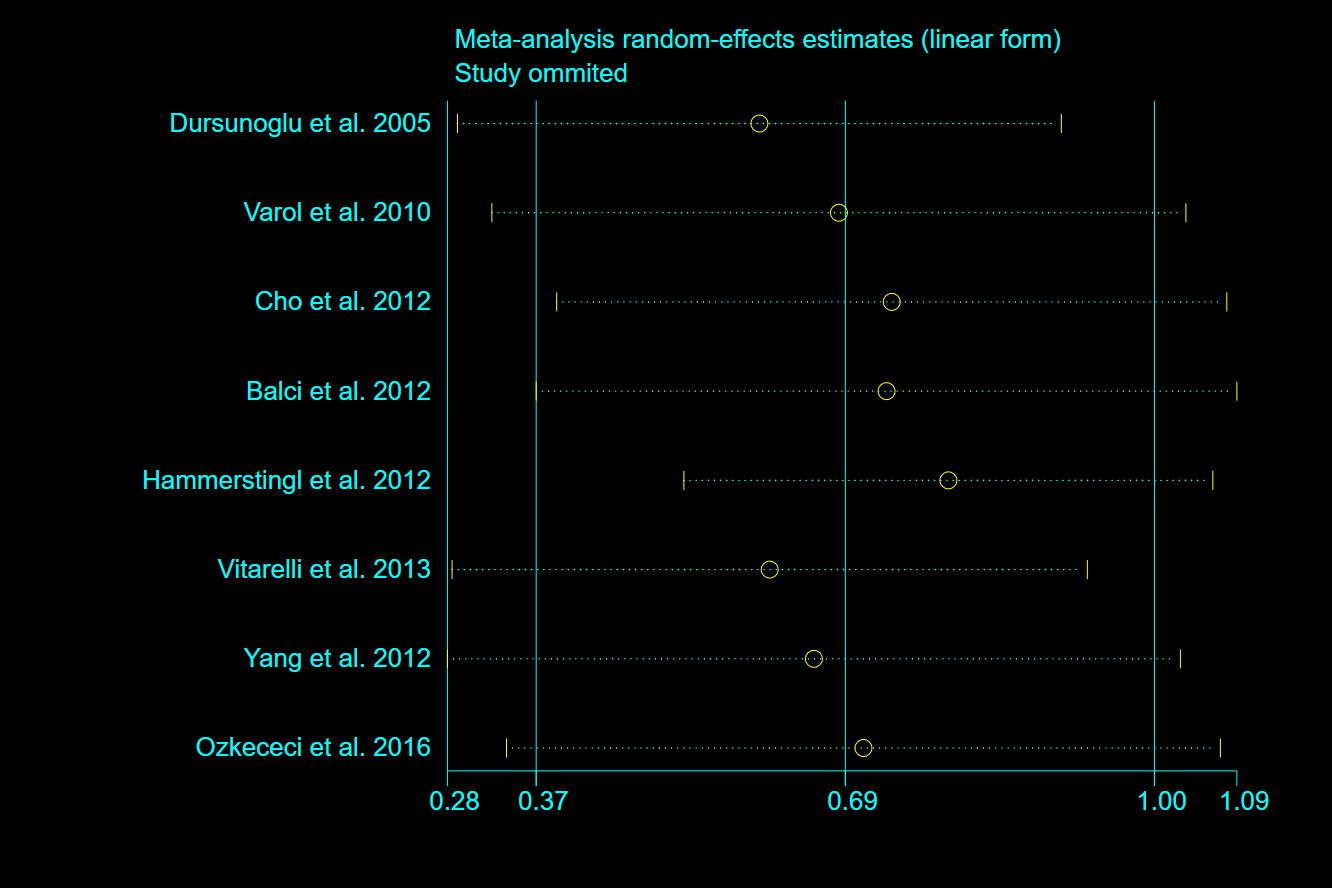


1. Sensitivity analysis of studies on left ventricular myocardial performance index


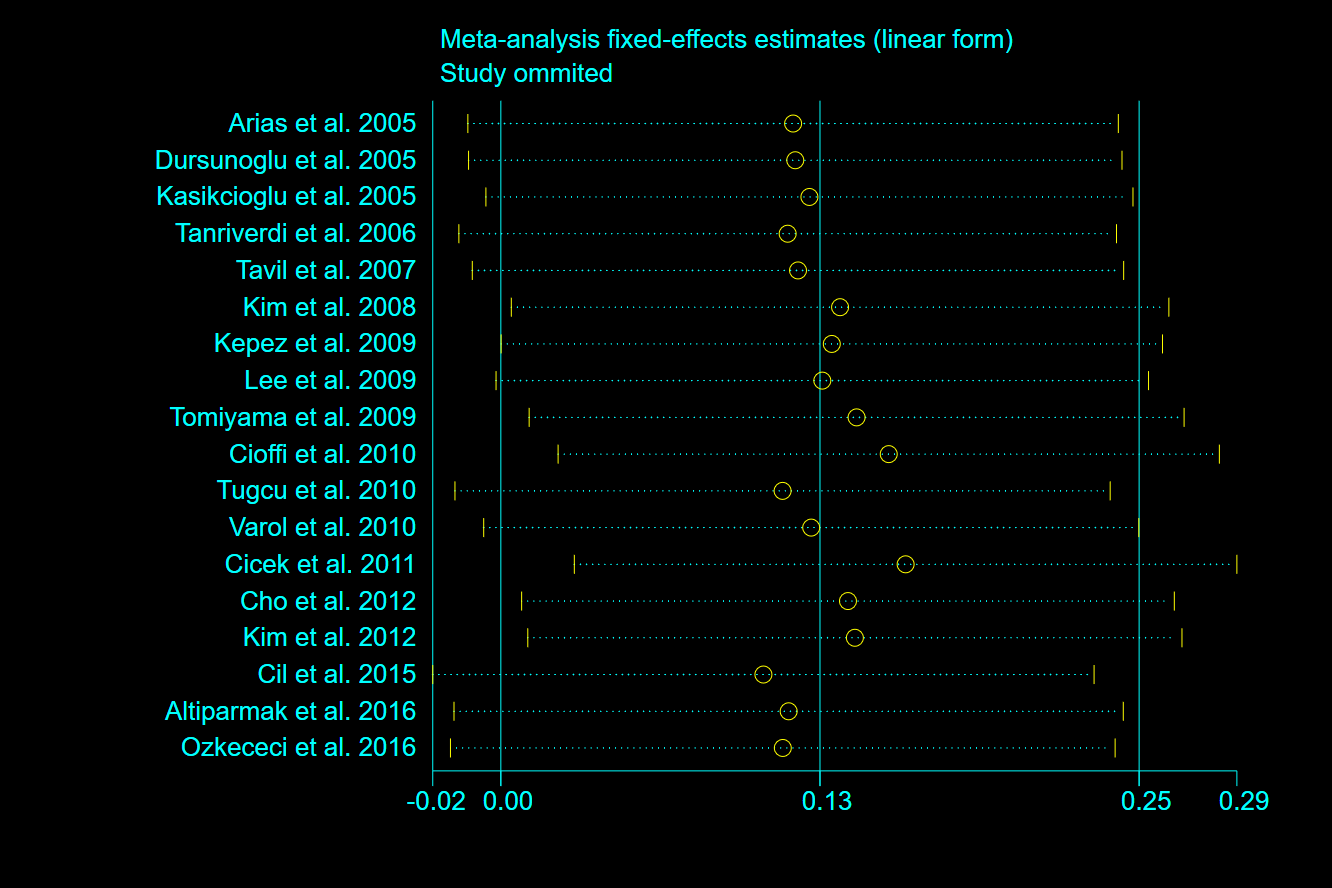


e. Sensitivity analysis of studies on left ventricular end- diastolic diameter


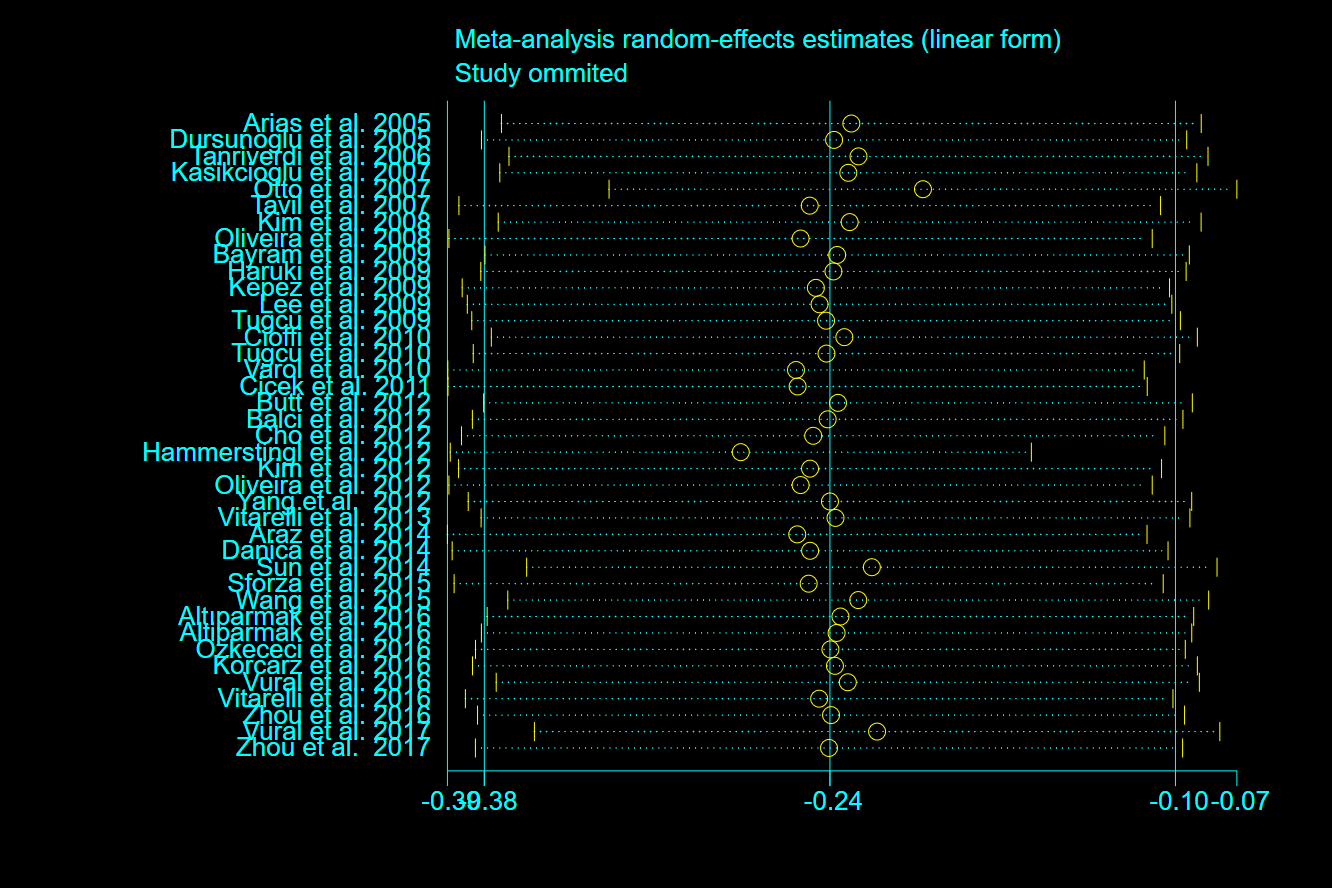


f. Sensitivity analysis of studies on left ventricular ejection fraction


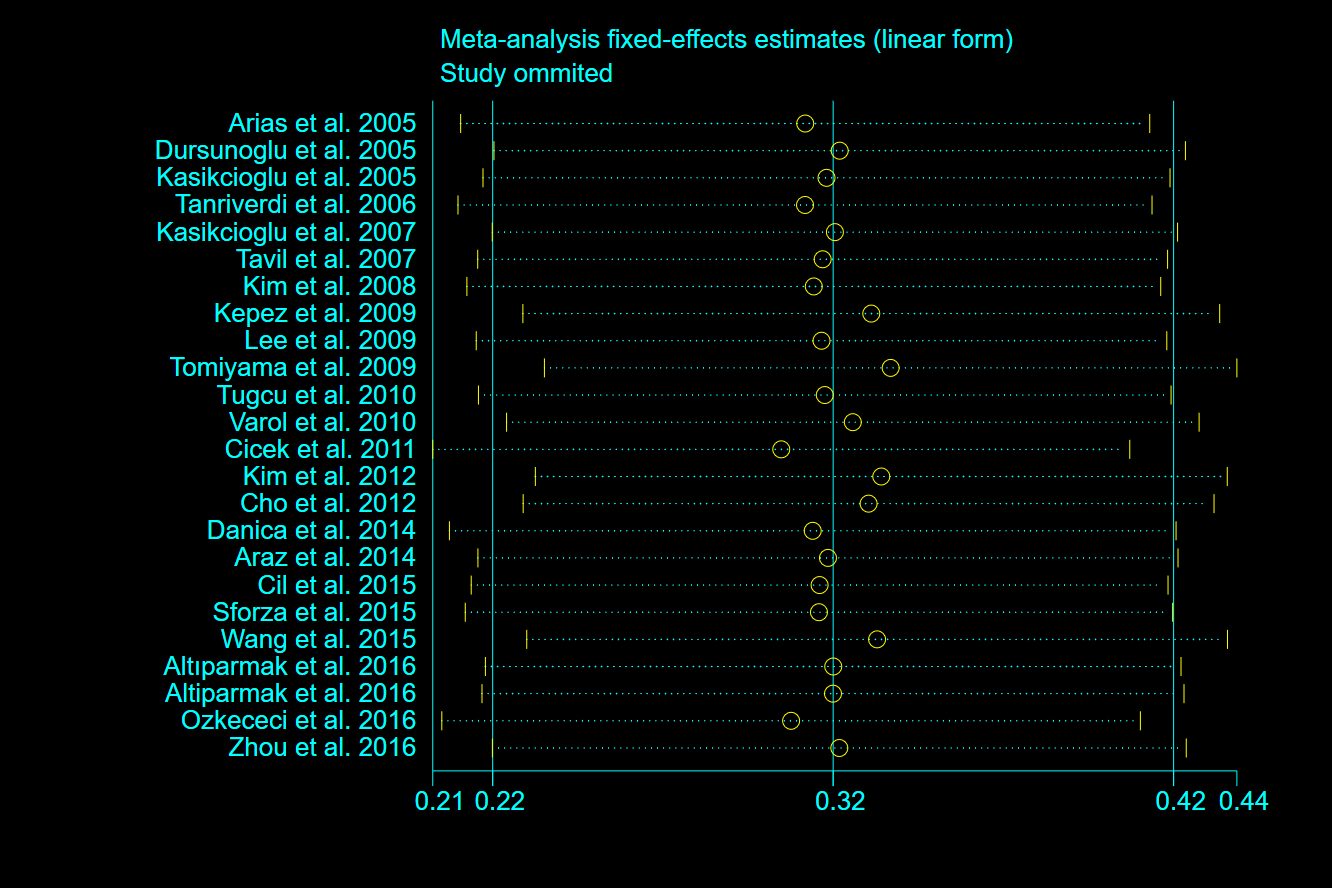


g. Sensitivity analysis of studies on left ventricular end-systolic diameter


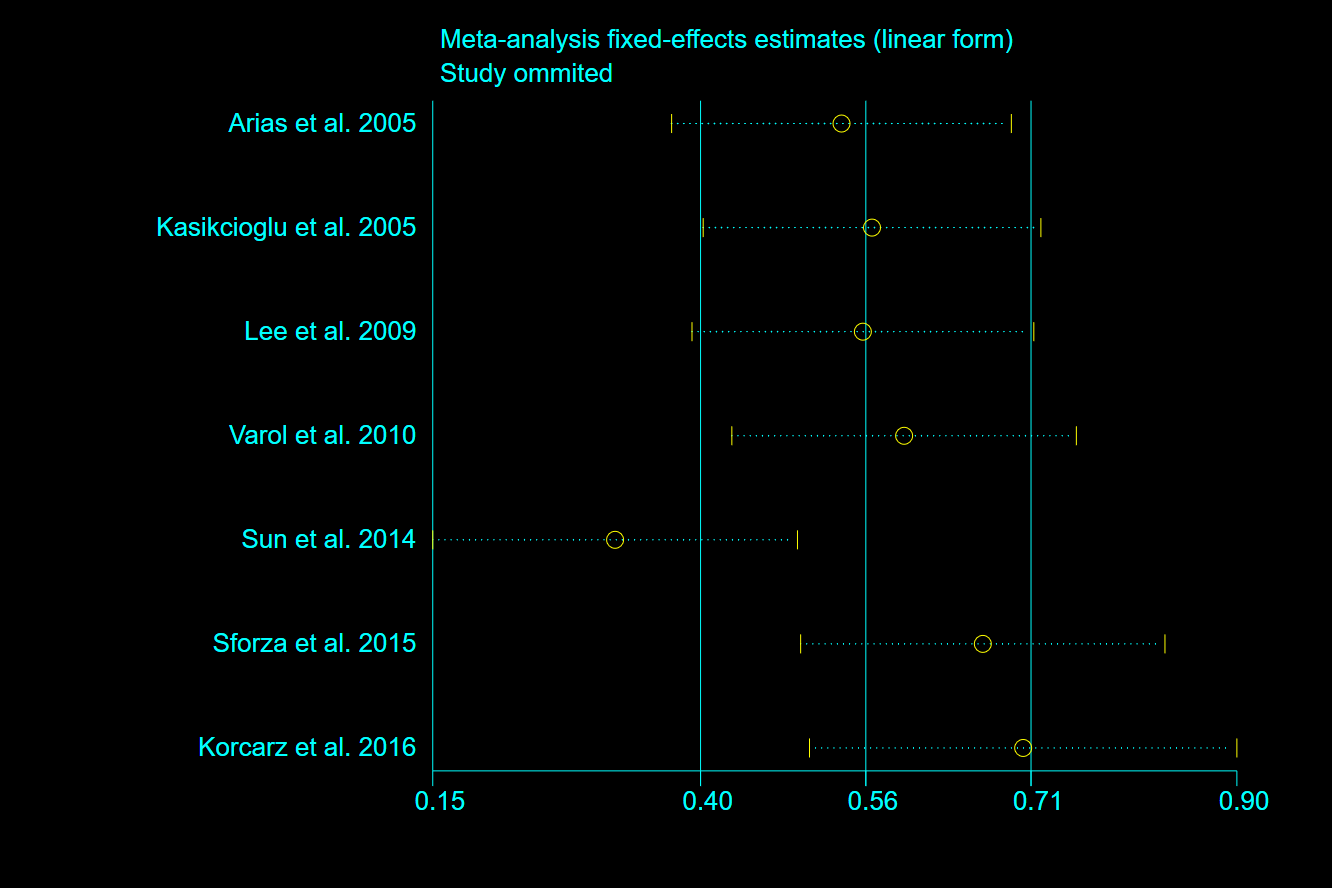


h. Sensitivity analysis of studies on left ventricular mass


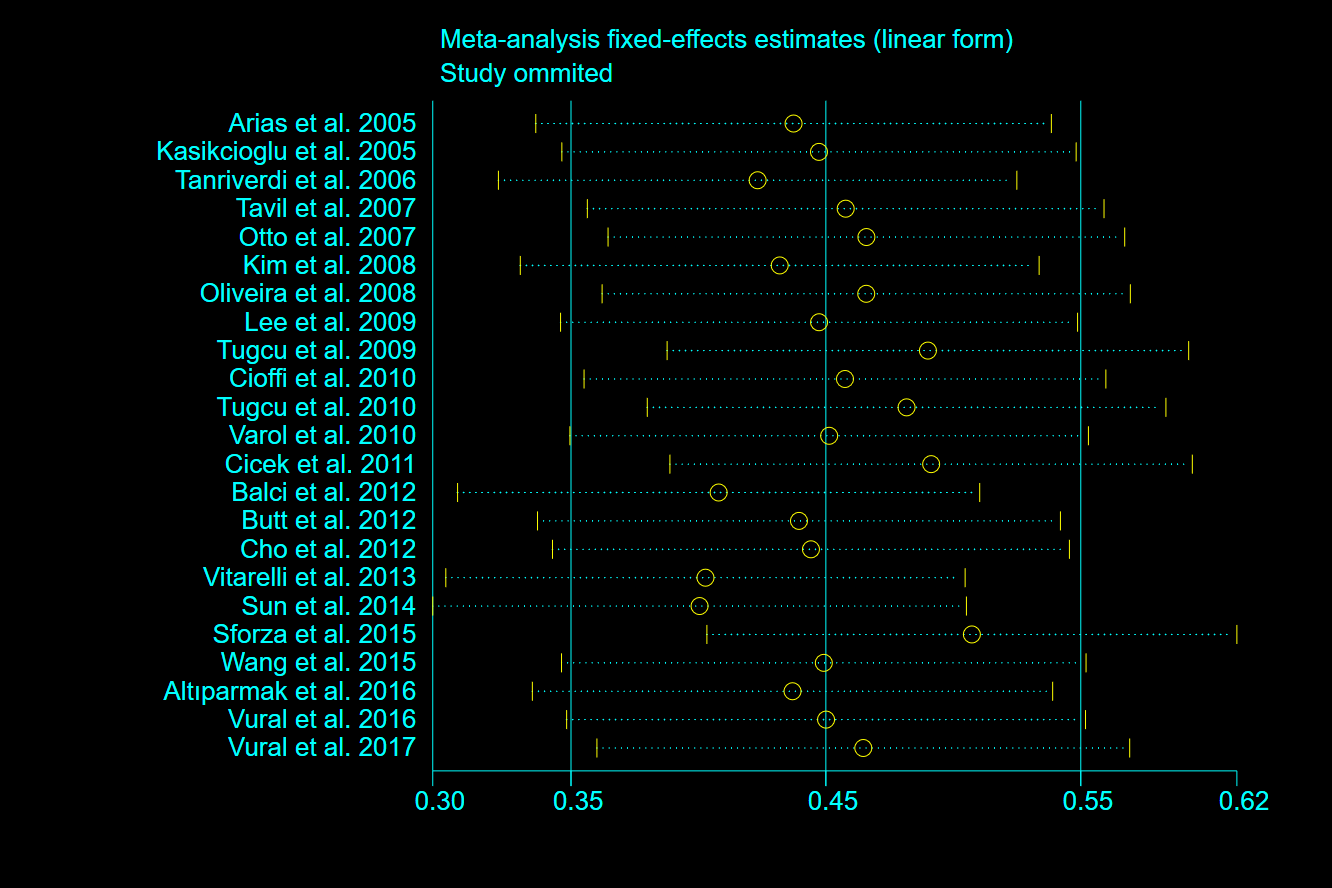


i. Sensitivity analysis of studies on left ventricular mass index


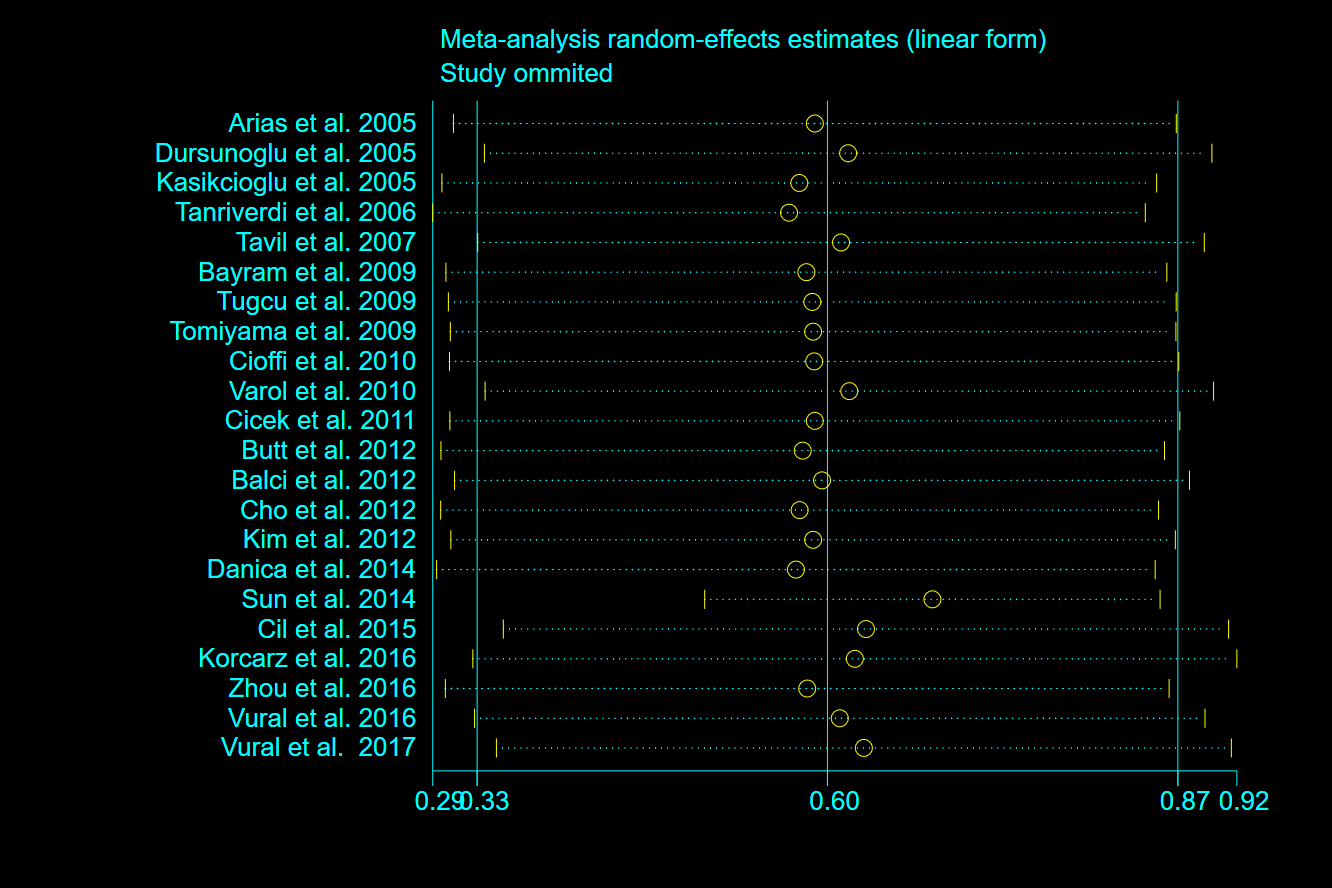


j. Sensitivity analysis of studies on posterior wall diameter


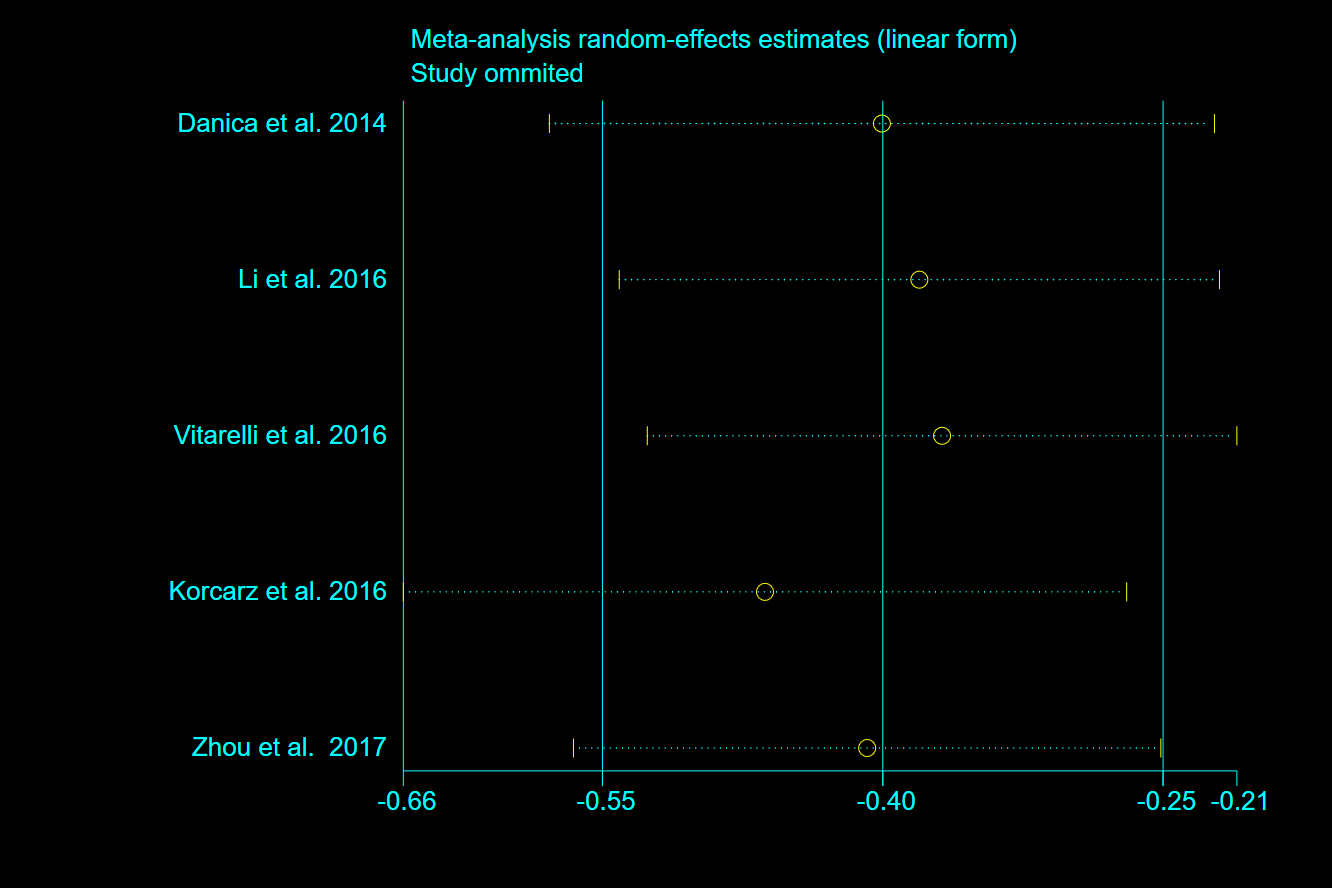


k. Sensitivity analysis of studies on right ventricular fractional area change


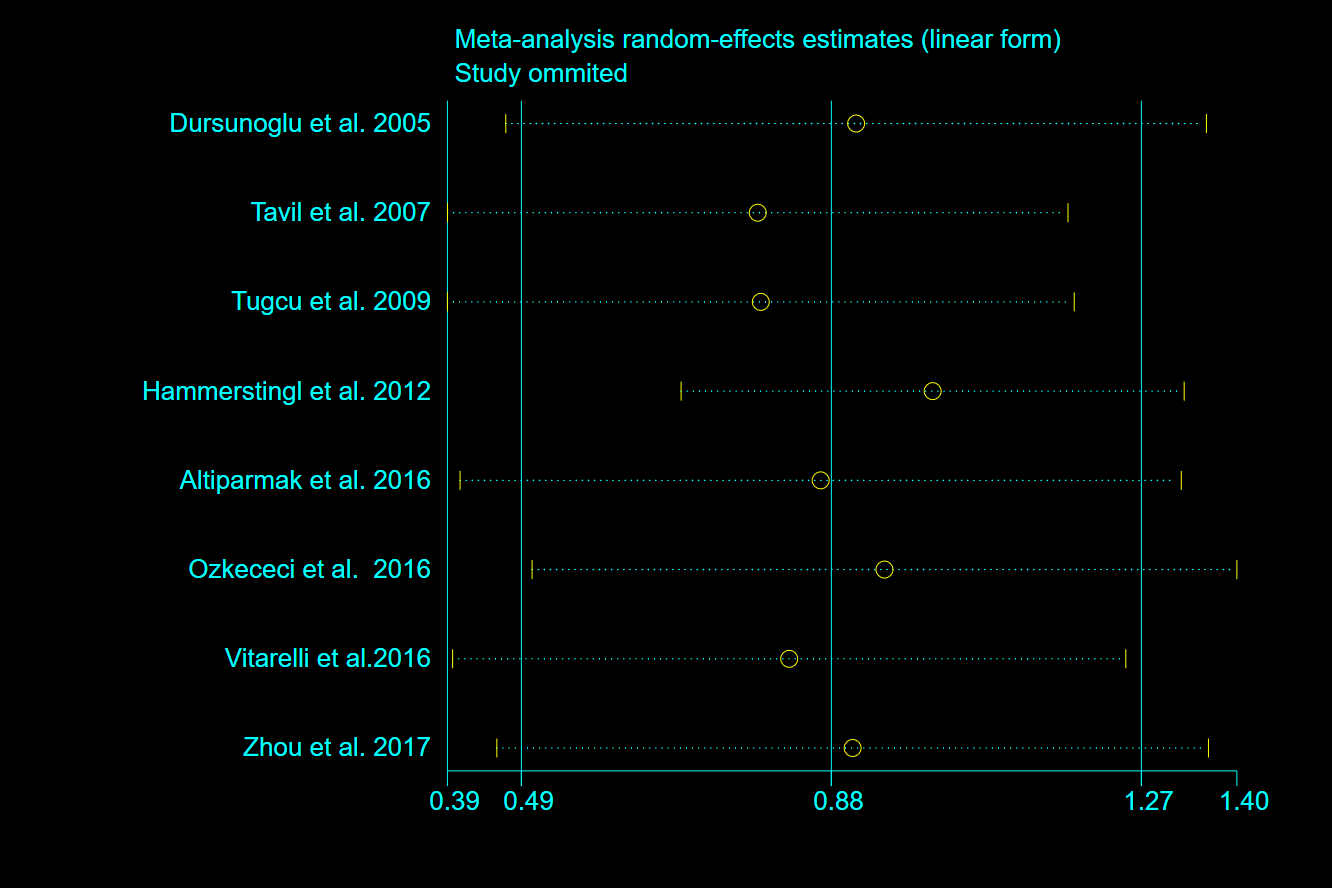


l. Sensitivity analysis of studies on right ventricular myocardial performance index


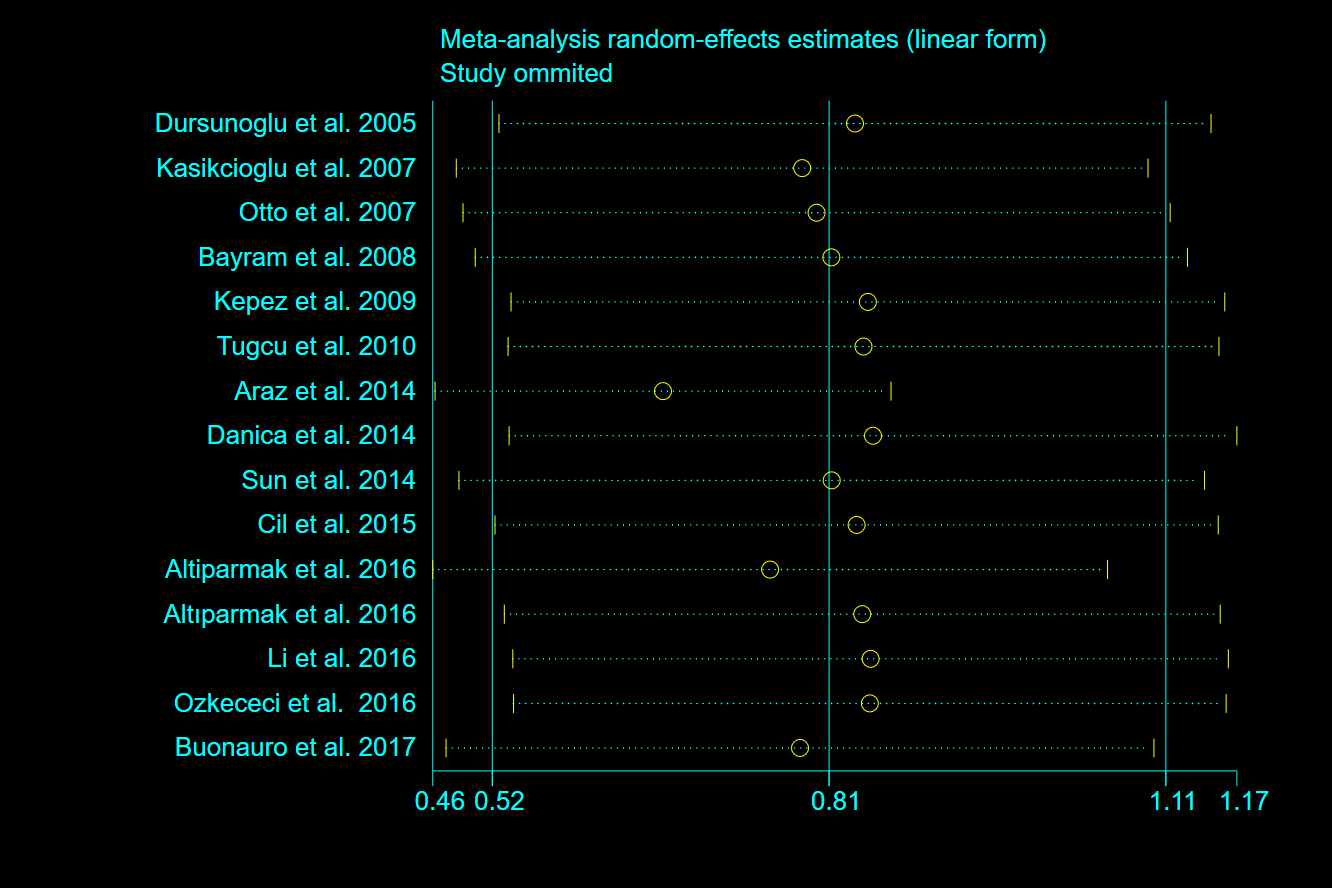


m. Sensitivity analysis of studies on right ventricular diameter


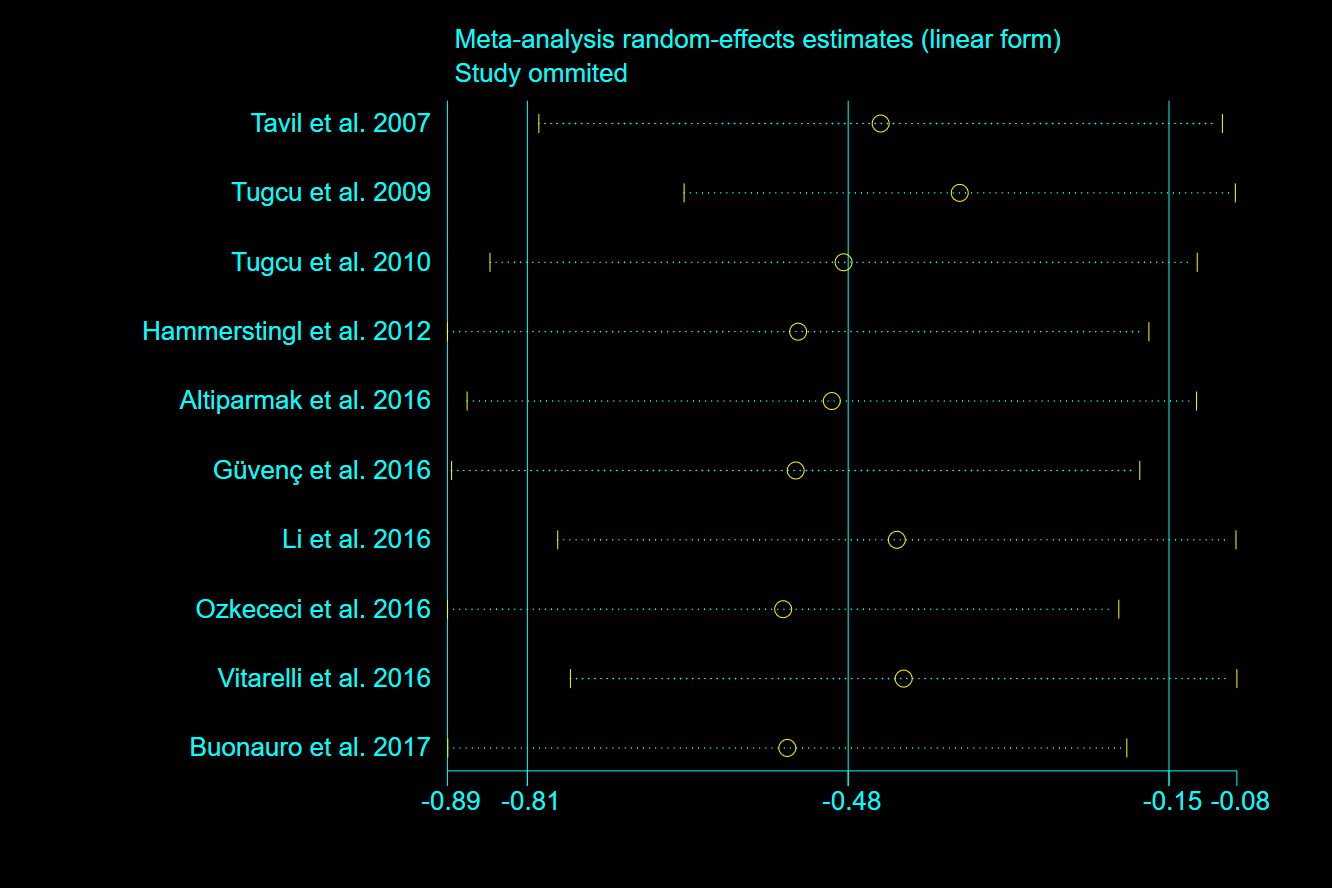


n. Sensitivity analysis of studies on tricuspid annular plane systolic excursion
