# Supplementary material for: Obstructive sleep apnea increases the risk of cardiovascular damage: a systematic review and meta-analysis of imaging studies
Source: Syst Rev. 2021 Jul 30;10:212. doi: 10.1186/s13643-021-01759-6 (PMC8325188; doi:10.1186/s13643-021-01759-6)
Supplement: Supplementary file 1 — Additional file 1: Table S1 A example of search strategies. Table S2 Equations. Table S3 Certainty of evidence. Figure S1 Forest plot. Figure S2 Funnel plot. Figure S3 Sensitivity plot. [file 13643_2021_1759_MOESM1_ESM.zip › Table S1 A example of search strategiesR1.docx]

**Table S1 A example of search strategies**

**PubMed**

((Sleep Apnea, Obstructive[MeSH Terms]) OR (Apnea, Obstructive Sleep) OR (Obstructive Sleep Apnea) OR (Obstructive Sleep Apnea Syndrome) OR (OSAHS) OR (Syndrome, Sleep Apnea, Obstructive) OR (Sleep Apnea Syndrome, Obstructive) OR (Sleep Apnea Hypopnea Syndrome) OR (Syndrome, Obstructive Sleep Apnea) OR (Sleep-Disordered Breathing)) AND ((((((((((((Ventricular Function, Left[MeSH Terms]) OR ((Left Ventricular Function) OR (Function, Left Ventricular))) OR (Ventricular Dysfunction, Left[MeSH Terms])) OR ((Left Ventricular Dysfunction) OR (Dysfunction, Left Ventricular))) OR (Hypertrophy, Left Ventricular[MeSH Terms])) OR ((Left Ventricular Hypertrophy) OR (Ventricular Hypertrophy, Left))) OR (Ventricular Function, Right[MeSH Terms])) OR ((Right Ventricular Function) OR (Function, Right Ventricular))) OR (Ventricular Dysfunction, Right[MeSH Terms])) OR ((Right Ventricular Dysfunction) OR (Dysfunction, Right Ventricular))) AND ((Echocardiography) OR (Cardiac Magnetic Resonance Imaging) OR (Cardiovascular Magnetic Resonance))) OR (((Coronary Arteriosclerosis) OR (Coronary Artery Calcification) OR (Coronary Artery Calcium) OR (Coronary Plaque)) AND ((Computed Tomography) OR (Computed Tomographic Angiography) OR (Intravascular Ultrasound) OR (Optical Frequency Domain Imaging))))

limit to English, Adult: 19+ years, Humans. 2005-2020
