# Supplementary material for: Obstructive sleep apnea increases the risk of cardiovascular damage: a systematic review and meta-analysis of imaging studies
Source: Syst Rev. 2021 Jul 30;10:212. doi: 10.1186/s13643-021-01759-6 (PMC8325188; doi:10.1186/s13643-021-01759-6)
Supplement: Supplementary file 1 — Additional file 1: Table S1 A example of search strategies. Table S2 Equations. Table S3 Certainty of evidence. Figure S1 Forest plot. Figure S2 Funnel plot. Figure S3 Sensitivity plot. [file 13643_2021_1759_MOESM1_ESM.zip › Table S3 Certainty of evidenceR1.docx]

# Table S3 Summary of findings table

| **Quality assessment** | | | | | | | **No of patients** | | **Effect** | | **Quality** |
| --- | --- | --- | --- | --- | --- | --- | --- | --- | --- | --- | --- |
|  |  |  |  |  |  |  |  |  |  |  |  |
| **No of studies** | **Design** | **Limitations** | **Inconsistency** | **Indirectness** | **Imprecision** | **Other considerations** | **OSA** | Control | **Relative (95% CI)** | **Absolute** |  |
| **coronary artery calcification score** | | | | | | | | | | | |
| 12 | observational studies | no serious limitations | serious^1^ | no serious indirectness | no serious imprecision | reduced effect ^1^ | - | - | - | not pooled | ÅÅOO LOW |
| **coronary plaque burden (assessed with: the presence of plaque, plaque volume)** | | | | | | | | | | | |
| 9 | observational studies | no serious limitations | no serious inconsistency | no serious indirectness | no serious imprecision | reduced effect ^1^ | - | - | - | not pooled | ÅÅÅO MODERATE |
|  |  |  |  |  |  |  |  |  |  |  |  |
| **left atrial diameter** | | | | | | | | | | | |
| 13 | observational studies | no serious limitations | no serious inconsistency | no serious indirectness | no serious imprecision | none | 1107 | 317 | - | SMD 0.385 higher (0.252 to 0.518 higher)^1^ | ÅÅOO LOW |
| **left atrium volume index** | | | | | | | | | | | |
| 6 | observational studies | no serious limitations | no serious inconsistency^1^ | no serious indirectness | no serious imprecision | reporting bias^1^ | 238 | 163 | - | SMD 0.307 higher (0.096 to 0.518 higher)^1^ | ÅOOO VERY LOW |
| **left ventricular end-systolic diameter** | | | | | | | | | | | |
| 24 | observational studies | no serious limitations | no serious inconsistency | no serious indirectness | no serious imprecision | none | 1526 | 620 | - | SMD 0.323 higher (0.223 to 0.422 higher) | ÅÅOO LOW |
| **left ventricular end-diastolic diameter** | | | | | | | | | | | |
| 18 | observational studies | no serious limitations | no serious inconsistency | no serious indirectness | no serious imprecision | none | 918 | 406 | - | SMD 0.126 higher (0.003 to 0.249 higher) | ÅÅOO LOW |
| **left ventricular mass** | | | | | | | | | | | |
| 7 | observational studies | no serious limitations | no serious inconsistency^1^ | no serious indirectness | no serious imprecision | none | 708 | 612 | - | SMD 0.558 higher (0.403 to 0.712 higher) | ÅÅOO LOW |
| **left ventricular mass index** | | | | | | | | | | |  |
| 23 | observational studies | no serious limitations | serious^1^ | no serious indirectness | no serious imprecision | none | 1515 | 641 | - | SMD 0.478 higher (0.242 to 0.714 higher) | ÅOOO VERY LOW |
| **interventricular septum diameter** | | | | | | | | | | | |
| 24 | observational studies | no serious limitations | serious^1^ | no serious indirectness | no serious imprecision | reporting bias^1^ | 1375 | 808 | - | SMD 0.471 higher (0.195 to 0.747 higher) | ÅOOO VERY LOW |
| **posterior wall diameter** | | | | | | | | | | | |
| 22 | observational studies | no serious limitations | serious^1^ | no serious indirectness | no serious imprecision | none | 1348 | 1047 | - | SMD 0.602 higher (0.328 to 0.875 higher) | ÅOOO VERY LOW |
| **left ventricular ejection fraction** | | | | | | | | | | | |
| 39 | observational studies | no serious limitations | serious^1^ | no serious indirectness | no serious imprecision | none | 2552 | 1737 | - | SMD -0.238 lower (-0.379 higher to -0.097 lower) | ÅOOO VERY LOW |
| **left ventricular myocardial performance index** | | | | | | | | | | | |
| 8 | observational studies | no serious limitations | serious^1^ | no serious indirectness | no serious imprecision | none^1^ | 512 | 385 | - | SMD 0.687 higher (0.371 to 1.004 higher) | ÅOOO VERY LOW |
| **right ventricular diameter** | | | | | | | | | | | |
| 15 | observational studies | no serious limitations | serious^1^ | no serious indirectness | no serious imprecision | none | 845 | 470 | - | SMD 0.725 higher (0.605 to 0.845 higher) | ÅOOO VERY LOW |
| **right ventricular myocardial performance index** | | | | | | | | | | | |
| 8 | observational studies | no serious limitations | no serious inconsistency | no serious indirectness | no serious imprecision | none | 303 | 346 | - | SMD 0.881 higher (0.487 to 1.274 higher) | ÅÅOO LOW |
| **tricuspid annular plane systolic excursion** | | | | | | | | | | | |
| 10 | observational studies | no serious limitations | serious^1^ | no serious indirectness | no serious imprecision | none | 435 | 419 | - | SMD -0.481 lower (-0.810 higher to -0.152 lower) | ÅOOO VERY LOW |
| **right ventricular fractional area change** | | | | | | | | | | | |
| 5 | observational studies | no serious limitations | no serious inconsistency | no serious indirectness | no serious imprecision | none | 762 | 234 | - | SMD -0.399 lower (-0.553 higher to -0.246 lower) | ÅÅOO LOW |
| **myocardial injury (assessed with: the presence of left ventricular scar, myocardial contractile reserve)** | | | | | | | | | | | |
| 2 | observational studies | no serious limitations | no serious inconsistency^1^ | no serious indirectness | no serious imprecision | reporting bias^1^ | - | - |  | not pooled | ÅOOO VERY LOW |
|  |  |  |  |  |  |  |  |  |  |  |  |

^1^ case-control
