# Supplementary figures and images for: Obstructive sleep apnea increases the risk of cardiovascular damage: a systematic review and meta-analysis of imaging studies
Source: Syst Rev. 2021 Jul 30;10:212. doi: 10.1186/s13643-021-01759-6 (PMC8325188; doi:10.1186/s13643-021-01759-6)

**Table S2 Equations**

M=(N1M1+N2M2)/(N1+N2)


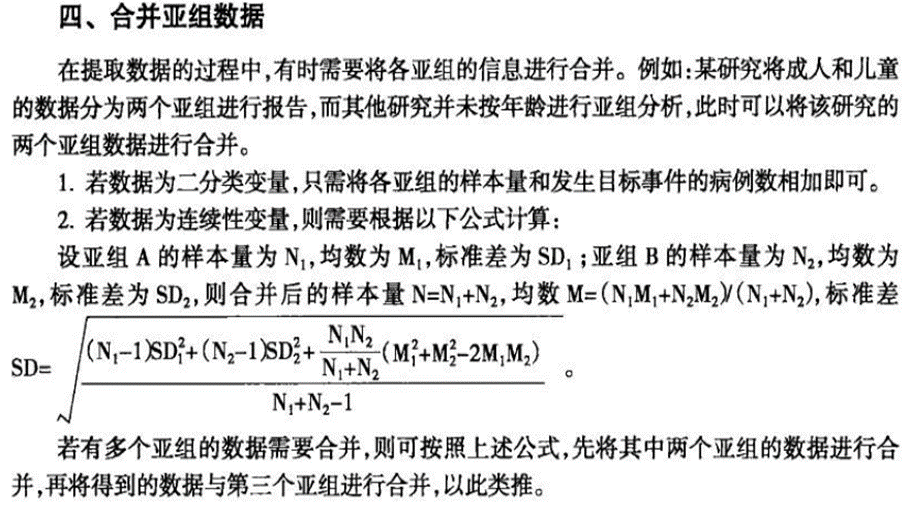

Supplement: Supplementary file 1 — Additional file 1: Table S1 A example of search strategies. Table S2 Equations. Table S3 Certainty of evidence. Figure S1 Forest plot. Figure S2 Funnel plot. Figure S3 Sensitivity plot. [file 13643_2021_1759_MOESM1_ESM.zip › Table S2 EquationsR1.docx]
